# Design, Synthesis and Biological Effects Studies of Novel EGFR Inhibitors Targeting Wild-Type and Mutant EGFR (EGFR-L858R and EGFR-L858R/T790M)

**DOI:** 10.1021/acsomega.6c00728

**Published:** 2026-06-18

**Authors:** Derya Osmaniye, Ümit Balıkçı, Berkant Kurban, Yusuf Özkay, Zafer Asım Kaplancıklı

**Affiliations:** † Department of Pharmaceutical Chemistry, Faculty of Pharmacy, 52944 Anadolu University, Eskişehir 26470, Turkey; ‡ Central Research Laboratory, Faculty of Pharmacy, Anadolu University, Eskişehir 26470, Turkey; § Faculty of Pharmacy, Anadolu University, Eskişehir 26470, Turkey; ∥ Institute of Graduate Education, Anadolu University, Eskişehir 26470, Turkey; ⊥ Department of Pharmaceutical Chemistry, Faculty of Pharmacy, Afyonkarahisar Health Sciences University, Afyonkarahisar 03030, Turkey; # Department of Pharmacy Services, Vocational School of Health Services, Bilecik Seyh Edebali University, Bilecik 11230, Turkey

## Abstract

Lung cancer remains one of the most significant global health challenges. Although EGFR inhibitors are actively employed in treatment, there is an urgent need for novel and effective inhibitors. In this context, a series of new EGFR inhibitors targeting both wild-type and mutant EGFR were designed and synthesized. The anticancer potentials of the synthesized derivatives were evaluated on A549 (lung cancer) and NIH/3T3 (healthy fibroblast) cell lines using the MTT method. Biological activity results revealed that the derivatives with 3,4-dichloro (**2i**) and 2,4-dichloro (**2j**) substitutions on the phenyl ring exhibited the highest potency in the series. Compound **2i** showed superior efficacy against A549 cells with an IC_50_ of 3.075 μM and a selective profile against healthy cells. Molecular docking studies (PDB: 4HJO, 2ITZ, 4I22) conducted to support the experimental data demonstrated that the active compounds were highly compatible with the ATP-binding pocket of EGFR. Structure–activity relationship (SAR) analyses showed that the specific halogen bonds formed by the dichloro derivatives with Met769/Met793 residues in the hinge region played a critical role in the activity enhancement. In enzyme inhibition tests, the success achieved by compound **2i** at the nM level with an IC_50_ = 0.096 μM against both the L858R and L858R-T790 M double mutant forms of EGFR confirmed the potential of this derivative to overcome clinical resistance mutations. In conclusion, the strong correlation between rational design, docking predictions, and biological activity results proves that the 3,4-dichloro (**2i**) modification is a key structural optimization in developing a next-generation EGFR inhibitor for the treatment of resistant lung cancer.

## Graphical abstract

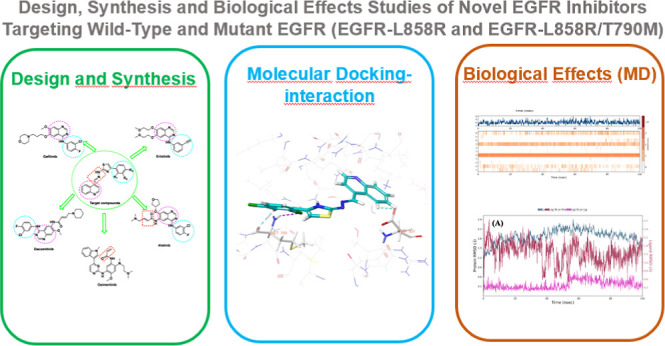

## 1 Introduction

Lung cancer is among the leading causes of cancer-related deaths worldwide in both women and men. Epidermal growth factor receptor (EGFR) is a critical receptor tyrosine kinase that facilitates the transduction of extracellular growth signals into cells and regulates cell proliferation, differentiation, and survival. Mutations leading to the overexpression or activation of EGFR have been associated with tumor development and progression in various solid tumors, particularly nonsmall cell lung cancer (NSCLC). For example, resistance to tubulin inhibitors in nonsmall cell lung cancer (NSCLC) cell lines such as A549, H1975, and PC9 is closely related to the overactivation of the EGFR signaling pathway. Pharmacological inhibition of EGFR may contribute to breaking down resistance to tubulin inhibitors in lung cancer cells by suppressing this aberrant signaling. Therefore, inhibition of EGFR is considered an important therapeutic strategy that suppresses tumor growth and can limit the aggressive course of the disease, especially in NSCLC and some subtypes of pancreatic cancer.

**Scheme 1:**
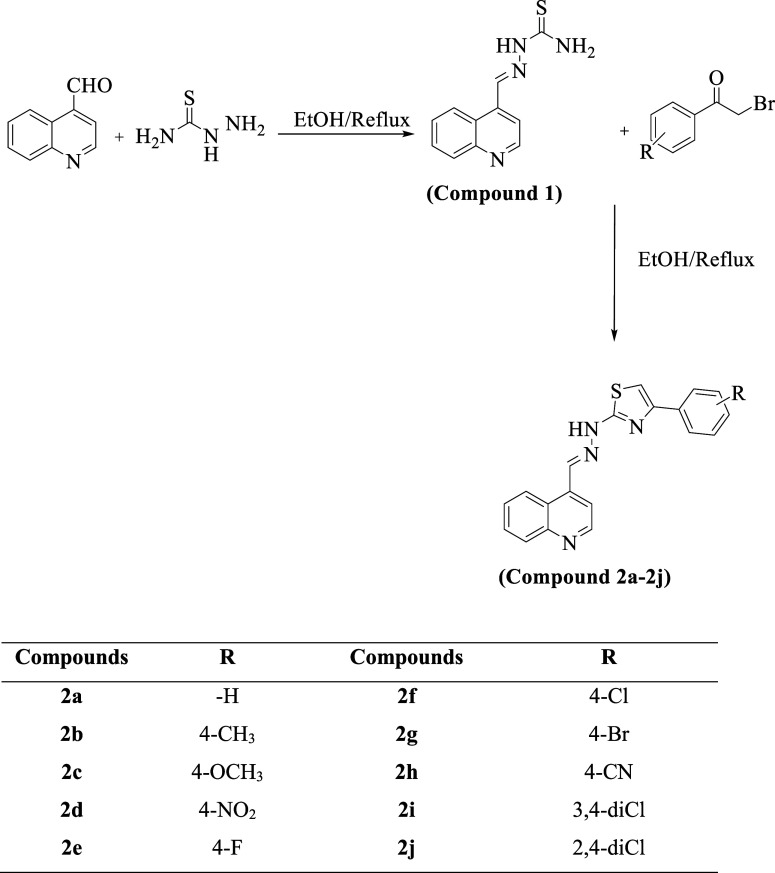
Synthesis Procedure of Compounds **2a**–**2j**

EGFR tyrosine kinase inhibitors (TKIs) are classified according to their selectivity for different forms of EGFR (wild-type and mutated variants). Erlotinib and gefitinib, first-generation FDA-approved TKIs, exhibit high inhibitory activity on wild-type EGFR and EGFR with the single mutation L858R. As treatment duration increases, an acquired mutation in EGFR, Thr790Met (T790M), can develop, leading to resistance to first-generation EGFR-TKIs. Although second-generation irreversible EGFR-TKIs such as afatinib and dacomitinib exert their effects by covalently binding to Cys797, the most effective clinical approach for T790M-mediated resistance has been achieved with third-generation inhibitors (osimertinib). Although the clinical efficacy of osimertinib is well-established and clearly defined, the development of acquired resistance mechanisms with long-term use is considered inevitable in most patients. Therefore, the development of fourth-generation EGFR-TKIs that can overcome the C797S-mediated resistance mechanism while maintaining high selectivity against mutant EGFR stands out as a significant unmet clinical need.^1–10^

In this study, ring variation was evaluated as a rational strategy in the novel drug design process to preserve pharmacophoric properties while reducing the development of acquired resistance. Accordingly, the use of quinoline cores instead of quinazoline cores was preferred. The quinazoline ring is an important pharmacophore in terms of anticancer activity and has been successfully incorporated into numerous anticancer agents developed for various targets, as widely reported in the literature.^11–20^ In addition, literature data on EGFR enzyme inhibition by compounds containing a thiazole ring^21–27^ suggest that hybrid molecules formed by combining these two pharmacophoric rings could enable the development of new derivatives with high efficacy.

The design of the compounds combines two powerful anticancer scaffolds: quinoline ring: found in many FDA-approved EGFR inhibitors (pelitinib), this unit fits well into hydrophobic pockets and allows for pi–pi stacking due to its planar structure. 2-Aminothiazole and hydrazone bridge: this hybrid structure provides flexibility to the molecule while also offering “donor–acceptor” sites capable of hydrogen bonding with the enzyme’s critical amino acids (Asp831/Asp800). In addition to classical hydrogen bonds, it also aims to establish “halogen bonds” with Met769/Met793 in the hinge region using chlorine and bromine atoms (Figure 1). The T790 M mutation narrows the pocket entry by converting threonine into a bulkier methionine. The design strategy aims to control volume by utilizing the rotational freedom of the phenyl ring via the hydrazone bridge, allowing the compound to “fit” into this narrowed pocket. In summary, this study employed a “pharmacophore coupling” strategy to develop next-generation therapeutic candidates for EGFR inhibition. The hydrophobic character of the quinoline ring was combined with the hydrogen bonding ability of the hydrazone-thiazole skeleton. During the design phase, dichloro (**2i** and **2j**) modifications on the phenyl ring were rationally positioned to establish specific halogen bonds with hinge site residues (Met769/Met793). This strategy resulted in compounds exhibiting high affinity not only for wild-type EGFR but also for clinically resistant L858R and T790 M mutations.

**Figure 1:**
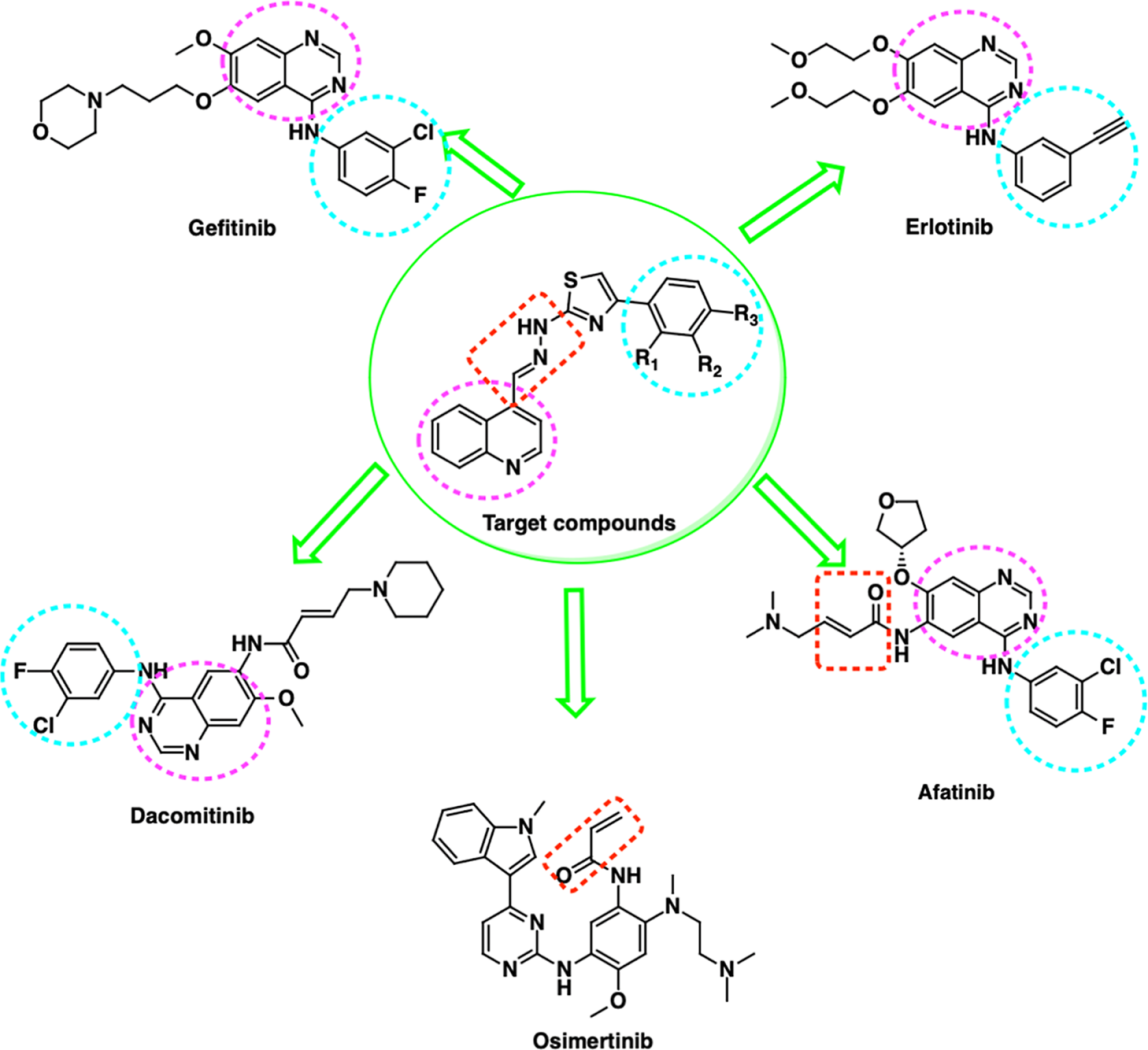
Investigation of common pharmacophoric regions of target compounds and kinase inhibitors.

## 2 Materials and Methods

### 2.1 Chemistry

All reagents were purchased from commercial suppliers and no purification was performed before use. Melting points (M.N.) were determined using the Mettler Toledo-MP90 Melting Point System without any correction. Characterization of the compounds was carried out using a 1H NMR DPX 300 FT-NMR spectrometer and a 13C NMR DPX 75 MHz spectrometer (Bruker Bioscience, USA). Mass spectrometers were recorded using the ESI technique and a LCMS-IT-TOF (Shimadzu, Kyoto, Japan) instrument (Figures S1–S30).

#### 2.1.1 Synthesis of 2-(Quinolin-4-ylmethylene)­hydrazine-1-carbothioamide (**1**)

Quinoline-4-carbaldehyde (0.002 mol, 0.314 g) was dissolved in absolute ethanol, followed by the addition of thiosemicarbazide (0.002 mol, 0.182 g). The resulting reaction mixture was heated under reflux for 12 h. The progress of the reaction was monitored using TLC, and the product precipitated as a solid within the reaction medium. The solid was collected by filtration and washed with cold ethanol before being dried.

#### 2.1.2 Synthesis of the Target Compounds

2-(Quinolin-4-ylmethylene)­hydrazine-1-carbothioamide (**1**) (0.001 mol, 0.230 g) was dissolved in absolute ethanol, and then phenacyl bromide derivatives (0.001 mol) was added. The mixture was refluxed for 12 h, during which the reaction progress was monitored via TLC. A solid product formed in the reaction medium, which was subsequently isolated by filtration, washed with cold ethanol, and dried.

##### 2.1.2.1 4-Phenyl-2-(2-(quinolin-4-ylmethylene)­hydrazineyl)­thiazole (**2a**)

Yield: 78%, mp 285.0–285.4 °C. ^1^H NMR (300 MHz, DMSO-*d*_6_): δ 7.33–7.37 (1H, m, Ar-H), 7.43–7.47 (2H, m, Ar-H), 7.56 (1H, s, thiazole), 7.90 (2H, d, *J* = 7.28 Hz, Ar-H), 7.98–8.02 (1H, m, Ar-H), 8.11–8.15 (1H, m, Ar-H), 8.20 (1H, d, *J* = 5.48 Hz, Ar-H), 8.25 (1H, d, *J* = 8.44 Hz, Ar-H), 8.76 (1H, d, *J* = 8.64 Hz, Ar-H), 8.79 (1H, s, –CH), 9.18 (1H, d, *J* = 5.36 Hz, Ar-H). ^13^C NMR (75 MHz, DMSO-*d*_6_): δ 106.25, 118.29, 123.66, 125.09, 125.39, 126.07, 128.39, 129.21, 129.89, 133.88, 134.55, 135.61, 145.59. HRMS (*m*/*z*): [M + H]^+^ calcd for C_15_H_14_N_4_S, 331.0988; found, 331.1012.

##### 2.1.2.2 2-(2-(Quinolin-4-ylmethylene)­hydrazineyl)-4-(*p*-tolyl)­thiazole (**2b**)

Yield: 81%, mp 288.5–288–7 °C. ^1^H NMR (300 MHz, DMSO-*d*_6_): δ 2.34 (3H, s, –CH_3_), 7.25 (2H, d, *J* = 7.52 Hz, 1,4-disubstituebenzene), 7.48 (1H, s, Thiazole), 7.78 (2H, d, *J* = 7.36 Hz, Ar-H), 7.97–8.01 (1H, m, *J* = 7.54 Hz, Ar-H), 8.11–8.14 (1H, m, Ar-H), 8.19 (1H, d, *J* = 4.84 Hz, Ar-H), 8.24 (1H, d, *J* = 8.48 Hz, Ar-H), 8.75–8.78 (2H, m, Ar-H), 9.17 (1H, d, *J* = 4.68 Hz, Ar-H). ^13^C NMR (75 MHz, DMSO-*d*_6_): δ 21.30, 105.30, 118.27, 125.07, 125.40, 126.01, 129.76, 129.85, 131.95, 133.84, 134.90, 135.55, 135.64, 137.73, 144.79, 145.61, 160.30. HRMS (*m*/*z*): [M + H]^+^ calcd for C_20_H_16_N_4_S, 345.1168; found, 345.1156.

##### 2.1.2.3 4-(4-Methoxyphenyl)-2-(2-(quinolin-4-ylmethylene)­hydrazineyl)­thiazole (**2c**)

Yield: 79%, mp 277.6–277.9 °C. ^1^H NMR (300 MHz, DMSO-*d*_6_): δ 3.80 (3H, s, –CH_3_), 7.00 (2H, d, *J* = 7.51 Hz, Ar-H), 7.38 (1H, s, thiazole), 7.82 (2H, d, *J* = 7.67 Hz, Ar-H), 7.97–8.01 (1H, m, Ar-H), 8.11–8.14 (1H, m, Ar-H), 8.18–8.19 (1H, m, Ar-H), 8.24 (1H, d, *J* = 8.43 Hz, Ar-H), 8.75–8.78 (2H, m, Ar-H), 9.17 (1H, d, *J* = 5.33 Hz, Ar-H). ^13^C NMR (75 MHz, DMSO-*d*_6_): δ 55.65, 104.06, 114.56, 118.23, 125.07, 125.41, 127.44, 129.86, 133.87, 134.58, 135.58, 137.55, 140.96, 144.35, 145.57, 159.53. HRMS (*m*/*z*): [M + H]^+^ calcd for C_20_H_16_N_4_OS, 361.1118; found, 361.11119.

##### 2.1.2.4 4-(4-Nitrophenyl)-2-(2-(quinolin-4-ylmethylene)­hydrazineyl)­thiazole (**2d**)

Yield: 76%, mp >300 °C. ^1^H NMR (300 MHz, DMSO-*d*_6_): δ 7.93–7.97 (2H, m, *J* = 8.34 Hz, Ar-H), 8.05–8.08 (1H, m, Ar-H), 8.11 (1H, d, *J* = 4.64 Hz, Ar-H), 8.14 (1H, s, Ar-H), 8.16 (1H, s, Ar-H), 8.21 (1H, d, *J* = 8.36 Hz, Ar-H), 8.31 (2H, d, *J* = 7.97 Hz, Ar-H), 8.73–8.76 (2H, m, Ar-H), 9.14 (1H, d, *J* = 4.76 Hz, Ar-H). ^13^C NMR (75 MHz, DMSO-*d*_6_): δ 110.78, 118.79, 124.68, 125.06, 125.23, 126.94, 129.58, 134.28, 138.90, 139.88, 140.70, 142.48, 146.90, 147.52, 149.50, 161.59. HRMS (*m*/*z*): [M + H]^+^ calcd for C_19_H_13_N_5_O_2_S, 376.0863; found, 376.085.

##### 2.1.2.5 4-(4-Fluorophenyl)-2-(2-(quinolin-4-ylmethylene)­hydrazineyl)­thiazole (**2e**)

Yield: 77%, mp >300 °C. ^1^H NMR (300 MHz, DMSO-*d*_6_): δ 7.26–7.30 (2H, m, *J* = 8.20 Hz, Ar-H), 7.52 (1H, s, Thiazole), 7.92–7.96 (3H, m, Ar-H), 8.05–8.11 (2H, m, *J* = 6.50 Hz, Ar-H), 8.26 (1H, d, *J* = 8.16 Hz, Ar-H), 8.74 (1H, d, *J* = 8.64 Hz, Ar-H), 8.78 (1H, s, Ar-H), 9.11–9.13 (1H, m, Ar-H). ^13^C NMR (75 MHz, DMSO-*d*_6_): δ 115.91, 116.20, 118.60, 122.22, 125.06, 125.19, 128.06, 128.16, 129.44, 130.25, 133.03, 133.54, 136.46, 136.79, 144.30, 146.85, 160.43. HRMS (*m*/*z*): [M + H]^+^ calcd for C_19_H_13_N_4_FS, 349.0918; found, 349.0907.

##### 2.1.2.6 4-(4-Chlorophenyl)-2-(2-(quinolin-4-ylmethylene)­hydrazineyl)­thiazole (**2f**)

Yield: 82%, mp 236.8–237.3 °C. ^1^H NMR (300 MHz, DMSO-*d*_6_): δ 7.50 (2H, d, *J* = 8.53 Hz, Ar-H), 7.62 (1H, s, thiazole), 7.91 (2H, m, *J* = 8.47 Hz, Ar-H), 7.97–8.02 (1H, m, Ar-H), 8.10–8.15 (1H, m, Ar-H), 8.19 (1H, d, *J* = 5.56 Hz, Ar-H), 8.25 (1H, d, *J* = 8.50 Hz, Ar-H), 8.75 (1H, d, *J* = 8.59 Hz, Ar-H), 8.79 (1H, s, Ar-H), 9.18 (1H, d, *J* = 5.54 Hz, Ar-H). ^13^C NMR (75 MHz, DMSO-*d*_6_): δ 107.07, 118.34, 123.62, 125.10, 125.38, 127.79, 129.22, 129.92, 132.78, 133.46, 133.91, 135.70, 140.87, 145.57, 160.37. HRMS (*m*/*z*): [M + H]^+^ calcd for C_19_H_13_N_4_SCl, 365.0622; found, 365.0624.

##### 2.1.2.7 4-(4-Bromophenyl)-2-(2-(quinolin-4-ylmethylene)­hydrazineyl)­thiazole (**2g**)

Yield: 79%, mp 296.5–297.7 °C. ^1^H NMR (300 MHz, DMSO-*d*_6_): δ 7.50 (2H, d, *J* = 8.13 Hz, Ar-H), 7.62 (1H, s, Thiazole), 7.91 (2H, m, *J* = 8.09 Hz, Ar-H), 7.95–7.99 (1H, m, Ar-H), 8.08–8.11 (1H, m, Ar-H), 8.14–8.15 (1H, m, Ar-H), 8.23 (1H, d, *J* = 8.43 Hz, Ar-H), 8.75 (1H, d, *J* = 8.80 Hz, Ar-H), 8.77 (1H, s, Ar-H), 9.16 (1H, d, *J* = 4.99 Hz, Ar-H). ^13^C NMR (75 MHz, DMSO-*d*_6_): δ 106.91, 118.51, 125.06, 125.29, 127.79, 129.22, 129.68. HRMS (*m*/*z*): [M + H]^+^ calcd for C_19_H_13_N_4_SBr, 409.0117; found, 409.0110.

##### 2.1.2.8 4-(2-(2-(Quinolin-4-ylmethylene)­hydrazineyl)­thiazol-4-yl)­benzonitrile (**2h**)

Yield: 75%, mp >300 °C. ^1^H NMR (300 MHz, DMSO-*d*_6_): δ 7.86 (1H, s, thiazole), 7.91 (2H, d, *J* = 7.70 Hz, Ar-H), 7.96–7.99 (1H, m, Ar-H), 8.06–8.12 (3H, m, Ar-H), 8.15–8.16 (1H, m, Ar-H), 8.23 (1H, d, *J* = 8.38 Hz, Ar-H), 8.74 (1H, d, *J* = 8.47 Hz, Ar-H), 8.78 (1H, s, Ar-H), 9.17 (1H, d, *J* = 4.83 Hz, Ar-H). ^13^C NMR (75 MHz, DMSO-*d*_6_): δ 107.20, 118.28, 121.40, 123.47, 125.08, 125.38, 128.07, 129.97, 132.13, 133.77, 134.01, 135.57, 140.66, 145.46, 145.73, 160.23. HRMS (*m*/*z*): [M + H]^+^ calcd for C_20_H_13_N_5_S, 356.0964; found, 356.0948.

##### 2.1.2.9 4-(3,4-Dichlorophenyl)-2-(2-(quinolin-4-ylmethylene)­hydrazineyl)­thiazole (**2i**)

Yield: 80%, mp >300 °C. ^1^H NMR (300 MHz, DMSO-*d*_6_): δ 7.70 (1H, m, *J* = 8.32 Hz, Ar-H), 7.76 (1H, s, Ar-H), 7.87 (1H, m, *J* = 8.12 Hz, Ar-H), 7.94–7.98 (1H, m, Ar-H), 8.06–8.13 (3H, m, Ar-H), 8.21 (1H, d, *J* = 8.04 Hz, Ar-H), 8.73–8.75 (2H, m, Ar-H), 9.15–9.15 (1H, m, Ar-H). ^13^C NMR (75 MHz, DMSO-*d*_6_): δ 108.25, 118.72, 125.03, 125.26, 126.13, 127.71, 129.59, 131.46, 132.07, 133.21, 134.93, 135.31, 136.58, 142.31, 146.62, 160.08. HRMS (*m*/*z*): [M + H]^+^ calcd for C_19_H_12_N_4_SCl_2_, 399.0232; found, 399.0247.

##### 2.1.2.10 4-(2,4-Dichlorophenyl)-2-(2-(quinolin-4-ylmethylene)­hydrazineyl)­thiazole (**2j**)

Yield: 74%, mp 263.0–264.5 °C. ^1^H NMR (300 MHz, DMSO-*d*_6_): δ 7.51 (1H, d, *J* = 7.97 Hz, Ar-H), 7.60 (1H, s, Ar-H), 7.90–7.93 (3H, m, Ar-H), 8.01–8.05 (2H, m, Ar-H), 8.22 (1H, d, *J* = 8.16 Hz, Ar-H), 8.72–8.75 (2H, m, Ar-H), 9.10 (1H, d, *J* = 4.27 Hz, Ar-H). ^13^C NMR (75 MHz, DMSO-*d*_6_): δ 107.42, 118.76, 123.32, 125.21, 125.85, 127.79, 129.21, 129.30, 132.68, 136.63, 141.78, 147.16, 148.58, 162.62. HRMS (*m*/*z*): [M + H]^+^ calcd for C_19_H_12_N_4_SCl_2_, 399.0232; found, 399.0245.

### 2.2 Cytotoxicity Assay

The colorless 3-(4,5-dimethylthiazol-2-yl)-2,5-diphenyltetrazolium salt forms the basis of the MTT test for assessing the metabolic activity of living cells. This compound undergoes reduction to form the purple formazan product, and cell viability can be determined spectroscopically by the color change. A 24 h MTT test was performed using the healthy NIH3T3 cell line and the A549 adenocarcinomic human alveolar basal epithelial cell line. It was carried out as previously reported by our team.^28–30^

### 2.3 EGFR, EGFR-L858R and EGFR-L858R-T790 M Enzyme Inhibition Assay

EGFR tyrosine kinase inhibitor activities were evaluated using enzyme inhibition assays performed according to the manufacturer’s protocol. For this purpose, the EGFR Kinase Assay Kit (BPS Bioscience, San Diego, CA, USA; Cat. No: 40321), EGFR (L858R) Kinase Assay Kit (BPS Bioscience, San Diego, CA, USA; Cat. No: 40324), and EGFR (T790M/L858R) Kinase Assay Kit (BPS Bioscience, San Diego, CA, USA; Cat. No: 40322) were used. These kits utilize a Kinase-Glo MAX-based method that allows for the measurement of recombinant EGFR enzyme activity for inhibitor screening and profiling. Changes in enzyme activity were monitored by adding test compounds to the reaction mixture at different concentrations; signal measurements were performed on a microplate reader at the end of incubation as recommended by the manufacturer. Enzyme activity was calculated as a percentage of inhibition compared to a control group, and IC_50_ values were determined using nonlinear regression on concentration–response curves.^31–33^

### 2.4 Molecular Docking

In this study, a structure-based in silico docking method was applied to determine the interactions and binding sites of molecules coded **2i** and **2j**, which exhibit the strongest activity against AChE enzyme among the compounds formed. Protein–ligand interactions were studied on the crystal structure of EGFR, EGFR-L858R, EGFR-L858R-T790 M enzymes, PDB: 4HJO, PDB: 2ITZ, PDB: 4I22, respectively.^34–38^ First, the crystal structure was prepared with the “Protein Preparation Wizard”^39^ using Schrödinger Suite 2015 Update 2 software. This structure was edited following the necessary protocols to make it suitable for docking studies. Atomic potential charges and bond lengths of charged amino acids under environmental conditions were calculated using the OPLS 2005 force field. Compounds were made suitable for docking analysis using the LigPrep 3.8 ^40^ module and a docking grid was created using the Glide 7.1 ^41^ software. The same module was also used for SP (single precision) docking studies.

### 2.5 Molecular Dynamic Studies

For compound **2i** and **2j**, molecular dynamics (MD) simulations—which are regarded as a crucial computational tool to assess the time-dependent stability of a ligand at an active site for a drug–receptor complex were carried out. As previously stated, MD experiments were carried out for 100 ns. After the system setup was finished, the settings were used to conduct the MD simulation. The figures for root-mean-square deviation (rmsd), root-mean-square fluctuation (RMSF), and radius of gyration (*R*_g_) were determined by the Desmond application.^42–47^

## 3 Results and Discussions

### 3.1 Chemistry

The synthesis of 2-(quinolin-4-ylmethylene)­hydrazine-1-carbothioamide (**1**) was carried out from quinoline-4-carbaldehyde and thiosemicarbazide. Then, the target compounds were obtained with 2-(quinolin-4-ylmethylene)­hydrazine-1-carbothioamide (**1**) and appropriate phenacyl bromide derivatives (Scheme 1).

### 3.2 Cytotoxicity Assay

Five of the ten synthesized compounds were found to be effective at IC_50_ values ≤100 μM against the A549 cell line. Furthermore, compounds **2i** and **2j** exhibited the highest activity with IC_50_ values of 3.075 ± 0.121 and 13.121 ± 0.817, respectively (Supporting Information Figure S31–S40) (Table 1).

**Table 1:** IC_50_ (μM) Values of Synthesized Compounds

compounds	A549 (μM)	NIH/3T3 (μM)
**2a**	185.168 ± 1.088	332.315 ± 2.871
**2b**	271.049 ± 3.001	354.790 ± 4.801
**2c**	38.050 ± 0.712	410.157 ± 5.111
**2d**	44.424 ± 0.989	433.543 ± 3.709
**2e**	189.160 ± 2.789	>1000
**2f**	250.001 ± 3.891	>1000
**2g**	59.252 ± 1.701	97.796 ± 1.097
**2h**	121.288 ± 2.077	>1000
**2i**	3.075 ± 0.121	9.848 ± 0.121
**2j**	13.191 ± 0.817	85.884 ± 0.916

### 3.3 EGFR, EGFR-L858R and EGFR-L858R-T790 M Enzyme Inhibition Assay

The relevant results are presented in Table 2 and Figure 2. The inhibitory potential of the compounds showing activity in the MTT analysis was evaluated on both EGFR (WT) and its single-mutated (EGFR-L858R) and double-mutated (EGFR-L858R/T790M) forms. When the obtained data were compared, it was seen that both tested compounds showed a significant inhibitory effect on the EGFR-L858R mutation; furthermore, compound **2i** also exhibited inhibitory activity on the EGFR-L858R/T790 M double-mutated enzyme.

**Table 2:** % Inhibition and IC_50_ (μM) Values of Compounds **2i** and **2j** against EGFR, EGFR-L858R, EGFR-L858R-T790M

	**2i**			**2j**		
compounds	1000 μM	100 μM	IC_50_ (μM)	1000 μM	100 μM	IC_50_ (μM)
EGFR	78.199%	73.459%	0.879 ± 0.025	85.071%	75.592%	0.793 ± 0.017
EGFR-L858R	83.333%	79.710%	0.096 ± 0.002	88.586%	81.340%	0.061 ± 0.017
EGFR-L858R-T790M	86.956%	85.144%	0.055 ± 0.002	88.586%	81.341%	0.049 ± 0.017

**Figure 2:**
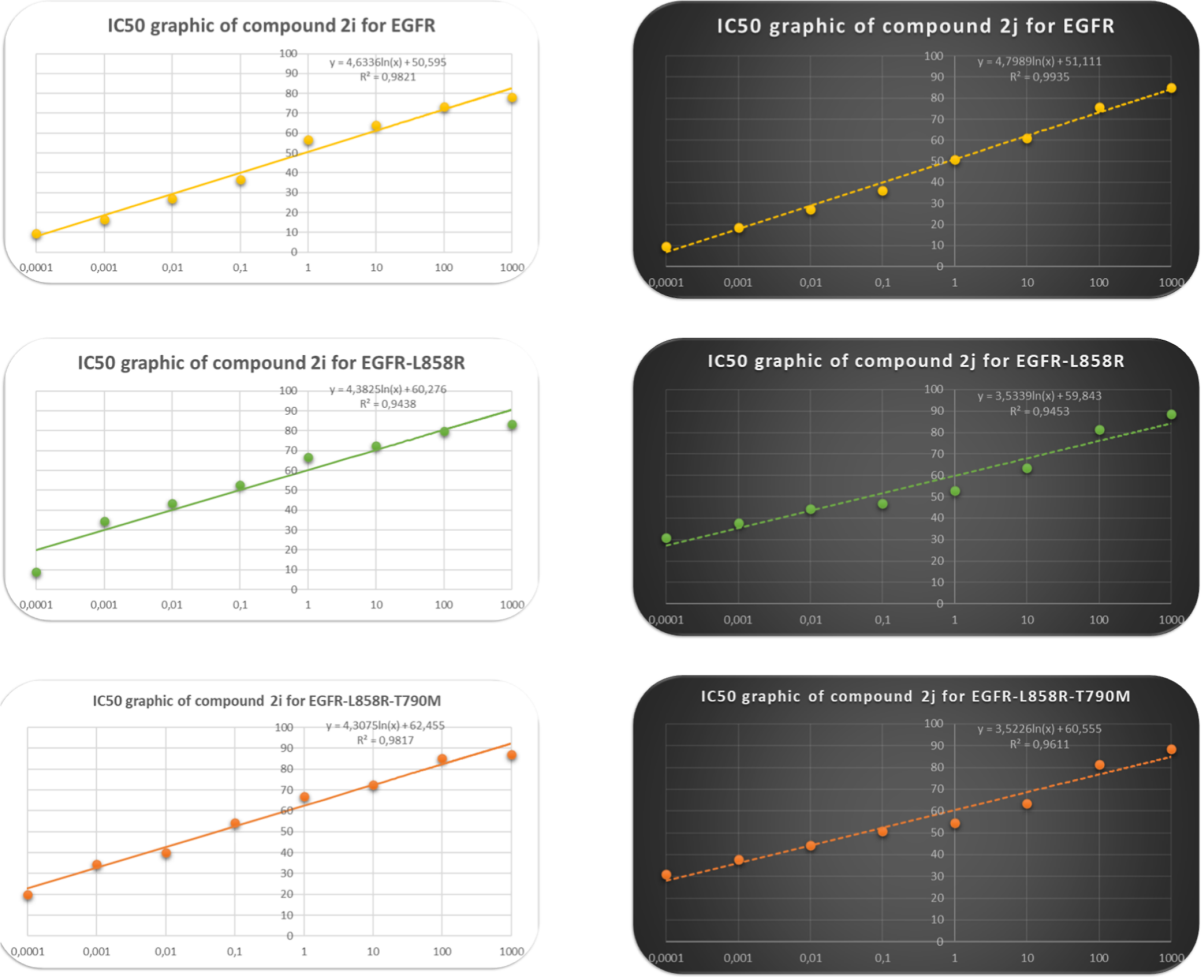
IC_50_ graphics of compounds **2i** and **2j** against EGFR, EGFR-L858R, EGFR-L858R-T790 M enzymes.

### 3.4 Molecular Docking Studies

Docking studies were performed using crystals with PDB IDs: 4HJO, 2ITZ, and 4I22. These enzyme crystals contain the EGFR, EGFR-L858R, and EGFR-L858R-T790 M mutations, respectively.

Figure 3 shows two-dimensional and three-dimensional images of the interactions of compounds **2i** and **2j** with the EGFR (PDB: 4HJO). The Cl atom at position 3 of compound **2i** formed a halogen bond with the amine group of Met769. Furthermore, the hydrazone nitrogen in the compound formed a hydrogen bond with Asp831, while the quinoxaline ring exhibited aromatic hydrogen bonding interactions with Ala719 and Asp831. The Cl atom in compound **2j** also formed a halogen bond with the amine group of Met769, and the 2,4-dichloro ring formed an aromatic hydrogen bond with the carbonyl of Met769. In addition, the quinoxaline ring of compound **2j** formed an aromatic hydrogen bond with Asp831.

**Figure 3:**
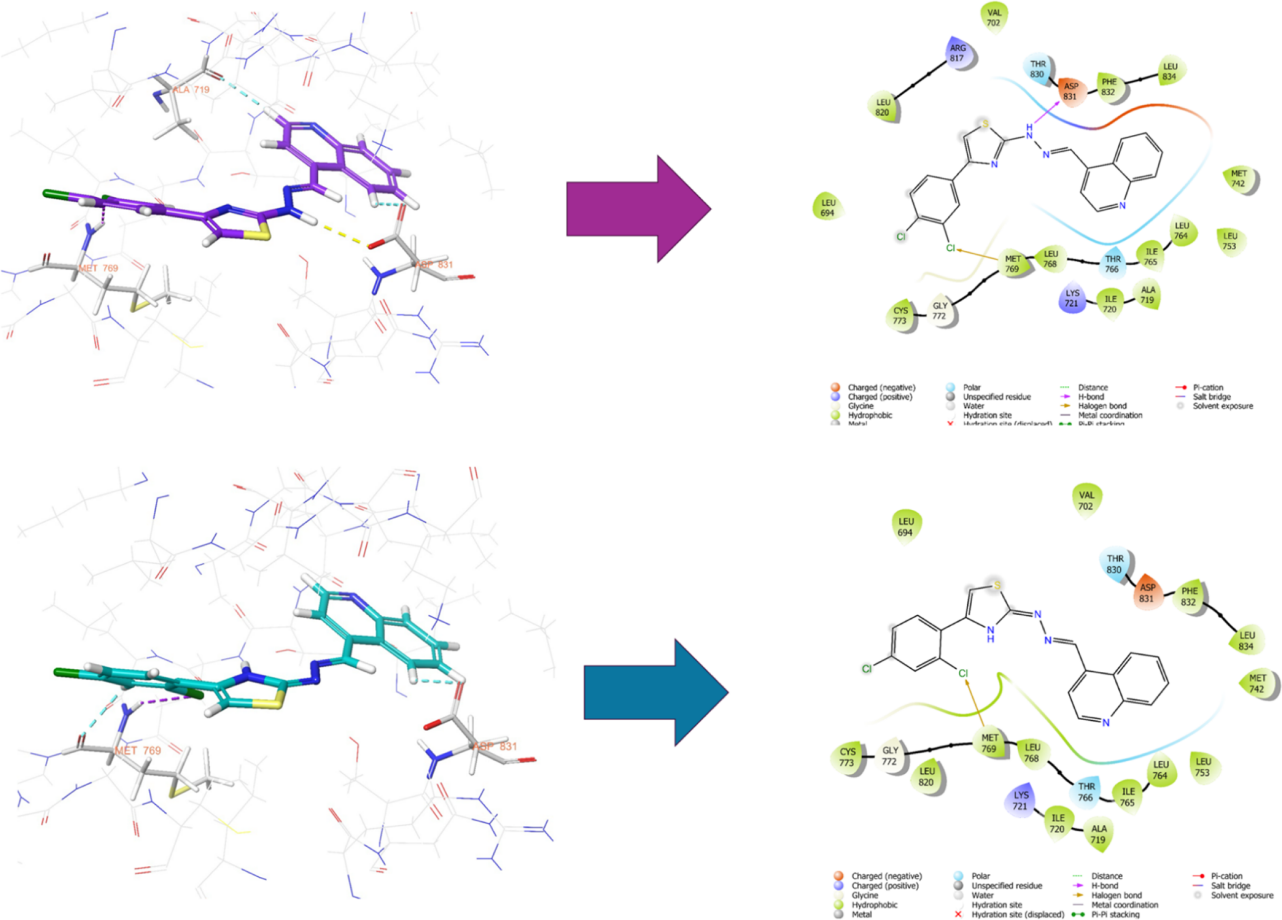
2D–3D docking poses of compounds **2i** and **2j** with EGFR enzyme (PDB: 4HJO).

Figure 4 presents the docking results performed with the EGFR-L858R (PDB: 2ITZ) crystal. The thiazole and hydrazone moieties of compound **2i** formed a double hydrogen bond with Met793. The Cl atom at position 3 of the same compound formed halogen bonds with Glu762 and Asp855. The thiazole ring of compound **2j** formed a hydrogen bond with Met793, and the Cl atom at position 4 exhibited a hydrogen bond interaction with Glu762. Furthermore, the quinoxaline ring of compound **2j** formed a cation–π interaction with Lys716.

**Figure 4:**
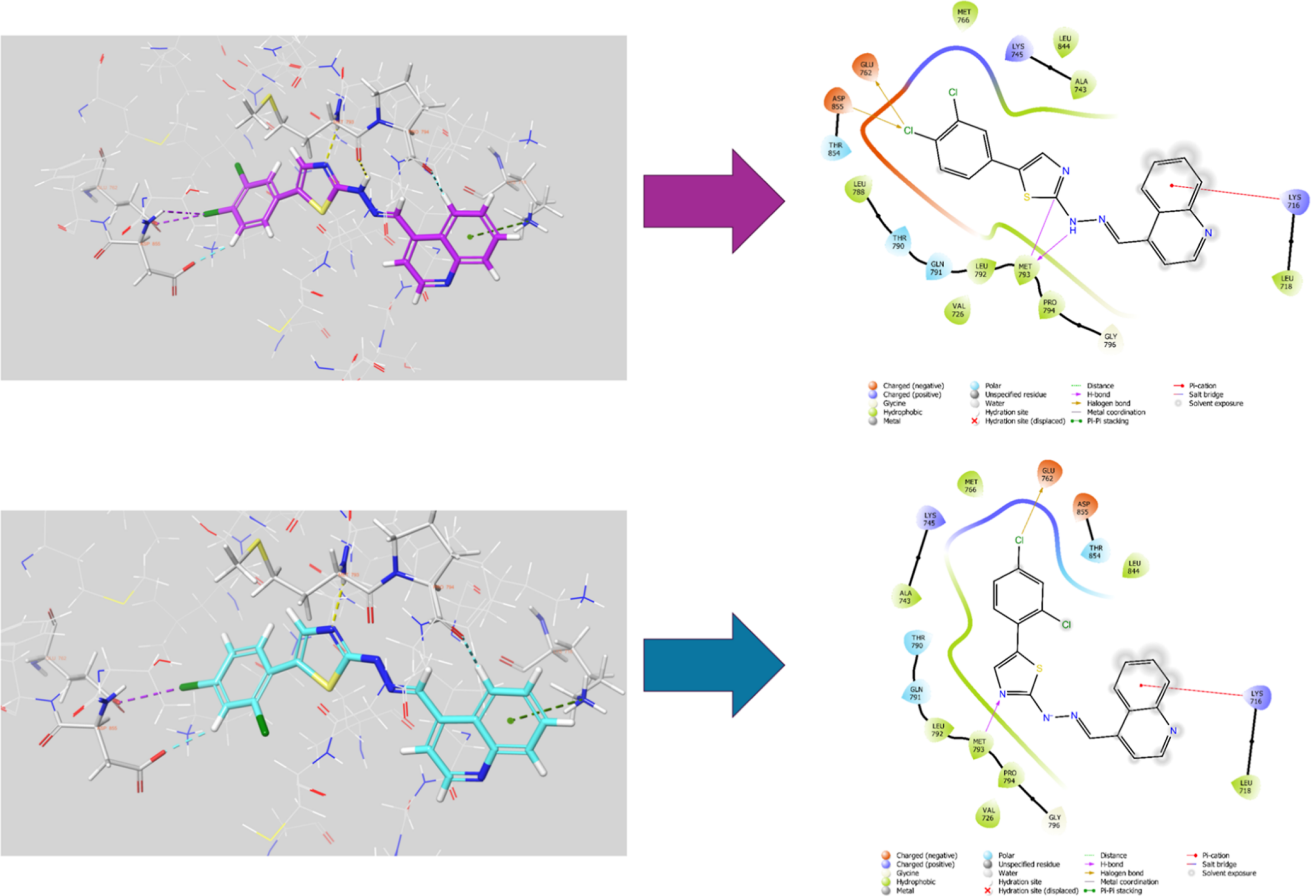
2D–3D docking poses of compounds **2i** and **2j** with EGFR-L858R enzyme (PDB: 2ITZ).

Figure 5 shows the docking results performed with the EGFR-L858R-T790 M (PDB: 4I22) crystal. The thiazole and hydrazone moieties of compound **2i** formed two hydrogen bonds with Met793. Similarly, compound **2j** showed the same interactions.

**Figure 5:**
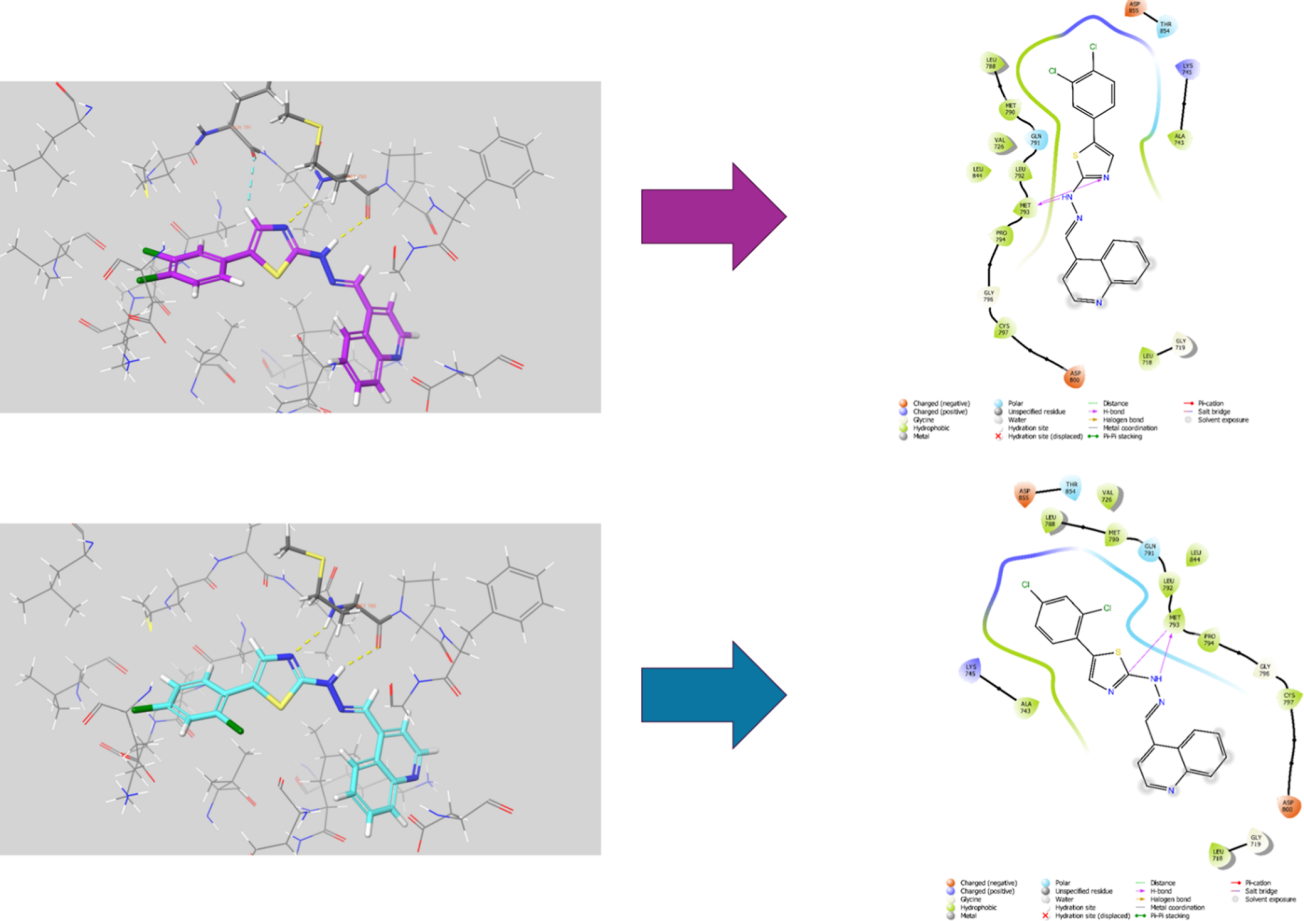
2D-3D docking poses of compounds **2i** and **2j** with EGFR-L858R-T790 enzyme (PDB: 4I22).

### 3.5 Molecular Dynamic Studies

Molecular docking studies provide guidance by predicting the possible binding modes of compounds with the target protein. Molecular dynamics (MD) simulations are necessary to confirm these results and understand dynamic stability. In this context, MD studies were performed for compounds **2i** and **2j** against the EGFR, EGFR-L858R, and EGFR-L858R-T790 M mutations.

EGFR (PDB ID: 4HJO), EGFR-L858R (PDB ID: 2ITZ), and EGFR-L858R-T790 M (PDB ID: 4I22) crystal structures were used in the simulations.

The complexes formed by each compound with the relevant enzyme were placed in the POPE membrane model, and simulations were run at 310.55 K for 100 ns.

In molecular dynamics simulations, rmsd (root mean square deviation) is a fundamental parameter used to evaluate the structural stability of a system over time. rmsd represents the root-mean-square deviation of atomic positions relative to a reference structure (usually the initial or crystal structure). Low rmsd values (≈1–3 Å) indicate that the system is in a stable conformation and that no major conformational changes have occurred. rmsd values for all complexes for which dynamic studies have been conducted are presented in Table 3.

**Table 3:** rmsd vs RMSF Graphics of Complex **2i +** 4HJO, **2j +** 4HJO, **2i +** 2ITZ, **2j +** 2ITZ, **2i +** 4I22, **2j +** 4I22

complex	rmsd (Å)	RMSF
**2i +** 4HJO	2.4	Leu694 (1.23 Å), Val702 (0.66 Å), Ala719 (0.49 Å), Ile720 (0.49 Å), Lys721 (0.51 Å), Met742 (0.76 Å), Leu753 (0.54 Å), Leu764 (0.49 Å), Thr766 (0.40 Å), Cys773 (0.55 Å), Arg817 (0.52 Å), Asn818 (0.50 Å), Leu820 (0.45 Å), Thr830 (0.45 Å), Asp831 (0.45 Å), Phe832 (0.61 Å), Leu834 (0.72 Å)
**2j +** 4HJO	2.25	Leu694 (0.82 Å), Ser696 (1.09 Å), Val702 (0.56 Å), Ala719 (0.60 Å), Lys721 (0.47 Å), Met742 (0.78 Å), Leu753 (0.52 Å), Leu764 (0.45 Å), Thr766 (0.53 Å), Leu768 (0.73 Å), Cys773 (0.50 Å), Arg817 (0.58 Å), Leu820 (0.46 Å), Asp831 (0.54 Å), Phe832 (0.85 Å), Leu834 (0.70 Å)
**2i +** 2ITZ	2.8	Lys716 (0.99 Å), Leu718 (0.91 Å), Phe723 (2.18 Å), Val726 (0.74 Å), Lys728 (0.77 Å), Ala743 (0.55 Å), Lys745 (0.65 Å), Gln791 (0.59 Å), Leu792 (0.63 Å), Met793 (0.57 Å), Cys797 (0.48 Å), Asp800 (0.59 Å), Val843 (0.42 Å)
**2j +** 2ITZ	2.4	Lys716 (0.93 Å), Leu718 (0.87 Å), Ser720 (1.12 Å), Phe723 (1.50 Å), Val726 (0.63 Å), Tyr727 (0.68 Å), Lys728 (0.69 Å), Ala743 (0.62 Å), Lys745 (0.57 Å), Met790 (0.55 Å), Gln791 (0.78 Å), Leu792 (0.83 Å), Met793 (1.07 Å), Pro794 (1.13 Å), Cys797 (0.68 Å), Leu799 (0.61 Å), Asp800 (0.77 Å), Arg803 (0.84 Å), Leu844 (0.57 Å)
**2i +** 4I22	2.4	Leu718 (0.97 Å), Val726 (0.69 Å), Lys728 (0.64 Å), Ala743 (0.45 Å), Met766 (0.46 Å), Leu792 (0.51 Å), Met793 (0.70 Å), Cys797 (0.69 Å), Asp800 (0.74 Å), Leu844 (0.64 Å), Met1002 (1.48 Å)
**2j +** 4I22	2.7	Lys716 (0.87 Å), Val717 (0.68 Å), Leu718 (0.86 Å), Val726 (0.69 Å), Lys728 (0.61 Å), Ala743 (0.43 Å), Leu792 (0.47 Å), Met793 (0.50 Å), Pro794 (0.69 Å), Phe795 (0.80 Å), Gly796 (0.78 Å), Cys797 (0.75 Å), Asp800 (0.92 Å), Arg803 (0.99 Å), Glu804 (1.14 Å), Leu844 (0.52 Å), Leu1001 (1.43 Å), Met1002 (1.74 Å)

On the other hand, RMSF (root mean square fluctuation) is a parameter that determines how much each atom or residue deviates from its mean position over time, allowing the identification of flexible and mobile regions in the system. rmsd reflects the overall stability of the entire structure, while RMSF indicates the flexibility of specific regions. RMSF values for all complexes are also presented in Table 3.

Figure 6 presents the dynamics results for the **2i +** 4HJO complex. Figure 7: it presents the dynamical results of the **2j +** 4HJO complex. Figure 8 presents the dynamical results of the **2i +** 2ITZ complex. Figure 9 presents the dynamical results of the **2j +** 2ITZ complex. Figure 10 presents the dynamical results of the **2i +** 4I22 complex. Figure 11 presents the dynamical results of the **2j +** 4I22 complex.

**Figure 6:**
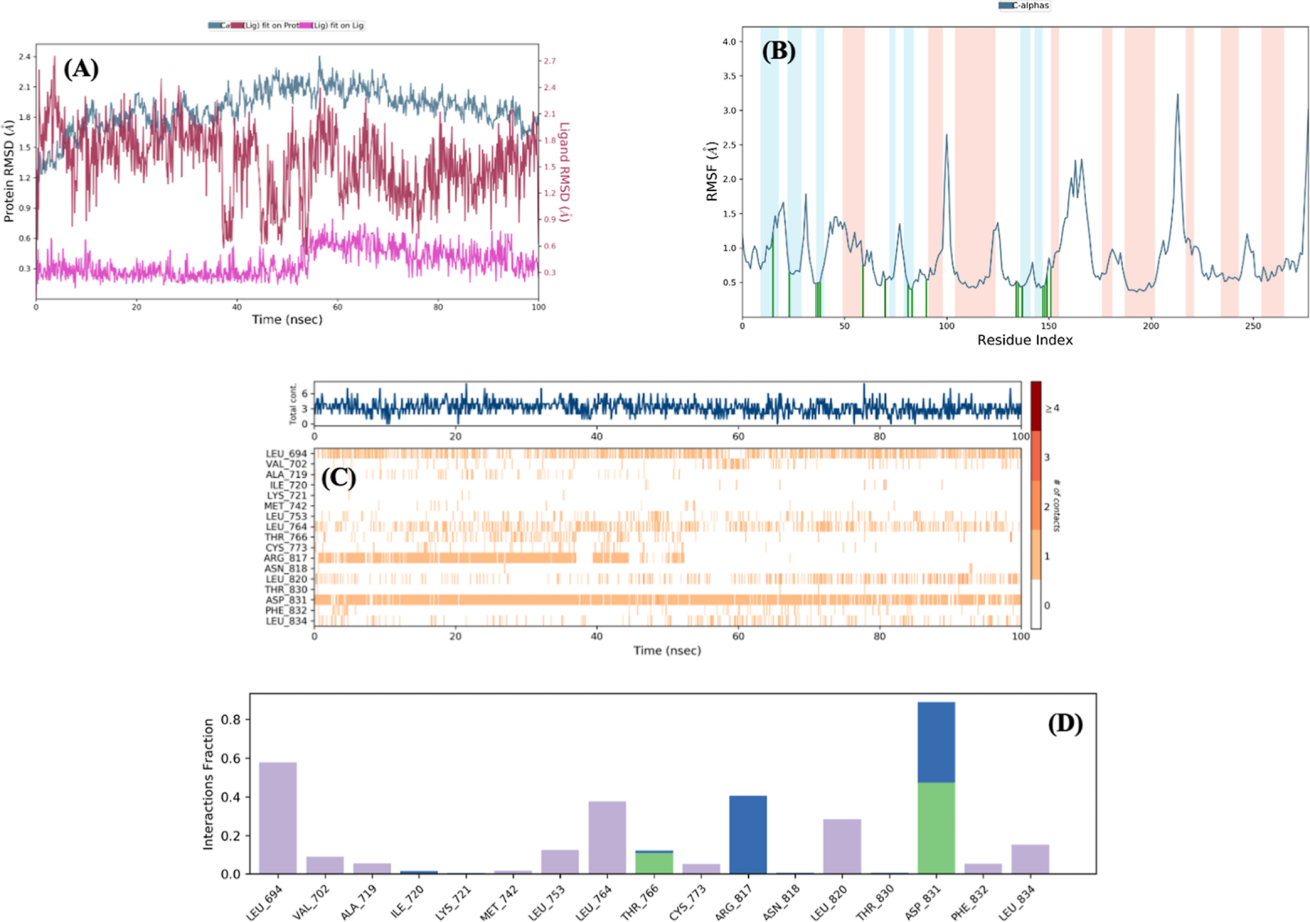
Molecular dynamics results of the **2i +** 4HJO complex. (A) rmsd parameters, (B) RMSF parameters, (C) time-dependent amino acid interactions, (D) types of interactions.

**Figure 7:**
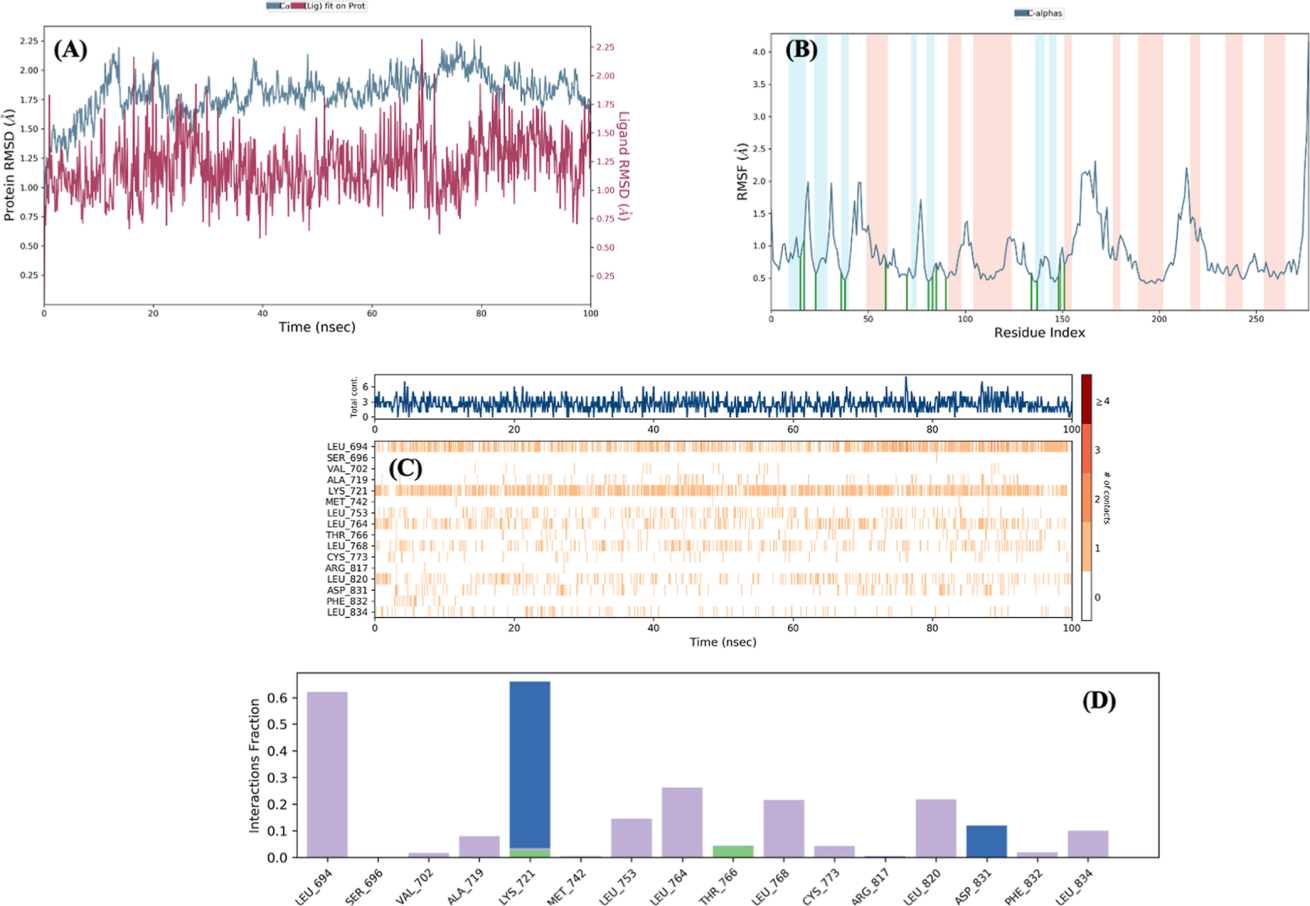
Molecular dynamics results of the **2j +** 4HJO complex. (A) rmsd parameters, (B) RMSF parameters, (C) time-dependent amino acid interactions, (D) types of interactions.

**Figure 8:**
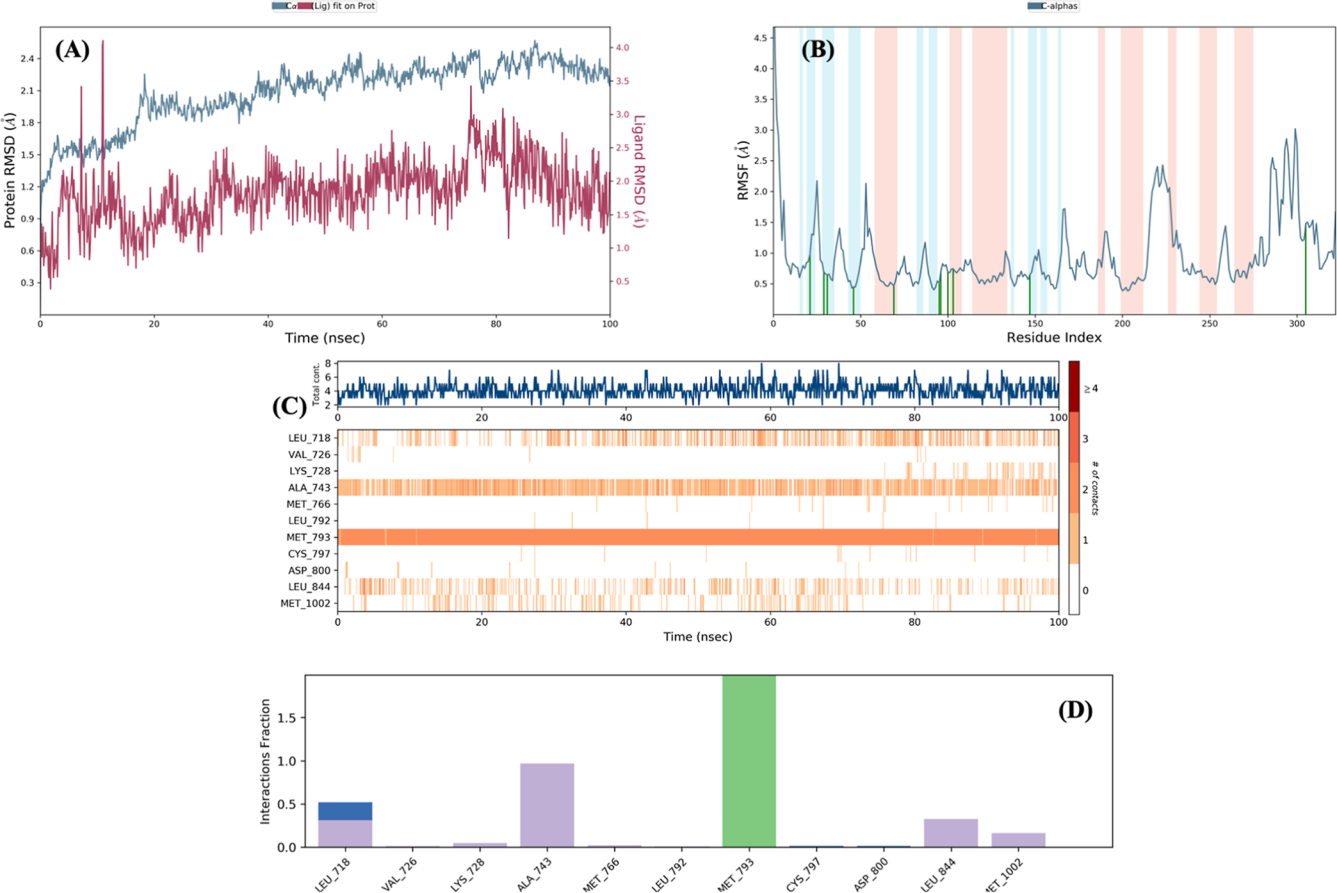
Molecular dynamics results of the **2i +** 2ITZ complex. (A) rmsd parameters, (B) RMSF parameters, (C) time-dependent amino acid interactions, (D) types of interactions.

**Figure 9:**
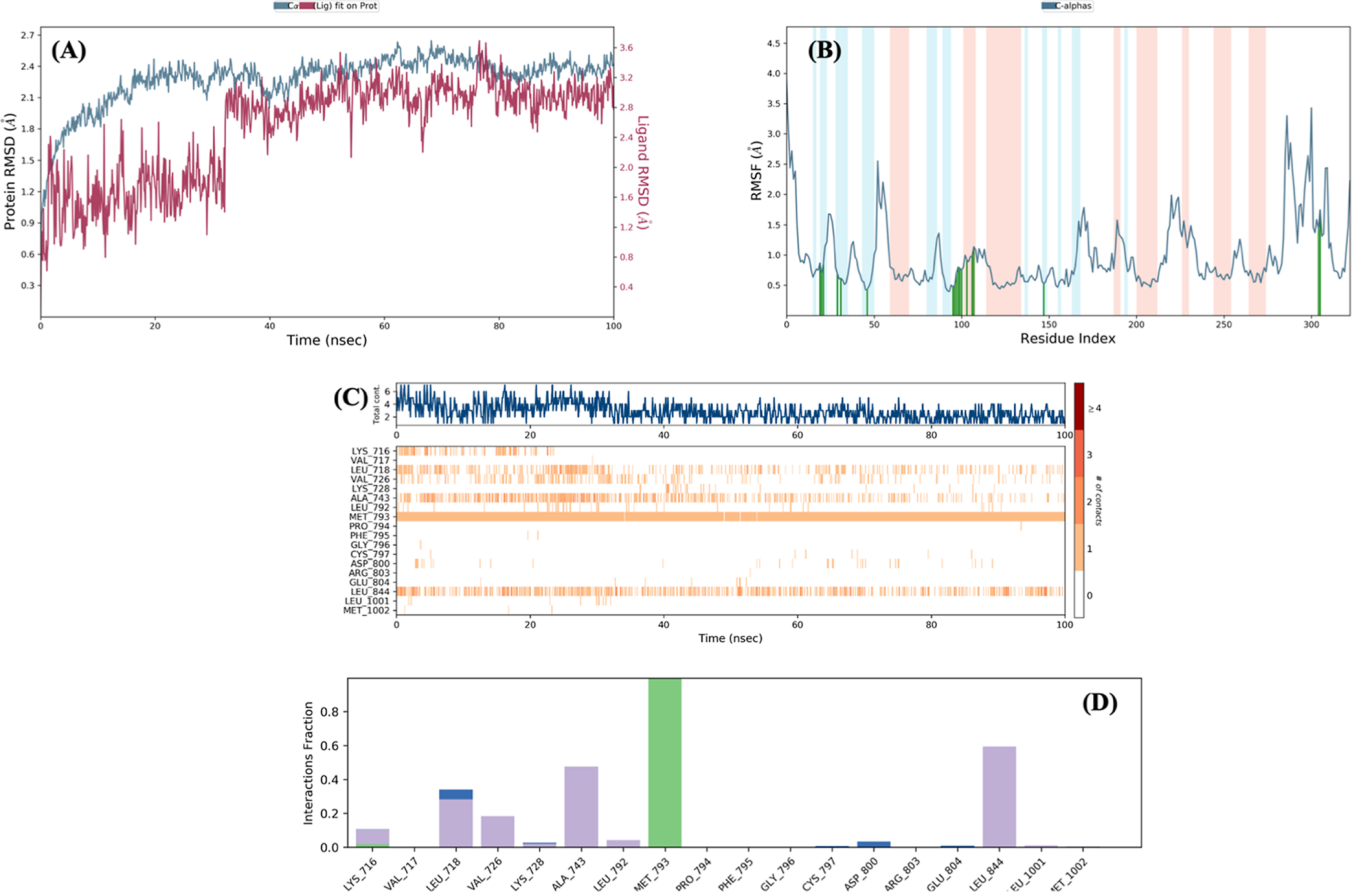
Molecular dynamics results of the **2j +** 2ITZ complex. (A) rmsd parameters, (B) RMSF parameters, (C) time-dependent amino acid interactions, (D) types of interactions.

**Figure 10:**
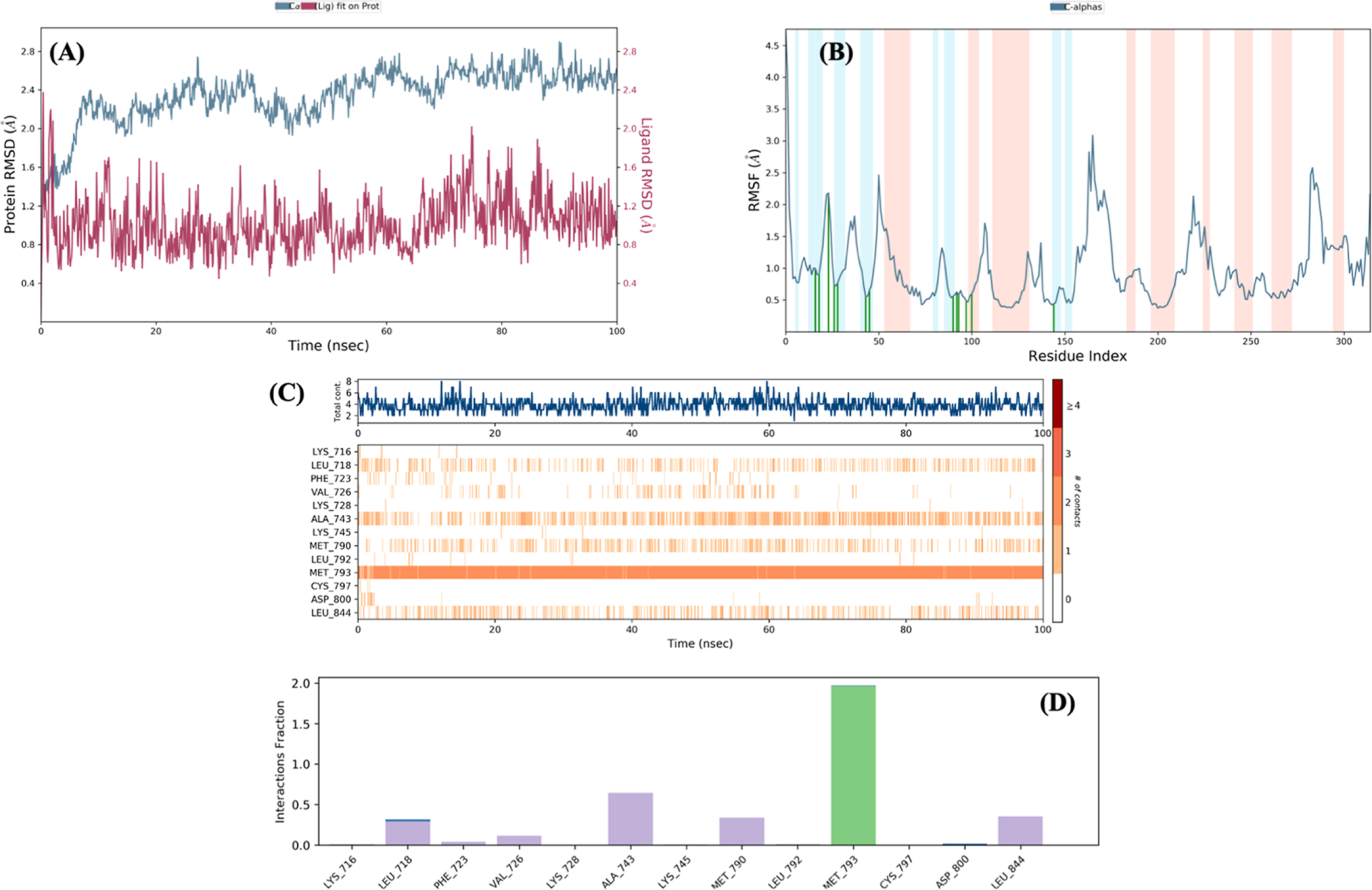
Molecular dynamics results of the **2i +** 4I22 complex. (A) rmsd parameters, (B) RMSF parameters, (C) time-dependent amino acid interactions, (D) types of interactions.

**Figure 11:**
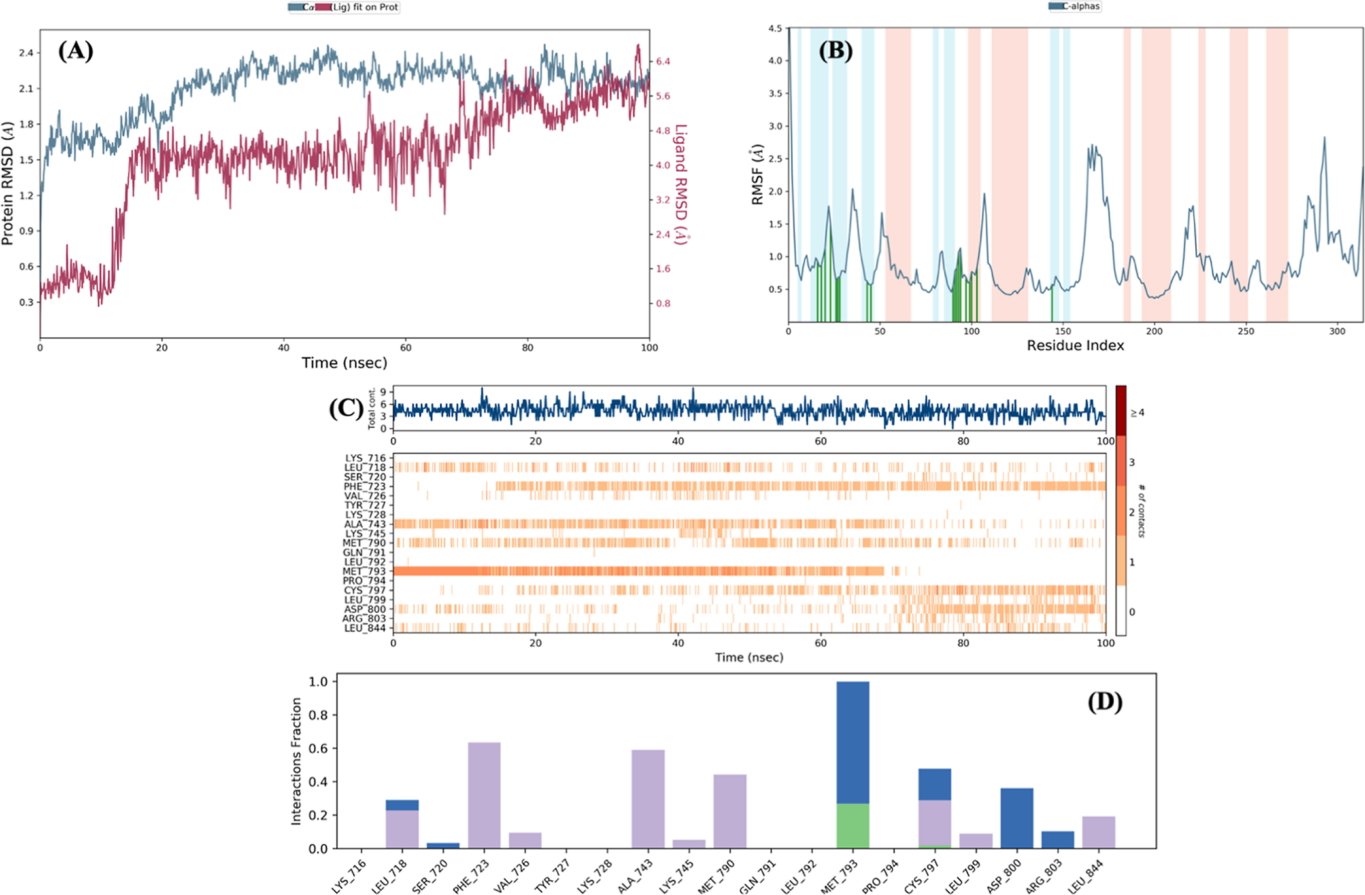
Molecular dynamics results of the **2j +** 4I22 complex. (A) rmsd parameters, (B) RMSF parameters, (C) time-dependent amino acid interactions, (D) types of interactions.

When dynamic results were examined, it was observed that the compounds did not exhibit significant inhibitory properties on the unmutated EGFR enzyme (PDB ID: 4HJO). This finding is also consistent with the in vitro results. The lack of interaction between both compounds and the amino acid Met790, which is critical for the binding site of the EGFR enzyme, structurally supports the negative results obtained in the in vitro studies.

On the other hand, the high activity observed in enzymes containing the L858R mutation alone or the L858R + T790 M double mutation is also supported by dynamic study results.

Figure 8C shows the interactions between compound **2i** and the EGFR-L858R enzyme. For this complex, a continuous interaction was observed with the amino acid Met793, located in the ATP-binding pocket (hinge region). This interaction is mediated by both the thiazole ring and the amine group attached to the thiazole, a typical binding pattern conserved in nearly all potent inhibitors. Furthermore, the same compound showed a continuous interaction with Ala743, which contributes significantly to the stabilization of the hydrophobic core.

Figure 9C shows the interactions between compound **2j** and the EGFR-L858R enzyme. Here, a continuous interaction was observed with Met793, and residues Ala743 and Leu844 supported hydrophobic contacts with the aromatic ring of the ligand.

Figure 10C shows the interactions between compound **2i** and the enzyme with the EGFR-L858R + T790 M mutation. A stable interaction with Met793 in the hinge region was maintained for 100 ns. Furthermore, continuous contact with Met790, located near the gatekeeper, was observed throughout the simulation. This mutation widens the back of the pocket, making it more hydrophobic; therefore, it causes steric repulsion in first-generation inhibitors, while third-generation inhibitor scaffolds accommodate this region through van der Waals or π-alkyl interactions.

Figure 11C shows the interactions between compound **2j** and the EGFR-L858R + T790 M enzyme. A continuous interaction with Met793 in the hinge region was observed for approximately 70 ns, after which time this interaction diminished, giving way to contacts with Cys797. The H-bond observed at Cys797 is a direct binding site in covalent inhibitors. Even if covalent binding did not occur, the close and continuous contact with this residue indicated high binding stability. Furthermore, interactions with Met790 lasting 100 ns significantly contributed to the stability of the complex.

Dynamic simulations showed that the Met793 interaction in both the EGFR-L858R and EGFR-L858R + T790 M complexes occurred through the thiazole ring. Therefore, the thiazole ring system can be considered the main pharmacophoric region responsible for the compounds’ activity. The Met790 interactions observed in the EGFR-L858R + T790 M enzyme were mediated by the compounds’ dichlorophenyl rings. This suggests that bulky ring systems hinder placement in the narrow pocket of wild-type EGFR, while providing a more suitable fit in the wide, hydrophobic pocket with the T790 M mutation.

Consequently, compounds **2i** and **2j** exhibit mutation-selective inhibitory profiles. This means that these compounds inhibit only mutant forms of EGFR without oversuppressing normal EGFR (and therefore with less toxicity and fewer dermatological side effects). This is a feature specifically targeted for second- and third-generation EGFR inhibitors (afatinib and osimertinib). Clinically, such compounds are expected to exhibit high efficacy in cancer cells carrying EGFR mutations, such as resistant NSCLC, and minimal side effects in healthy cells.

### 3.6 Structure–Activity Relationship (SAR) and Molecular Docking Analysis

Biological test results and molecular docking studies on three different EGFR crystal structures (PDB: 4HJO, 2ITZ, 4I22) support the following structure–activity inferences: role of the central skeleton: the conserved hydrogen bond formed by all active derivatives with Asp831 (wild type) or Asp800 (mutant) residues via the hydrazone NH group is a fundamental structural requirement for the compounds to be properly oriented to the ATP binding pocket. Dichloro effect and galogen bonds: the high activity exhibited by compounds **2i** and **2j** is directly related to the specific halogen bonds (C–Cl···O=C) formed by the chlorine atoms in the phenyl ring with the Met769/Met793 residues in the hinge region. When examined in terms of tolerance of resistance mutations (T790M), the 4I22 docking results show that the dichloro derivatives do not enter steric conflict with the gatekeeper mutation Met790 side chain. It has been confirmed that it can settle deep into the pocket. This rationally supports the experimental success of compound **2i** at the nM level on the double mutant enzyme. Regarding the importance of the substituent position: the 3,4-dichloro (**2i**) sequence is approximately 4 times more active at the cellular level (A549) than the 2,4-dichloro (**2j**) sequence, indicating that the chlorine atoms in the meta and para positions fit better into hydrophobic pockets. When examined in terms of electronic and steric factors; the presence of bulky or electron-withdrawing groups such as 4-NO_2_ (**2d**) and 4-OCH_3_ (**2c**) in the phenyl ring maintains the activity to a certain extent, while the unsubstituted derivative (**2a**) gives weaker results, proving that modification of the aromatic ring is necessary. A complete correlation was found between the experimental activity data and molecular modeling studies. The 3,4-dichloro (**2i**) modification has been identified as the most promising structural optimization for next-generation inhibitor designs specifically targeting resistant EGFR mutations. Poses of all obtained compounds are presented in the Supporting Information file (Figures S41–S43).

## 4 Conclusion

The anticancer potential of the synthesized hydrazone-thiazole derivatives (**2a**–**j**) was determined by MTT assay on A549 lung cancer and NIH/3T3 healthy fibroblast cell lines. Furthermore, the most active derivatives in the series, **2i** and **2j**, were tested against EGFR, EGFR-L858R, and EGFR-L858R-T790 M enzymes. Derivatives with dichloro substitution on the phenyl ring exhibited the highest activity in the series. Specifically, compound **2i** (3,4-Cl) showed the strongest growth inhibitory effect with an IC_50_ value of 3.07 μM. Compound **2i** demonstrated significant selectivity against cancer cells compared to healthy cells (NIH/3T3). The lack of toxicity in healthy cells for derivatives such as **2f** (4-Cl) and **2h** (4-CN), with an IC_50_ > 1000 μM, indicates that this scaffold offers a safe profile. Derivative **2i** demonstrated superior inhibition with a very low IC_50_ value of 0.096 μM in both mutant enzyme types (L858R and L858R-T790M). Derivative **2j** (2,4-Cl) showed stable activity in all enzyme forms with an IC_50_ = 0.79 μM, demonstrating a profile resistant to mutations. When the obtained data are evaluated together, nonsmall cell lung cancer (NSCLC) continues to be a clinically significant public health problem. In NSCLC, EGFR tyrosine kinase inhibitors (EGFR-TKIs) are widely used, especially in cases carrying EGFR mutations, due to their targeted selectivity and more predictable safety profile compared to classical chemotherapeutic agents. However, acquired drug resistance frequently develops during treatment, particularly with first-generation EGFR-TKIs such as gefitinib and erlotinib. One of the most common acquired resistance mechanisms in clinical practice is the EGFR-T790 M mutation, which can emerge over time in tumors that initially carry an activation mutation (L858R). In these mutational subtypes, second-generation EGFR-TKIs (afatinib) and third-generation EGFR-TKIs (osimertinib) are preferred in clinical practice. The compounds obtained in this study contain bioisosteric and pharmacophoric motifs that have potential for anticancer activity and EGFR inhibition, by combining structural units such as a quinoline core, a halogen-substituted phenolic ring, and a hydrazone functional group.

## Supplementary Material



## Notes

^1^H NMR, ^13^C NMR, HRMS spectra of compounds **2a**–**2j**, IC_50_ graphics of compounds **2a**–**2j** against A549 cell line, 2D Docking poses of compounds **2a**–**2h** with EGFR, EGFR-L858R, EGFR-L858R-T790 M enzymes (PDF)
