# Supplementary material for: Design, Synthesis and Biological Effects Studies of Novel EGFR Inhibitors Targeting Wild-Type and Mutant EGFR (EGFR-L858R and EGFR-L858R/T790M)
Source: ACS Omega. 2026 Jun 18;11(25):36935–48. doi: 10.1021/acsomega.6c00728 (PMC13325101; doi:10.1021/acsomega.6c00728)
Supplement: Supplementary file 1 [file ao6c00728_si_001.pdf]

SUPPORTING INFORMATION

DESIGN, SYNTHESIS AND BIOLOGICAL EFFECTS STUDIES OF NOVEL EGFR INHIBITORS TARGETING WILD-TYPE AND MUTANT EGFR (EGFR-L858R and EGFR-L858R/T790M)

Derya Osmaniye[1,2], Ümit Balıkçı[3], Berkant Kurban[4,5], Yusuf Özkay[1,2*], Zafer Asım Kaplancıklı[1,6]

(1) Department of Pharmaceutical Chemistry, Faculty of Pharmacy, Anadolu University

(2) Central Analysis Laboratory, Faculty of Pharmacy, Anadolu University

(3) Faculty of Pharmacy, Anadolu University

(4) Institute of Graduate Education, Anadolu University

(5) Department of Pharmaceutical Chemistry, Faculty of Pharmacy, Afyonkarahisar Health Sciences University, Afyonkarahisar, Turkey

(6) *Department of Pharmacy Services, Vocational School of Health Services, Bilecik Seyh Edebali University*

* Correspondence: yozkay@anadolu.edu.tr

Tel: +90- *Fax*: +90-222 335 05 80-3778

Address: Anadolu University, Faculty of Pharmacy, Department of Pharmaceutical Chemistry, 26470, Eskişehir, Turkey.

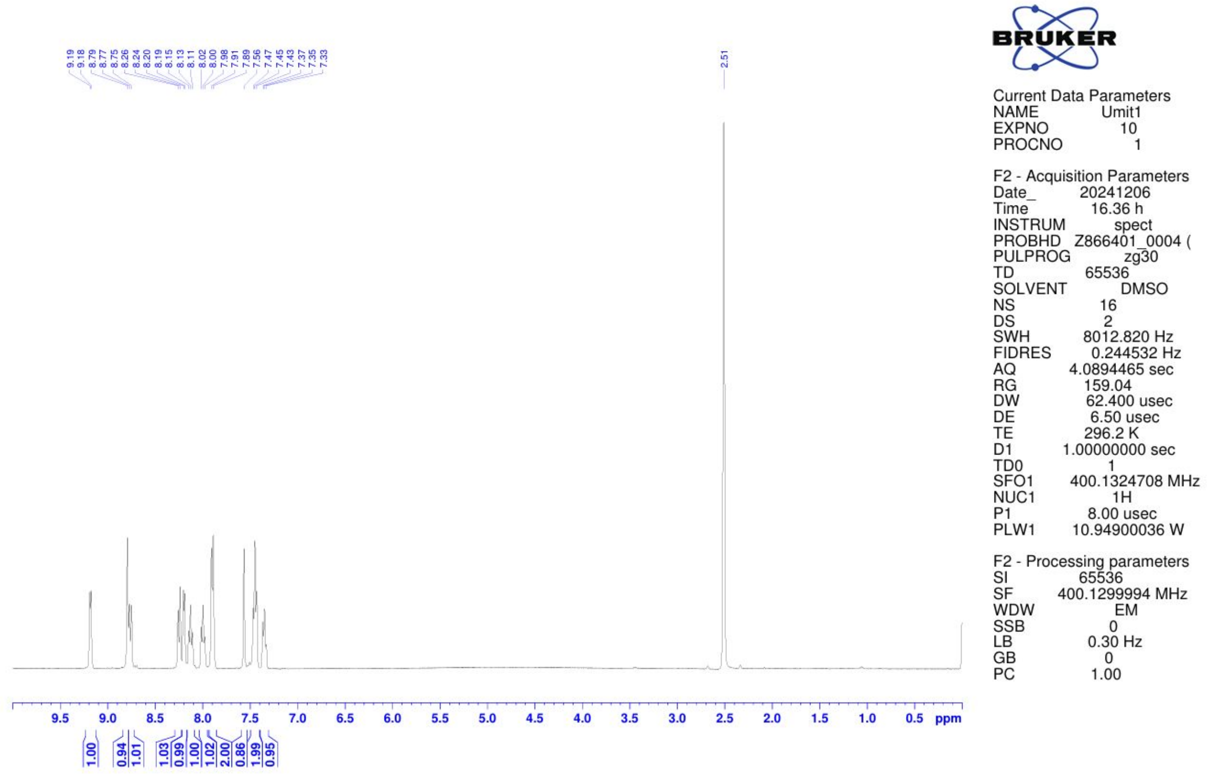

Figure S1. ^{1}H-NMR spectra of compound **2a**

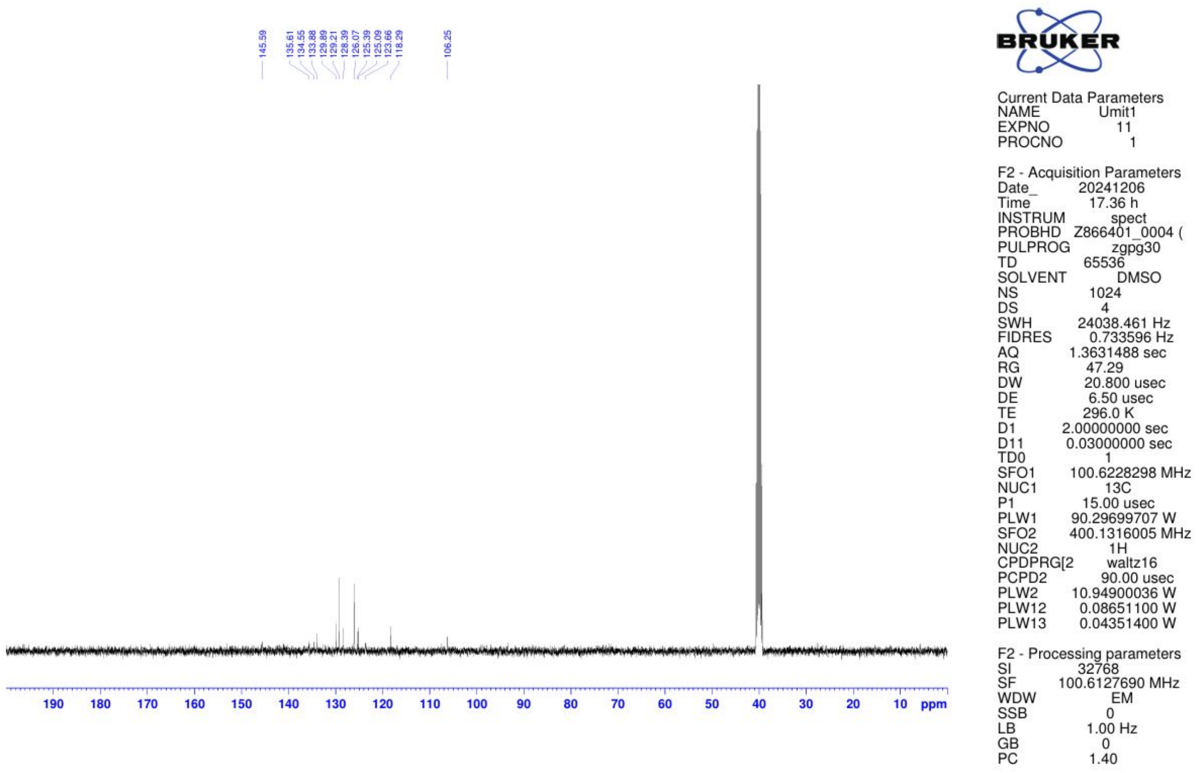

Figure S2. ^{13}C-NMR spectra of compound **2a**

Formula Predictor Report - Umit-1_40.lcd

Data File: C:\LabSolutions\Data\Analiz\Derya\Umit-1_40.lcd

Elmt	Val.	Min	Max	Elmt	Val.	Min	Max	Elmt	Val.	Min	Max	Elmt	Val.	Min	Max	Use Adduct
H	1	7	25	O	2	0	3	S	2	0	1	Ru	2	0	0	H
C	4	7	30	F	1	0	0	Cl	1	0	0	Pd	2	0	0	
N	3	0	5	P	3	0	0	Br	1	0	0	I	3	0	0	

Error Margin (ppm):	5	DBE Range:	0.0 - 20.0	Electron Ions:	both
HC Ratio:	unlimited	Apply N Rule:	yes	Use MSn Info:	yes
Max Isotopes:	5	Isotope RI (%):	1.00	Isotope Res:	9000
MSn Iso RI (%):	10.00	MSn Logic Mode:	AND	Max Results:	50

Event#: 1 MS(E+) Ret. Time : 1.573 Scan# : 237

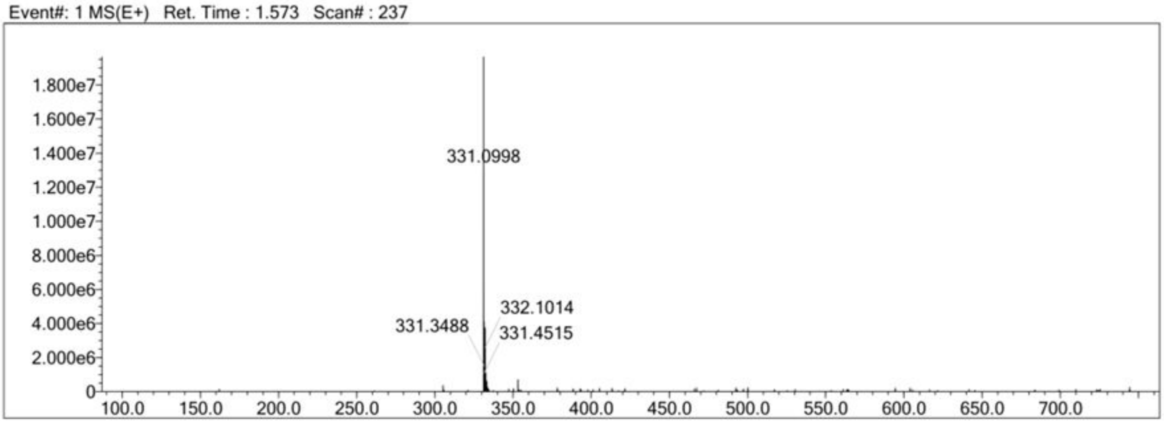

Measured region for 331.0998 m/z

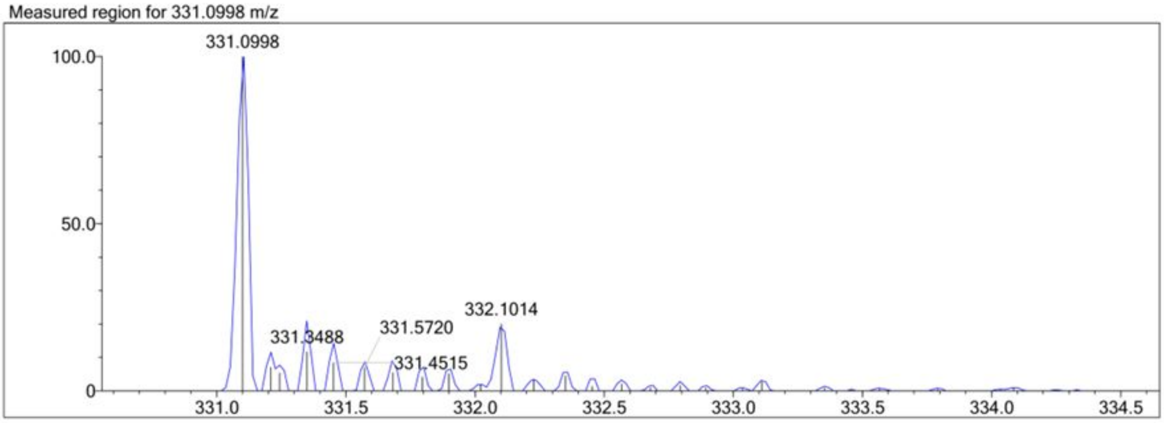

C19 H14 N4 S [M+H]+ : Predicted region for 331.1012 m/z

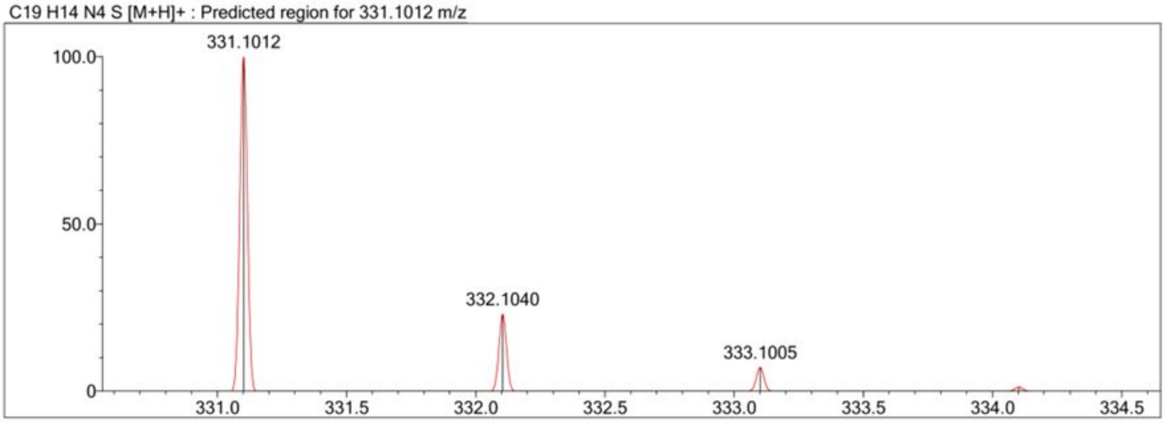

Rank	Score	Formula (M)	Ion	Meas. m/z	Pred. m/z	Df. (mDa)	Df. (ppm)	Iso	DBE
1	49.93	C19 H14 N4 S	[M+H]+	331.0998	331.1012	-1.4	-4.23	54.31	15.0

Figure S3. HRMS spectra of compound **2a**

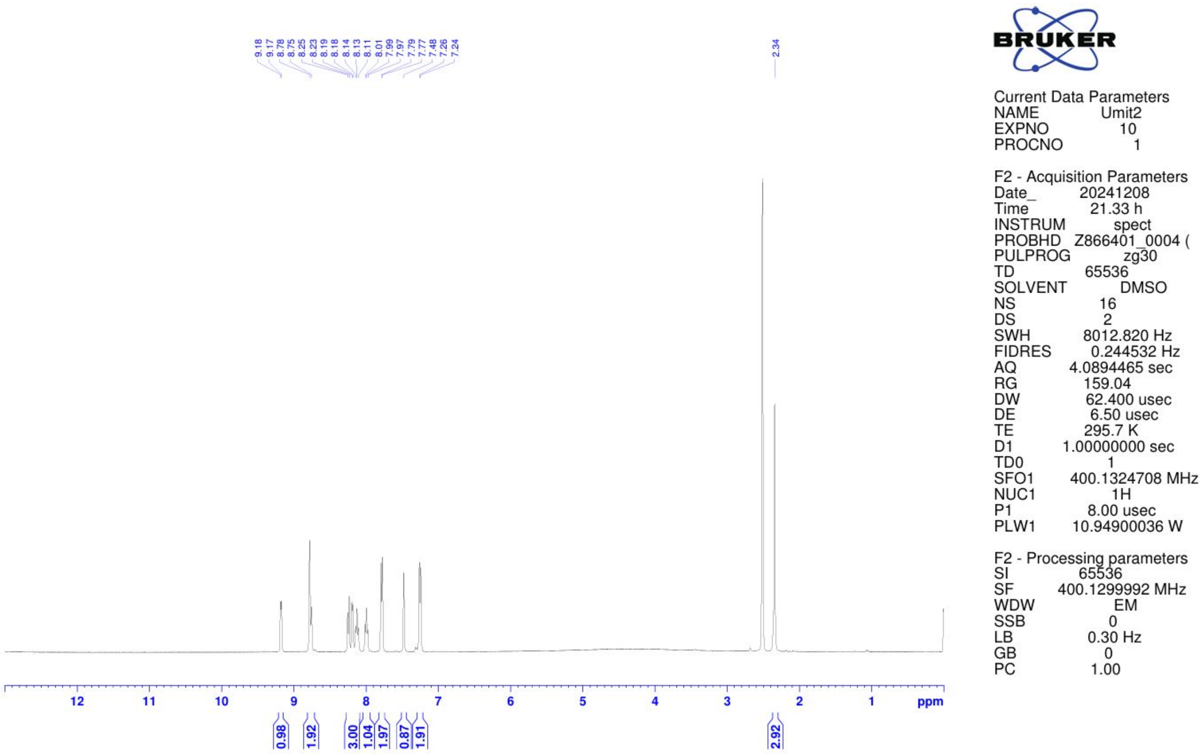

Figure S4. ^{1}H-NMR spectra of compound **2b**

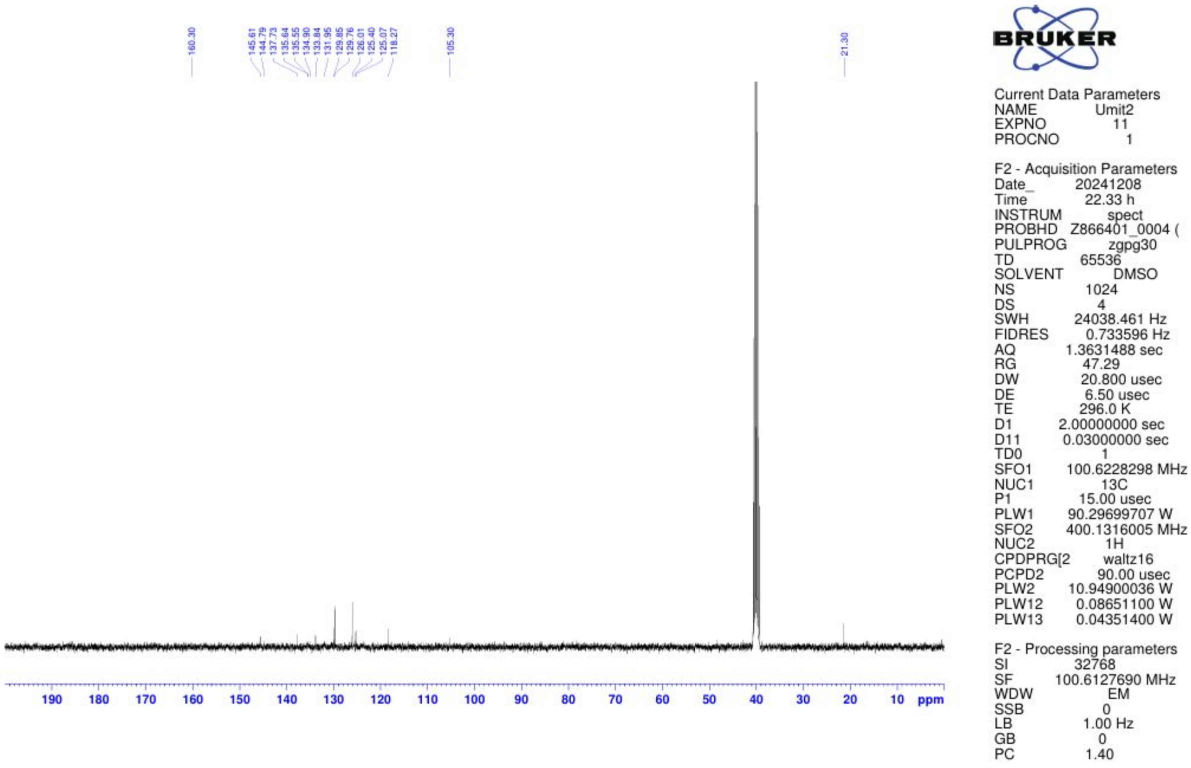

Figure S5. ^{13}C-NMR spectra of compound **2b**

Data File: C:\LabSolutions\Data\Analiz\Derya\Umit-2_41.lcd

Elmt	Val.	Min	Max	Elmt	Val.	Min	Max	Elmt	Val.	Min	Max	Elmt	Val.	Min	Max	Use Adduct
H	1	7	25	O	2	0	3	S	2	0	1	Ru	2	0	0	H
C	4	7	30	F	1	0	0	Cl	1	0	0	Pd	2	0	0	
N	3	0	5	P	3	0	0	Br	1	0	0	I	3	0	0	

Error Margin (ppm):	5	DBE Range:	0.0 - 20.0	Electron Ions:	both
HC Ratio:	unlimited	Apply N Rule:	yes	Use MSn Info:	yes
Max Isotopes:	5	Isotope RI (%):	1.00	Isotope Res:	9000
MSn Iso RI (%):	10.00	MSn Logic Mode:	AND	Max Results:	50

Event#: 1 MS(E+) Ret. Time : 1.773 Scan# : 267

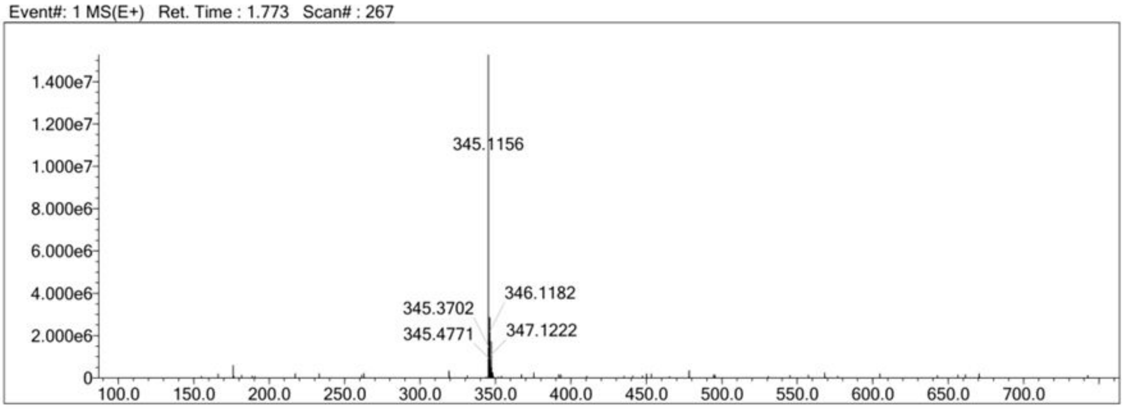

Measured region for 345.1156 m/z

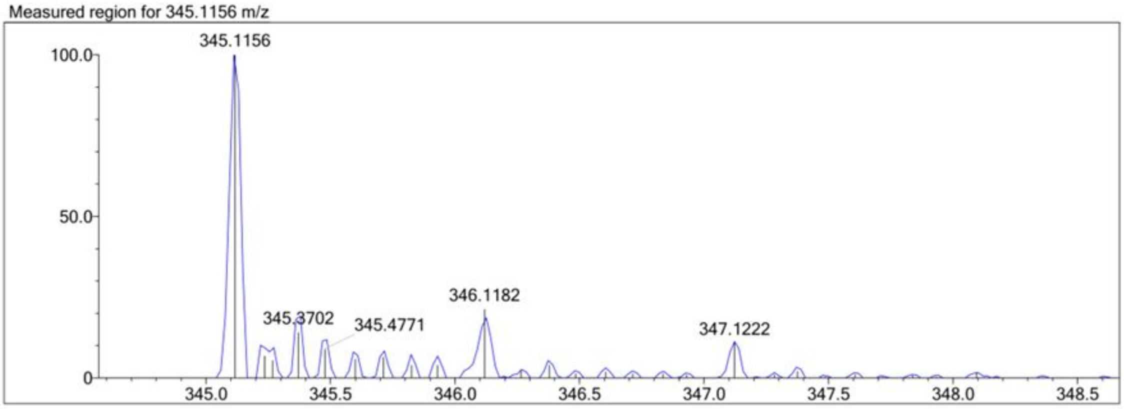

C20 H16 N4 S [M+H]+ : Predicted region for 345.1168 m/z

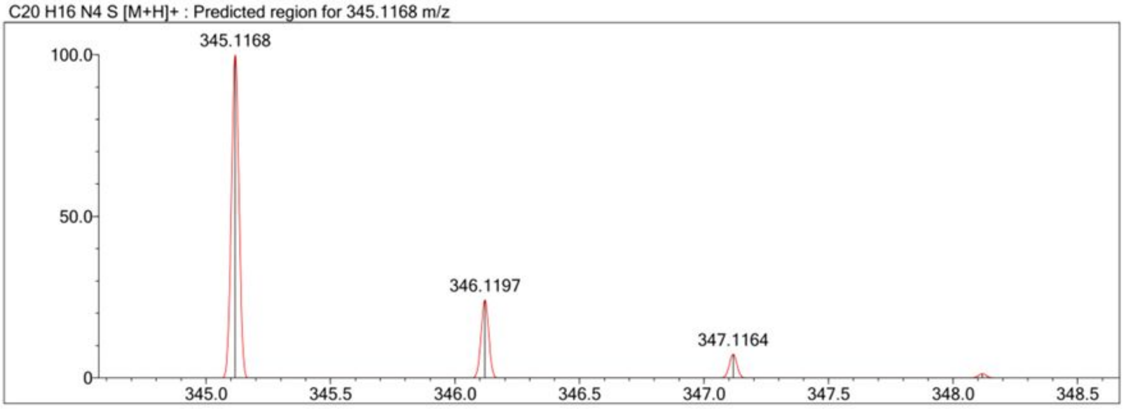

Rank	Score	Formula (M)	Ion	Meas. m/z	Pred. m/z	Df. (mDa)	Df. (ppm)	Iso	DBE
1	50.17	C20 H16 N4 S	[M+H]+	345.1156	345.1168	-1.2	-3.48	53.49	15.0

Figure S6. HRMS spectra of compound **2b**

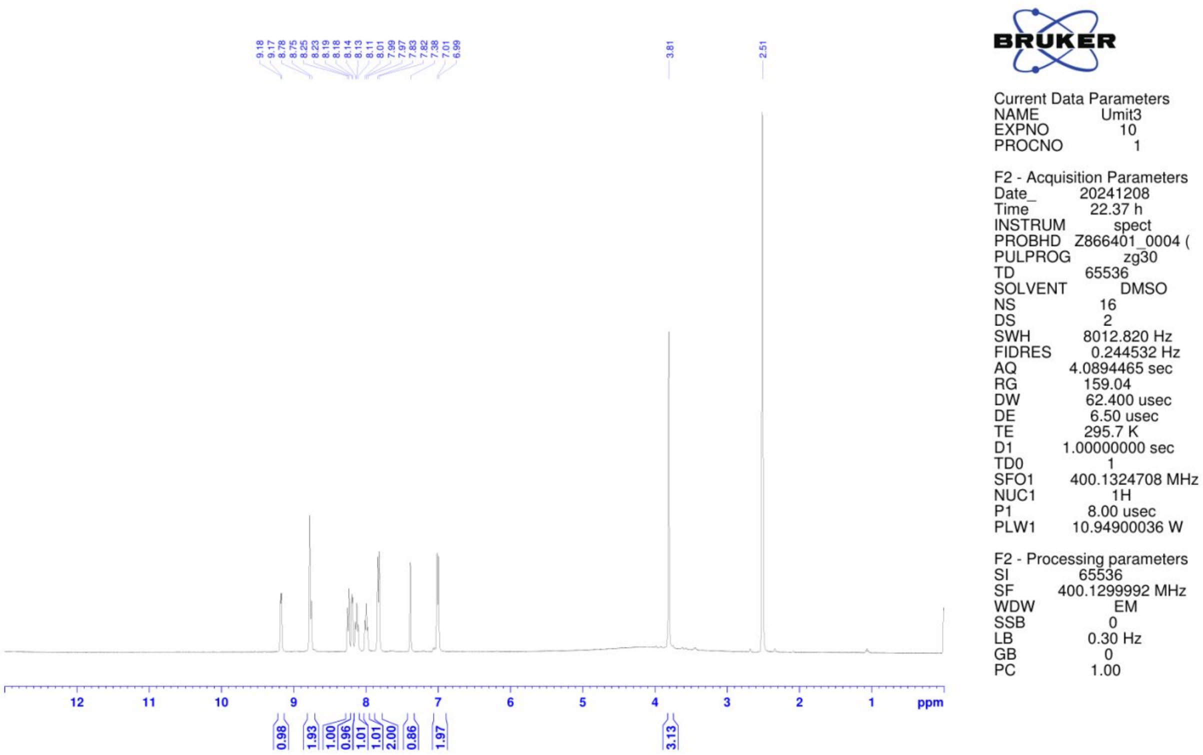

Figure S7. ^{1}H-NMR spectra of compound **2c**

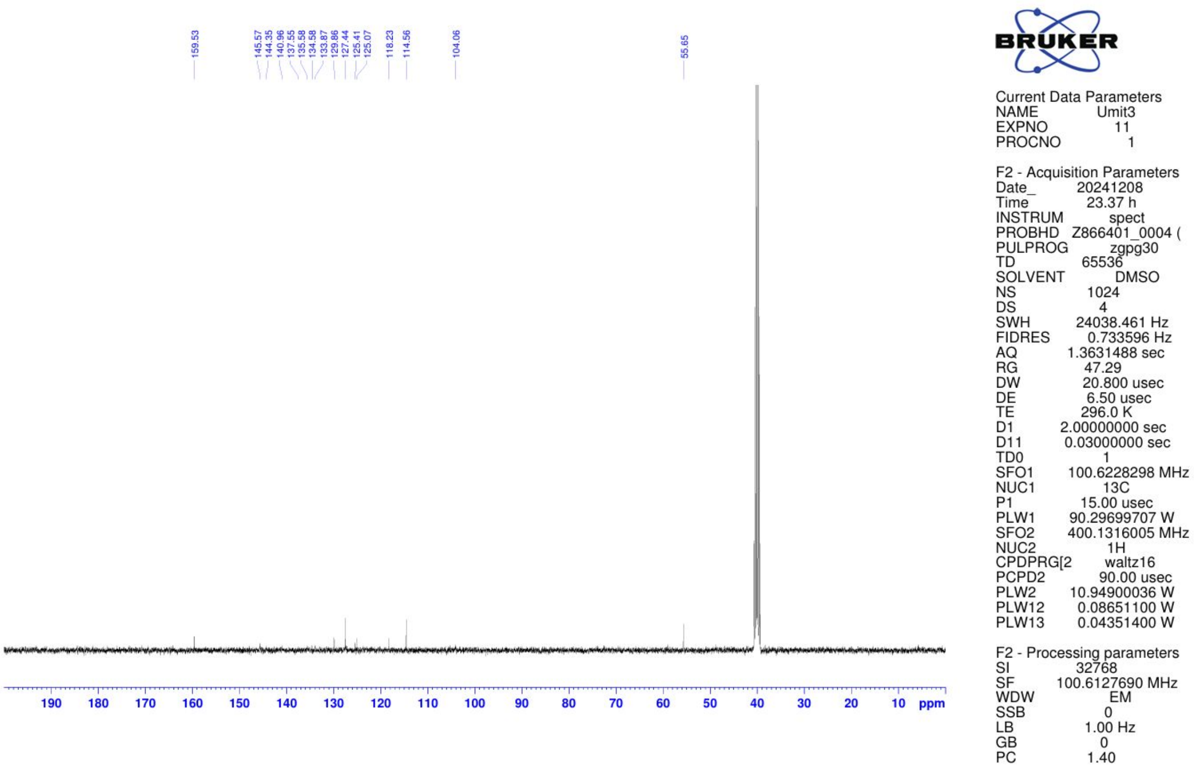

Figure S8. ^{13}C-NMR spectra of compound **2c**

Data File: C:\LabSolutions\Data\Analiz\Derya\Umit-3_42.lcd

Elmt	Val.	Min	Max	Elmt	Val.	Min	Max	Elmt	Val.	Min	Max	Elmt	Val.	Min	Max	Use Adduct
H	1	7	25	O	2	0	3	S	2	0	1	Ru	2	0	0	H
C	4	7	30	F	1	0	0	Cl	1	0	0	Pd	2	0	0	
N	3	0	5	P	3	0	0	Br	1	0	0	I	3	0	0	

Error Margin (ppm):	5	DBE Range:	0.0 - 19.0	Electron Ions:	both
HC Ratio:	unlimited	Apply N Rule:	yes	Use MSn Info:	yes
Max Isotopes:	5	Isotope RI (%):	1.00	Isotope Res:	9000
MSn Iso RI (%):	10.00	MSn Logic Mode:	AND	Max Results:	50

Event#: 1 MS(E+) Ret. Time : 1.453 Scan# : 219

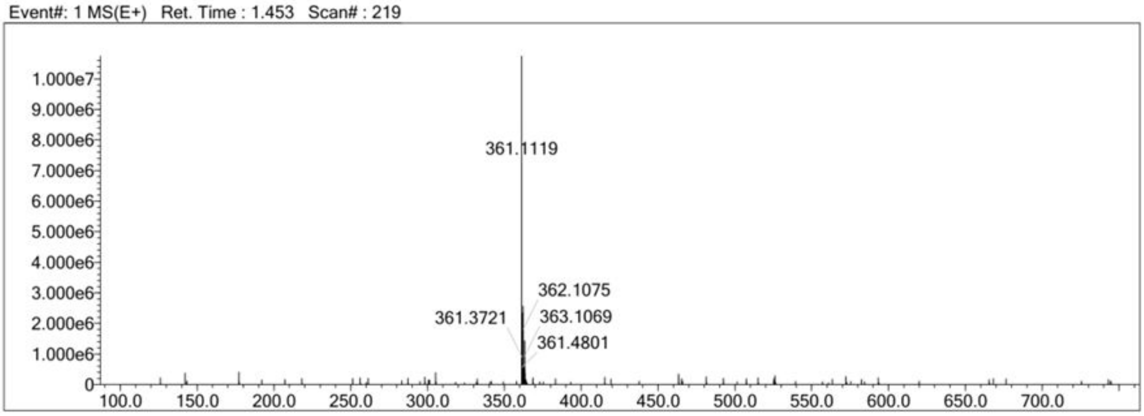

Measured region for 361.1119 m/z

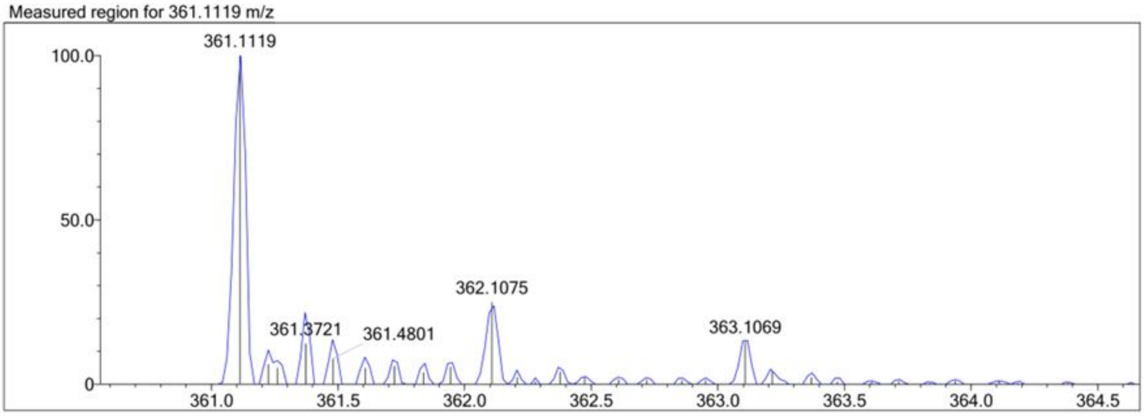

C20 H16 N4 O S [M+H]+ : Predicted region for 361.1118 m/z

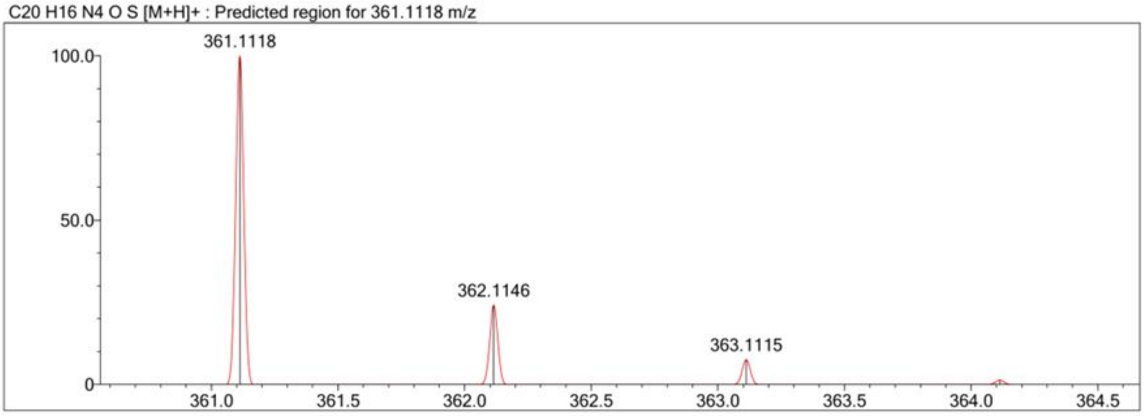

Rank	Score	Formula (M)	Ion	Meas. m/z	Pred. m/z	Df. (mDa)	Df. (ppm)	Iso	DBE
1	76.35	C20 H16 N4 O S	[M+H]+	361.1119	361.1118	0.1	0.28	76.35	15.0

Figure S9. HRMS spectra of compound **2c**

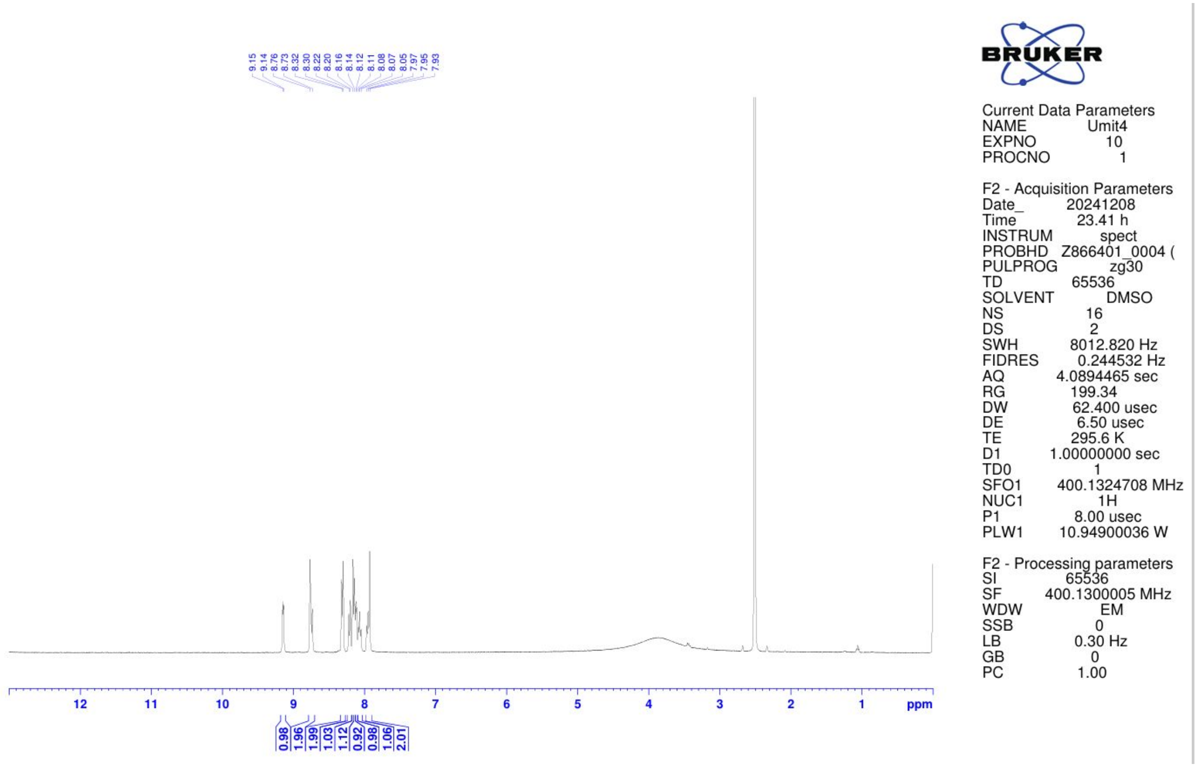

Figure S10. ^{1}H-NMR spectra of compound **2d**

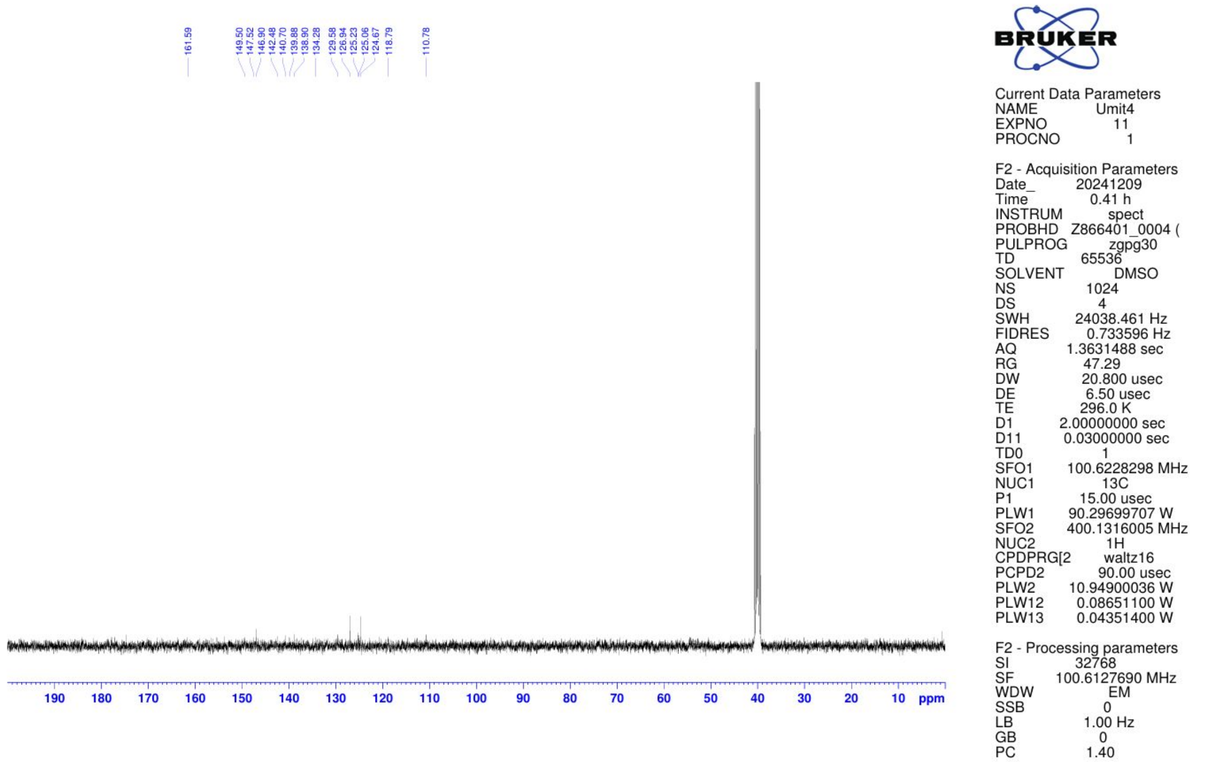

Figure S11. ^{13}C-NMR spectra of compound **2d**

Data File: C:\LabSolutions\Data\Analiz\Derya\Umit-4_43.lcd

Elmt	Val.	Min	Max	Elmt	Val.	Min	Max	Elmt	Val.	Min	Max	Elmt	Val.	Min	Max	Use Adduct
H	1	7	25	O	2	0	3	S	2	0	1	Ru	2	0	0	H
C	4	7	30	F	1	0	0	Cl	1	0	0	Pd	2	0	0	
N	3	0	5	P	3	0	0	Br	1	0	0	I	3	0	0	

Error Margin (ppm): 5
HC Ratio: unlimited
Max Isotopes: 5
MSn Iso RI (%): 10.00

DBE Range: 0.0 - 19.0
Apply N Rule: yes
Isotope RI (%): 1.00
MSn Logic Mode: AND

Electron Ions: both
Use MSn Info: yes
Isotope Res: 9000
Max Results: 50

Event#: 1 MS(E+) Ret. Time : 1.520 -> 1.627 Scan# : 229 -> 245

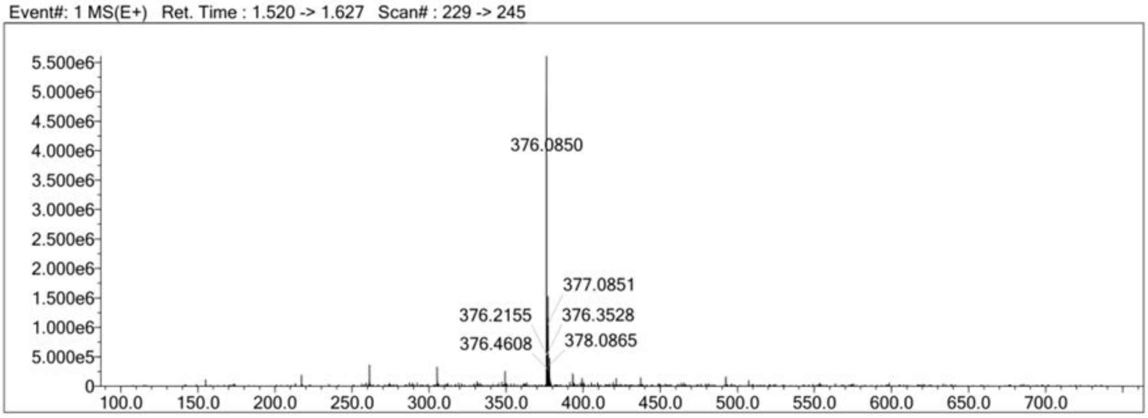

Measured region for 376.0850 m/z

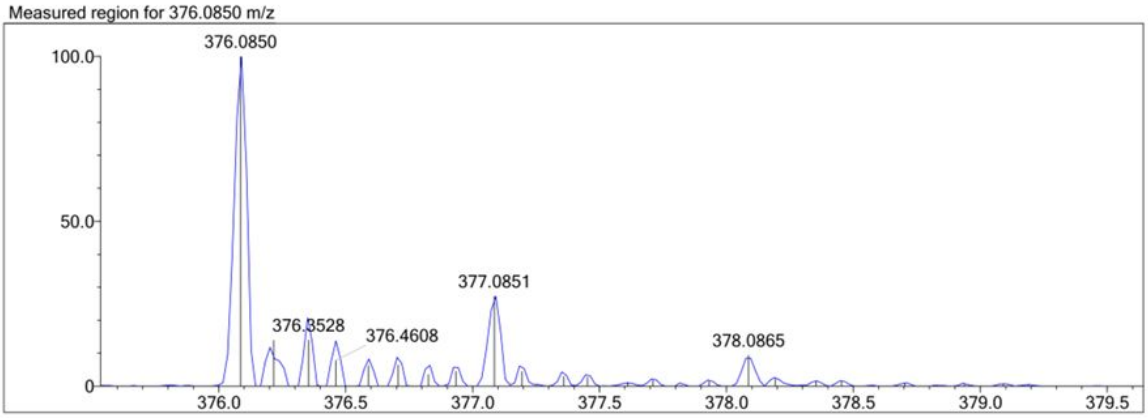

C19 H13 N5 O2 S [M+H]+ : Predicted region for 376.0863 m/z

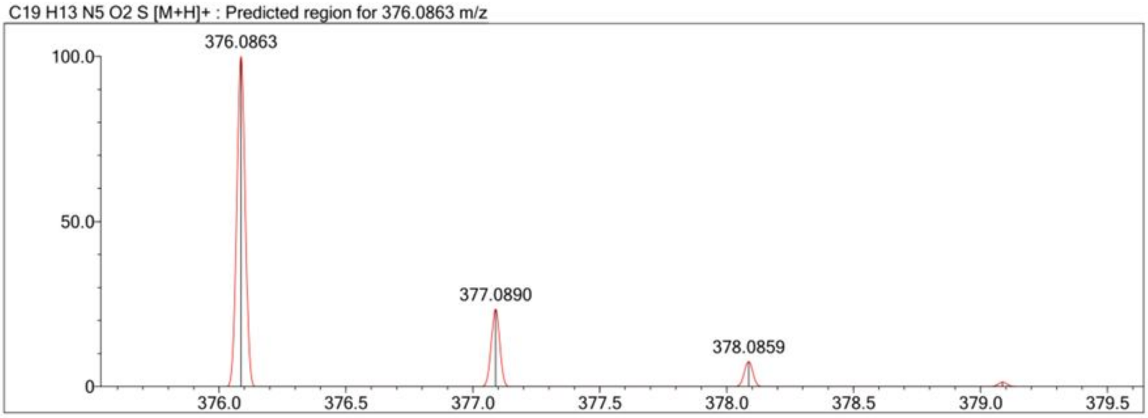

Rank	Score	Formula (M)	Ion	Meas. m/z	Pred. m/z	Df. (mDa)	Df. (ppm)	Iso	DBE
1	83.36	C19 H13 N5 O2 S	[M+H]+	376.0850	376.0863	-1.3	-3.46	88.82	16.0

Figure S12. HRMS spectra of compound **2d**

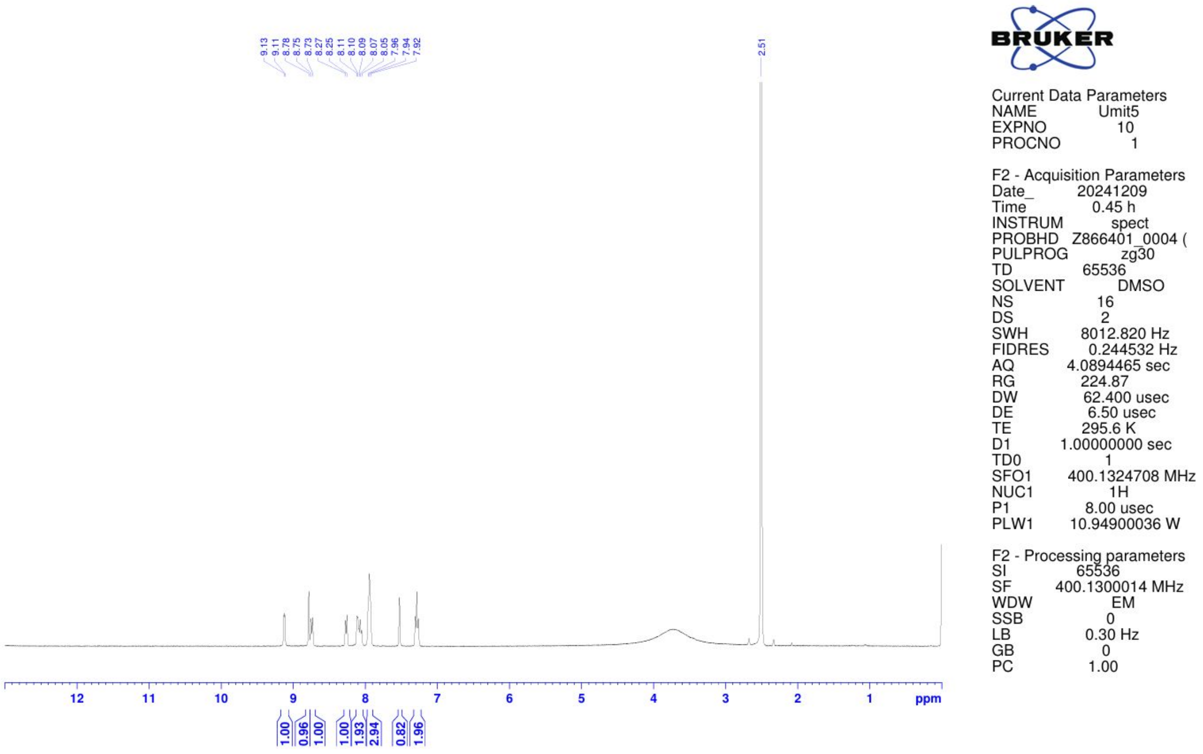

Figure S13. ^{1}H-NMR spectra of compound **2e**

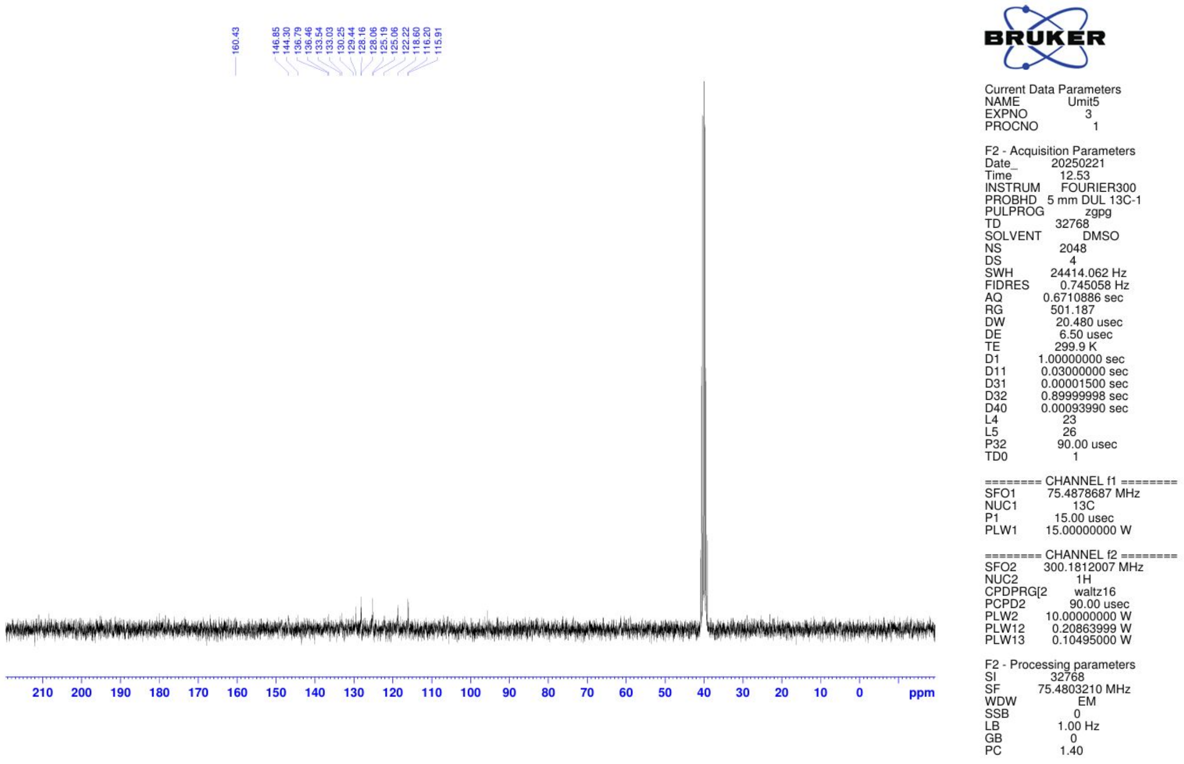

Figure S14. ^{13}C-NMR spectra of compound **2e**

Data File: C:\LabSolutions\Data\Analiz\Derya\Umit-5_44.lcd

Elmt	Val.	Min	Max	Elmt	Val.	Min	Max	Elmt	Val.	Min	Max	Elmt	Val.	Min	Max	Use Adduct
H	1	7	25	O	2	0	3	S	2	0	1	Ru	2	0	0	H
C	4	7	30	F	1	0	1	Cl	1	0	0	Pd	2	0	0	
N	3	2	5	P	3	0	0	Br	1	0	0	I	3	0	0	

Error Margin (ppm): 5
HC Ratio: unlimited
Max Isotopes: 5
MSn Iso RI (%): 10.00

DBE Range: 0.0 - 19.0
Apply N Rule: yes
Isotope RI (%): 1.00
MSn Logic Mode: AND

Electron Ions: both
Use MSn Info: yes
Isotope Res: 9000
Max Results: 50

Event#: 1 MS(E+) Ret. Time : 1.613 Scan# : 243

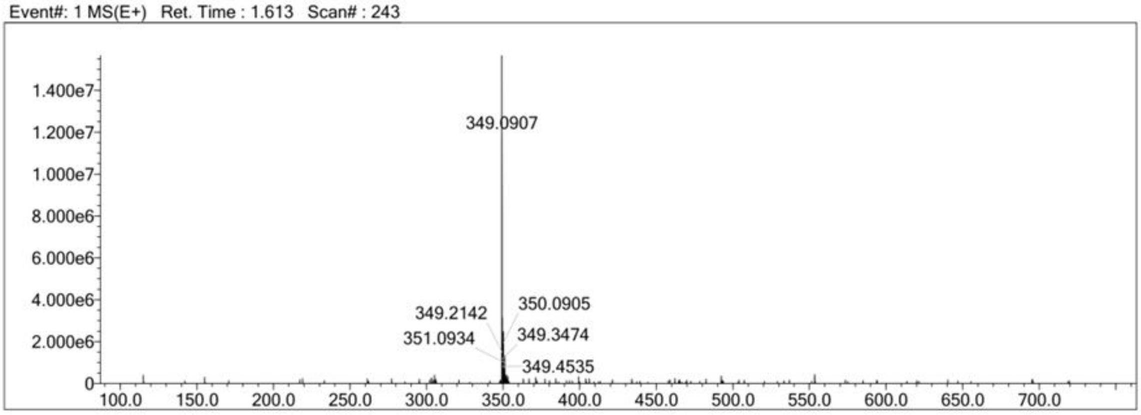

Measured region for 349.0907 m/z

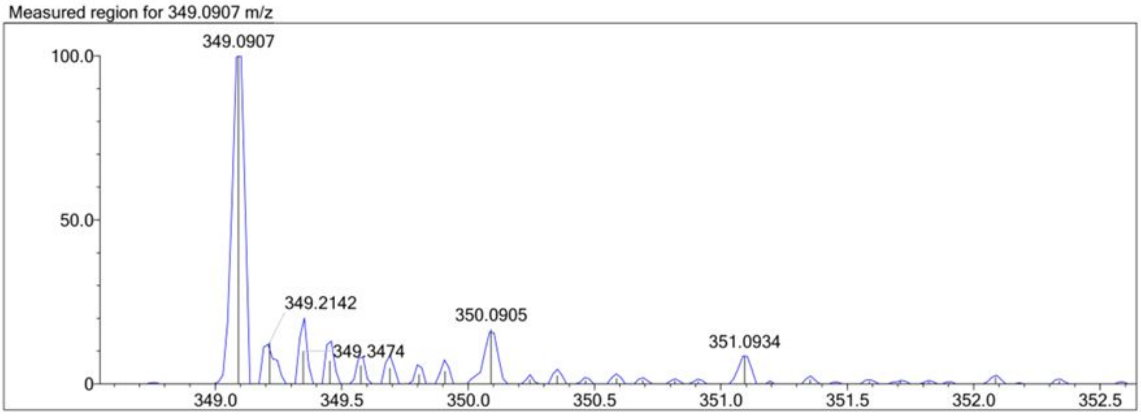

C19 H13 N4 F S [M+H]+ : Predicted region for 349.0918 m/z

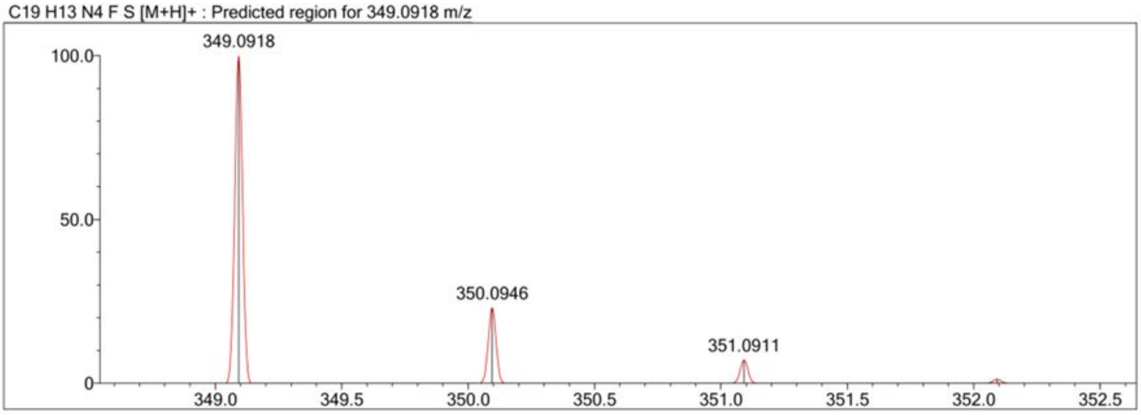

Rank	Score	Formula (M)	Ion	Meas. m/z	Pred. m/z	Df. (mDa)	Df. (ppm)	Iso	DBE
1	65.10	C19 H13 N4 F S	[M+H]+	349.0907	349.0918	-1.1	-3.15	68.79	15.0

Figure S15. HRMS spectra of compound **2e**

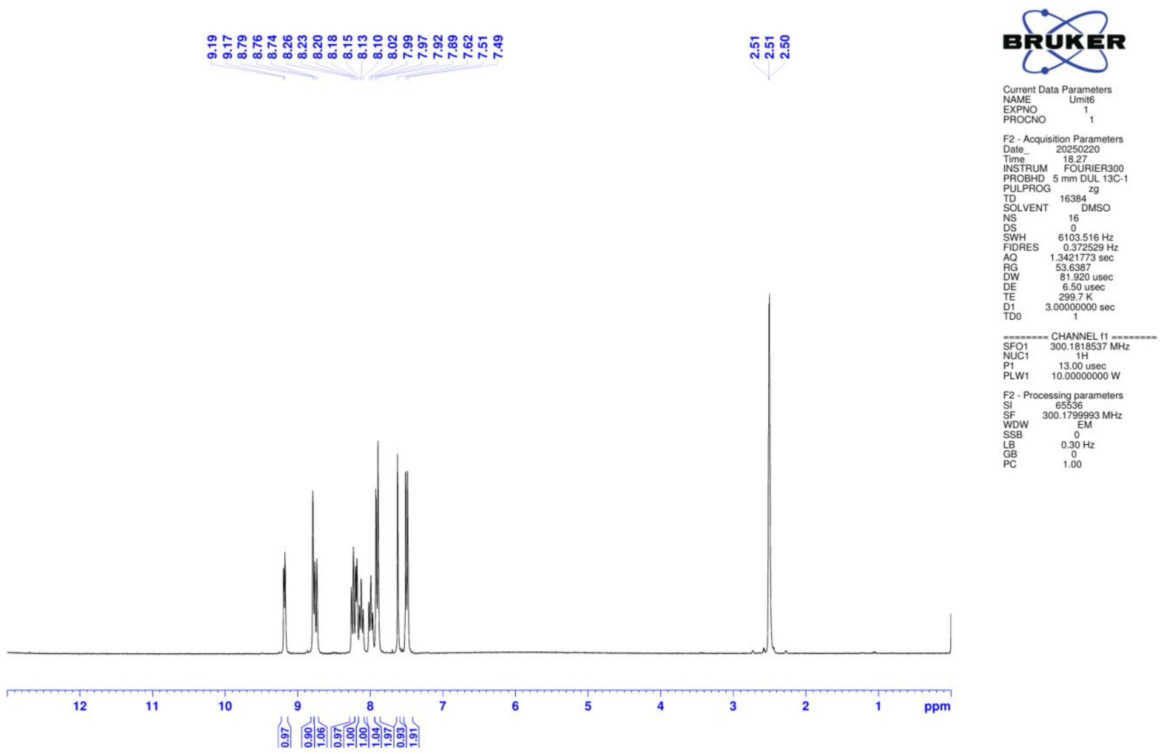

Figure S16. ^{1}H-NMR spectra of compound **2f**

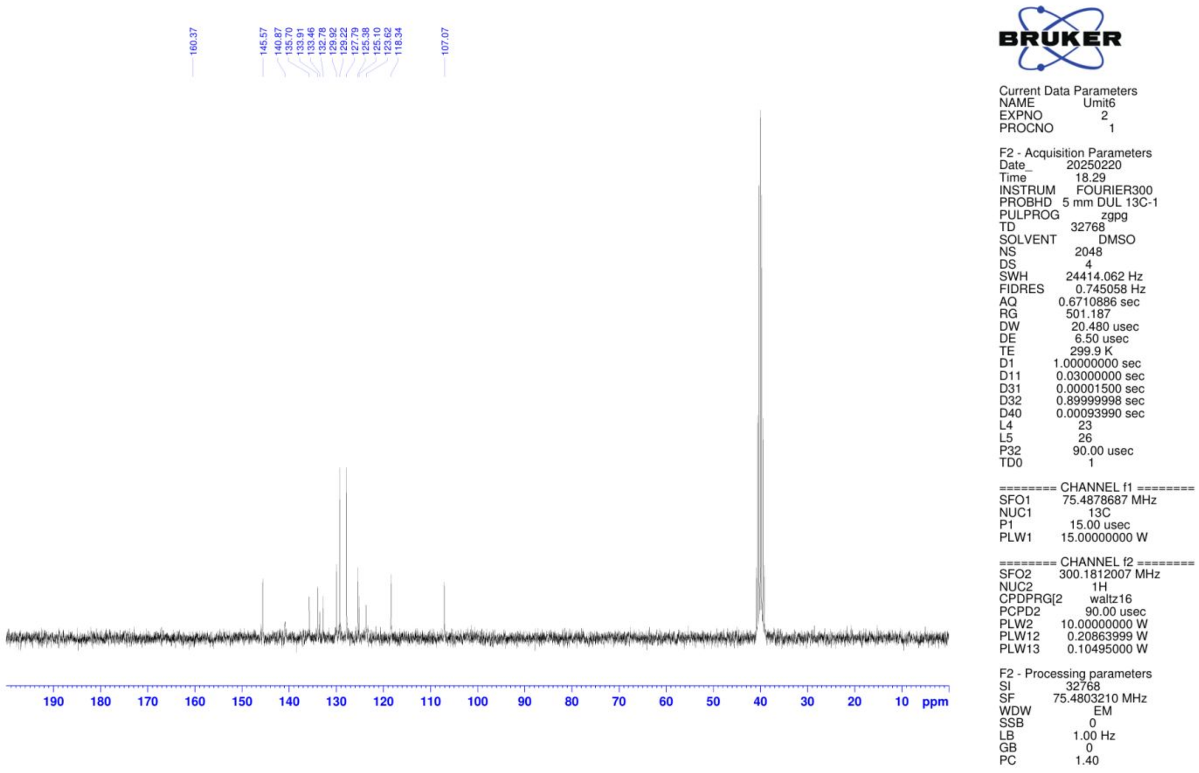

Figure S17. ^{13}C-NMR spectra of compound **2f**

Data File: C:\LabSolutions\Data\Analiz\Derya\Umit-7_46.lcd

Elmt	Val.	Min	Max	Elmt	Val.	Min	Max	Elmt	Val.	Min	Max	Elmt	Val.	Min	Max	Use Adduct
H	1	7	25	O	2	0	3	S	2	0	1	Ru	2	0	0	H
C	4	7	30	F	1	0	1	Cl	1	0	1	Pd	2	0	0	
N	3	2	5	P	3	0	0	Br	1	0	0	I	3	0	0	

Error Margin (ppm): 5
HC Ratio: unlimited
Max Isotopes: 5
MSn Iso RI (%): 10.00

DBE Range: 12.0 - 19.0
Apply N Rule: yes
Isotope RI (%): 1.00
MSn Logic Mode: AND

Electron Ions: both
Use MSn Info: yes
Isotope Res: 9000
Max Results: 50

Event#: 1 MS(E+) Ret. Time : 1.840 Scan# : 277

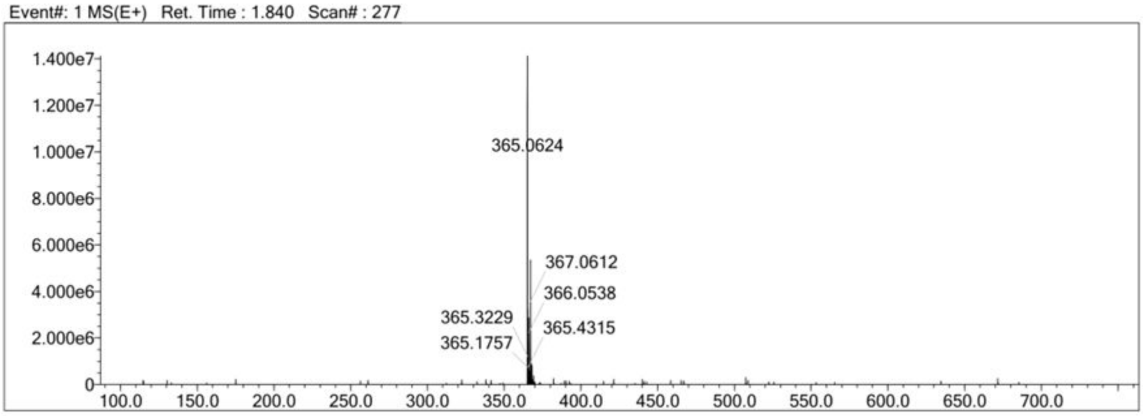

Measured region for 365.0624 m/z

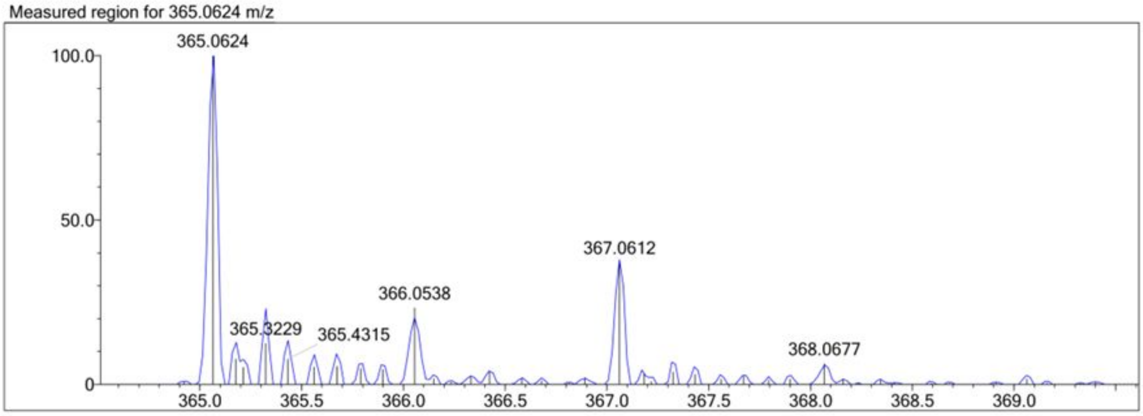

C19 H13 N4 S Cl [M+H]+ : Predicted region for 365.0622 m/z

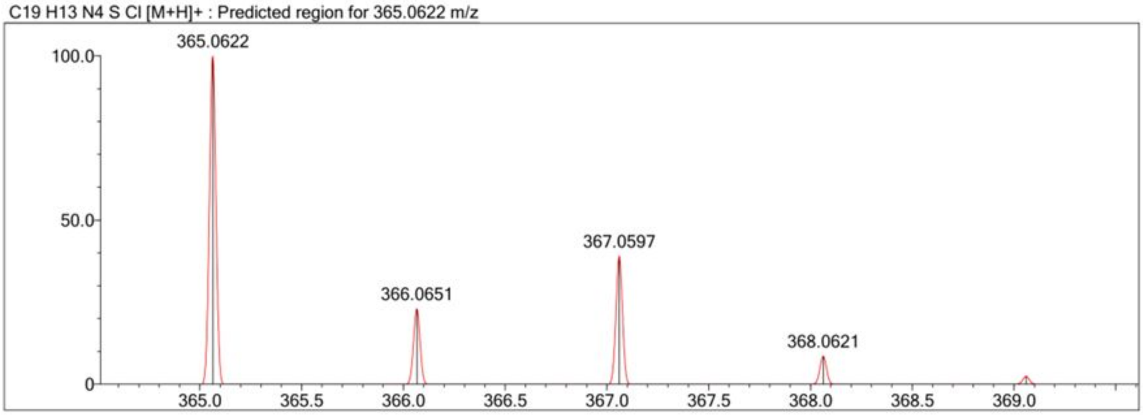

Rank	Score	Formula (M)	Ion	Meas. m/z	Pred. m/z	Df. (mDa)	Df. (ppm)	Iso	DBE
1	74.03	C19 H13 N4 S Cl	[M+H]+	365.0624	365.0622	0.2	0.55	74.03	15.0

Figure S18. HRMS spectra of compound **2f**

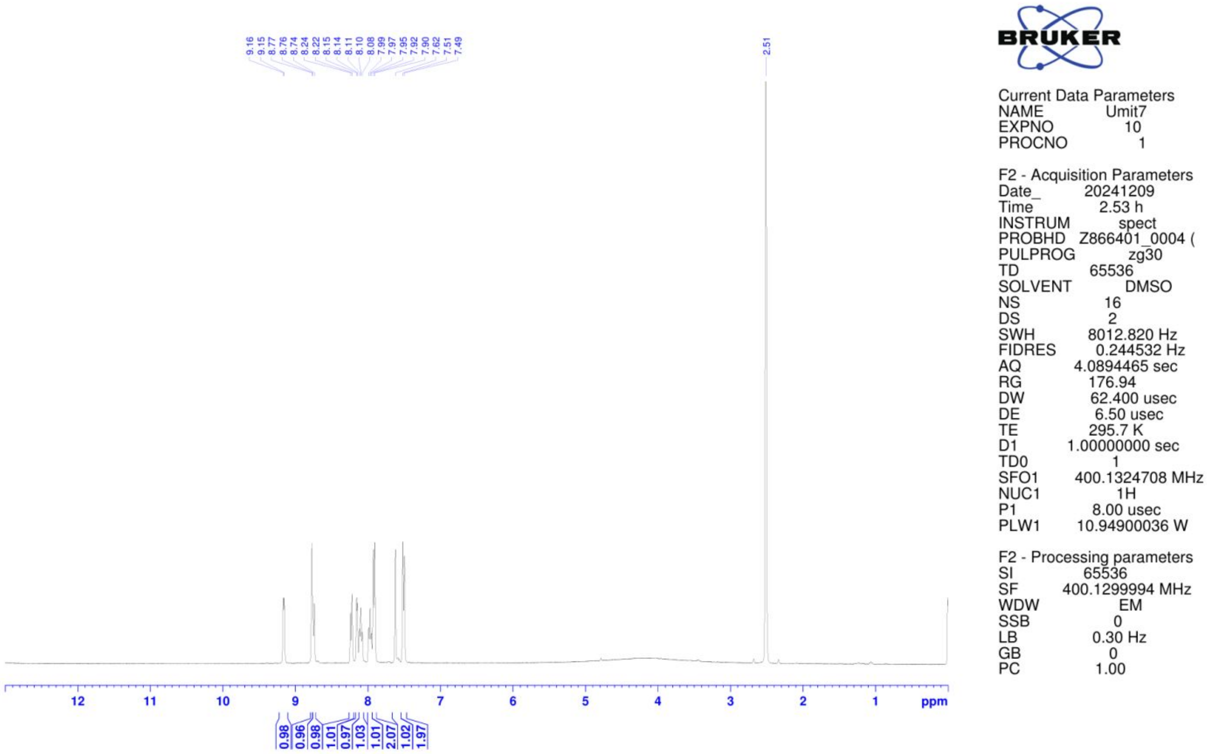

Figure S19. ^{1}H-NMR spectra of compound **2g**

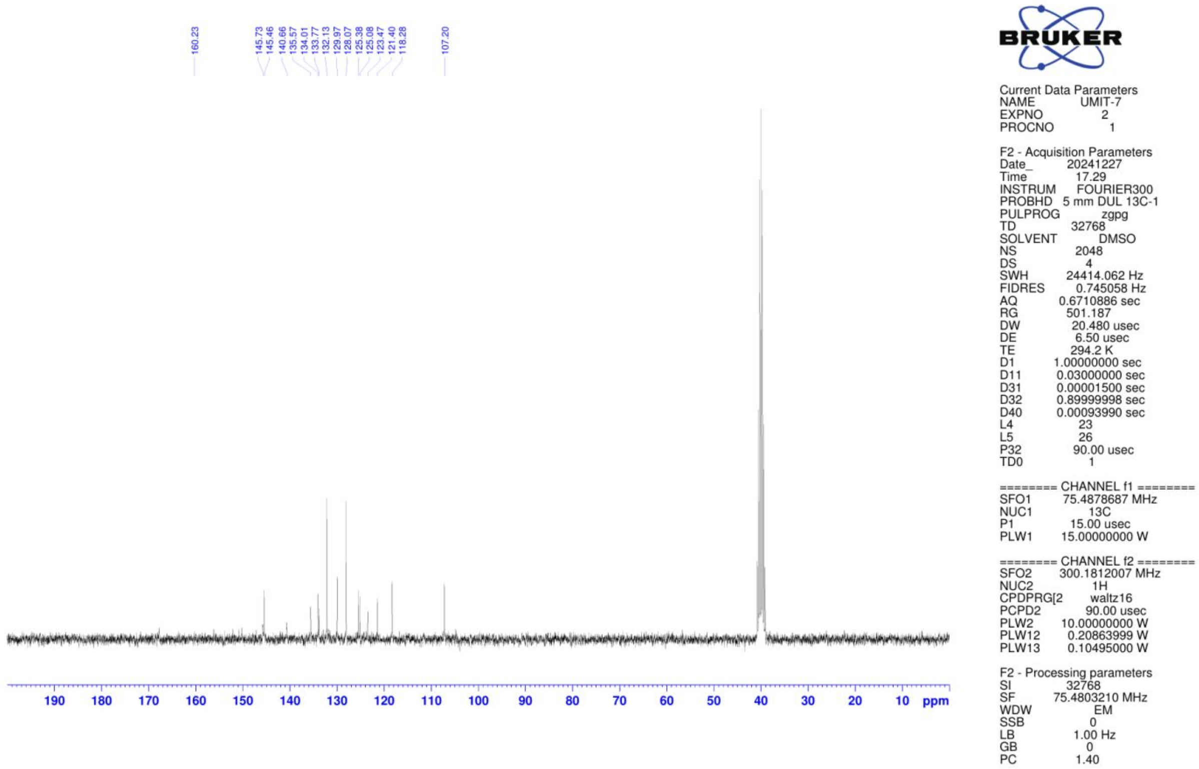

Figure S20. ^{13}C-NMR spectra of compound **2g**

Data File: C:\LabSolutions\Data\Analiz\Derya\Umit-7_1.lcd

Elmt	Val.	Min	Max	Elmt	Val.	Min	Max	Elmt	Val.	Min	Max	Elmt	Val.	Min	Max	Use Adduct
H	1	10	20	O	2	0	3	S	2	0	2	Ru	2	0	0	H
C	4	17	19	F	1	0	0	Cl	1	0	0	Pd	2	0	0	
N	3	0	7	P	3	0	0	Br	1	0	1	I	3	0	0	

Error Margin (ppm):	5	DBE Range:	5.0 - 25.0	Electron Ions:	both
HC Ratio:	unlimited	Apply N Rule:	yes	Use MSn Info:	yes
Max Isotopes:	5	Isotope RI (%):	1.00	Isotope Res:	9000
MSn Iso RI (%):	10.00	MSn Logic Mode:	AND	Max Results:	50

Event#: 1 MS(E+) Ret. Time : 1.867 -> 1.947 Scan# : 281 -> 293

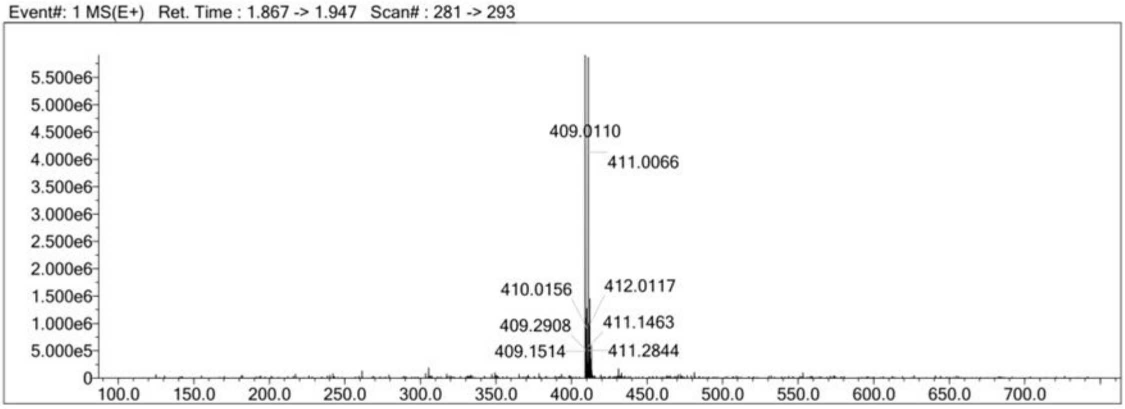

Measured region for 409.0110 m/z

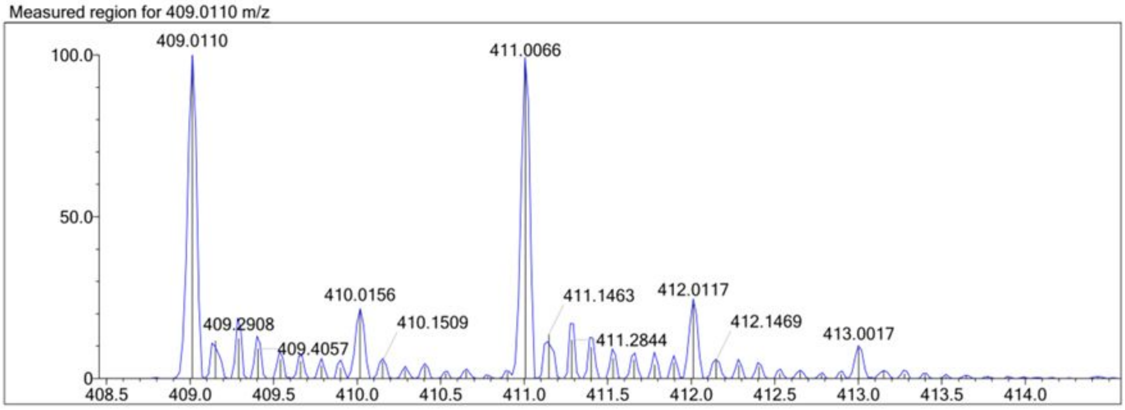

C19 H13 N4 S Br [M+H]+ : Predicted region for 409.0117 m/z

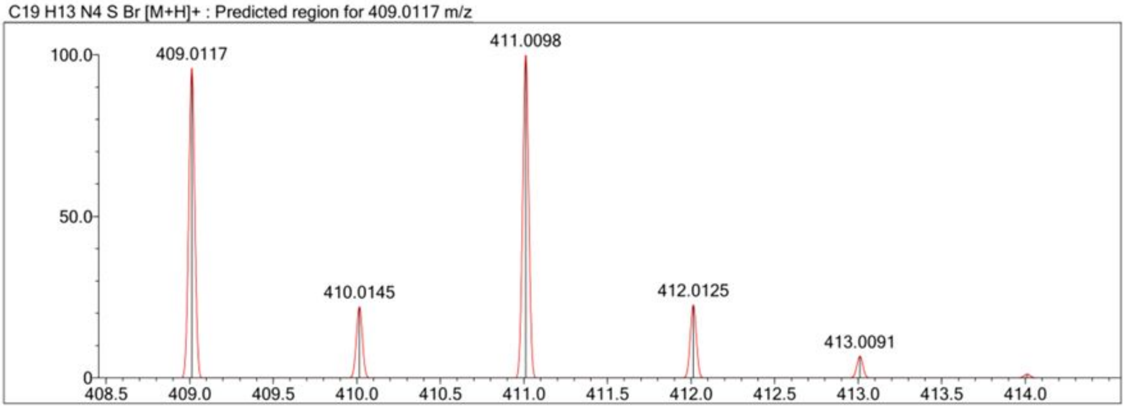

Rank	Score	Formula (M)	Ion	Meas. m/z	Pred. m/z	Df. (mDa)	Df. (ppm)	Iso	DBE
1	95.29	C19 H13 N4 S Br	[M+H]+	409.0110	409.0117	-0.7	-1.71	97.02	15.0

Figure S21. HRMS spectra of compound **2g**

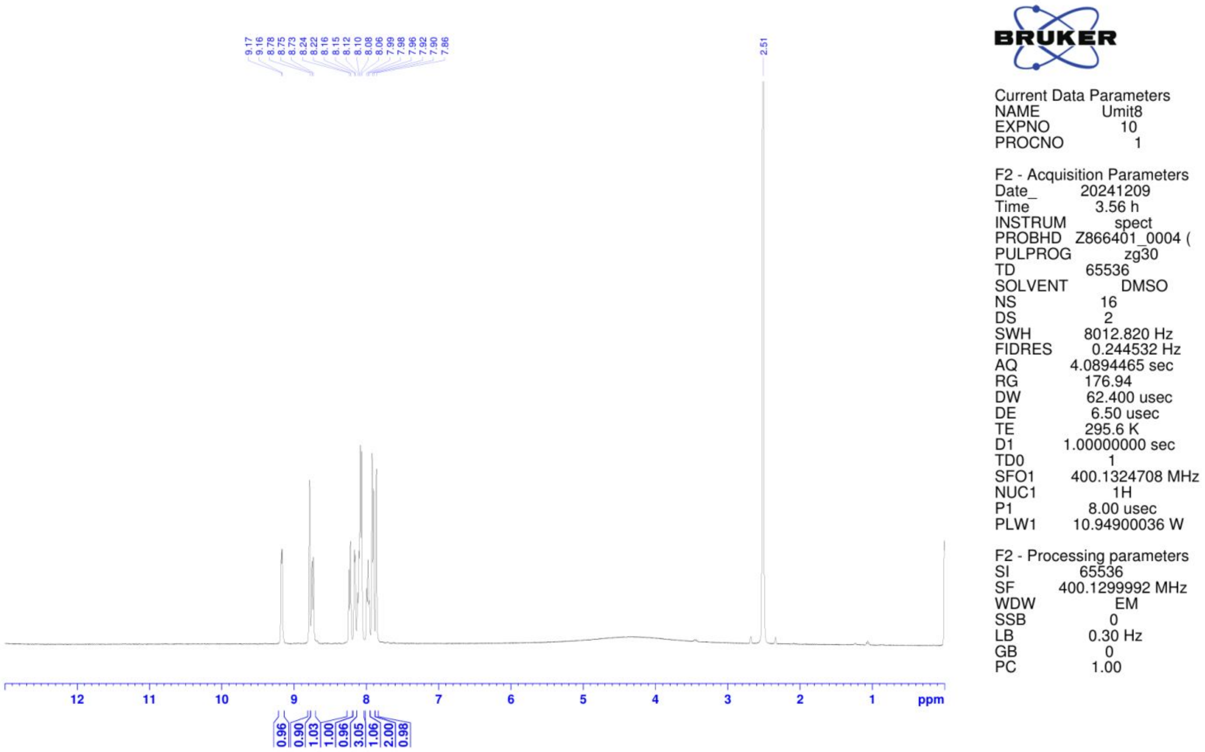

Figure S22. ^{1}H-NMR spectra of compound **2h**

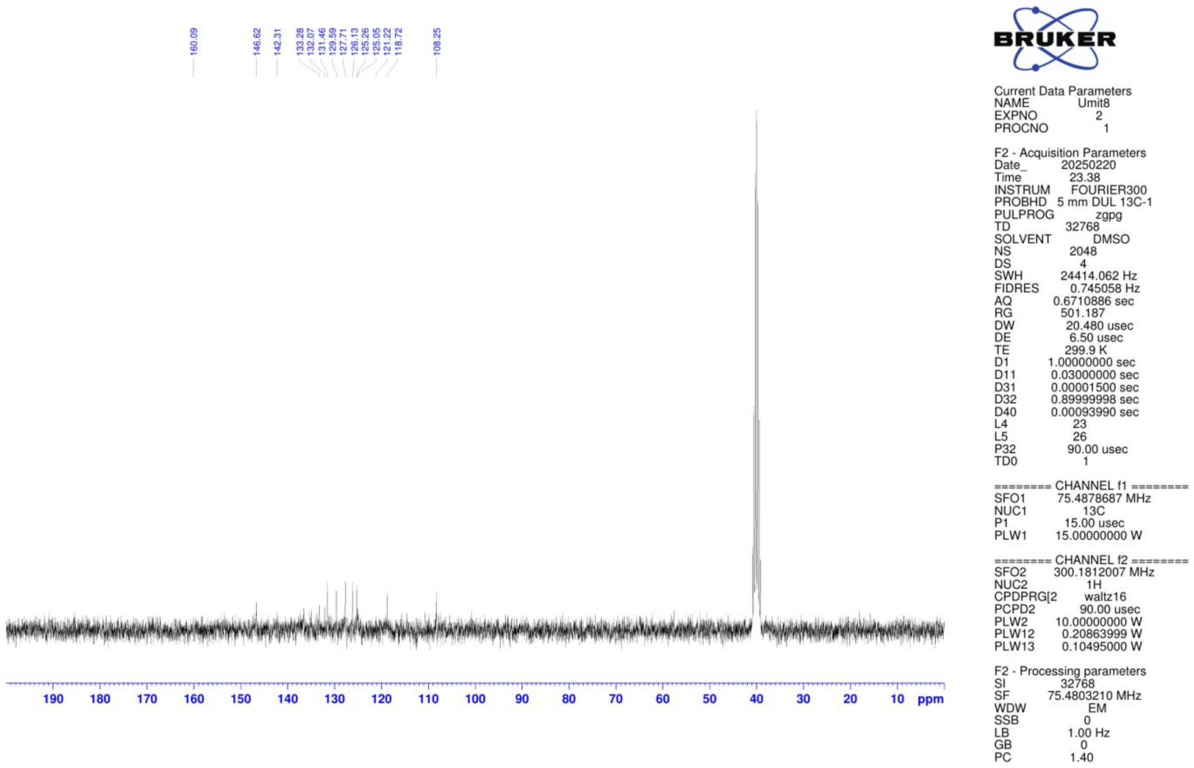

Figure S23. ^{13}C-NMR spectra of compound **2h**

Data File: C:\LabSolutions\Data\Analiz\Derya\Umit-8_47.lcd

Elmt	Val.	Min	Max	Elmt	Val.	Min	Max	Elmt	Val.	Min	Max	Elmt	Val.	Min	Max	Use Adduct
H	1	7	25	O	2	0	3	S	2	0	1	Ru	2	0	0	H
C	4	7	30	F	1	0	0	Cl	1	0	0	Pd	2	0	0	
N	3	2	5	P	3	0	0	Br	1	0	0	I	3	0	0	

Error Margin (ppm):	5	DBE Range:	12.0 - 19.0	Electron Ions:	both
HC Ratio:	unlimited	Apply N Rule:	yes	Use MSn Info:	yes
Max Isotopes:	5	Isotope RI (%):	1.00	Isotope Res:	9000
MSn Iso RI (%):	10.00	MSn Logic Mode:	AND	Max Results:	50

Event#: 1 MS(E+) Ret. Time : 1.480 Scan# : 223

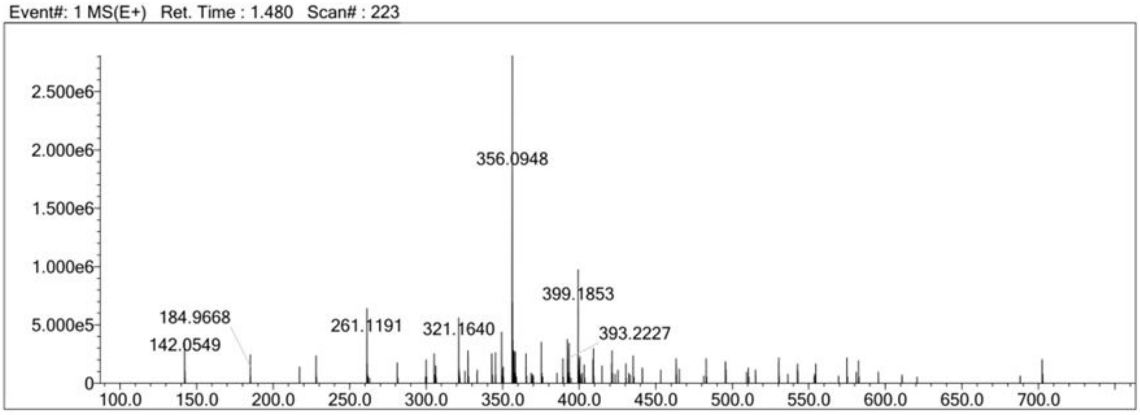

Measured region for 356.0948 m/z

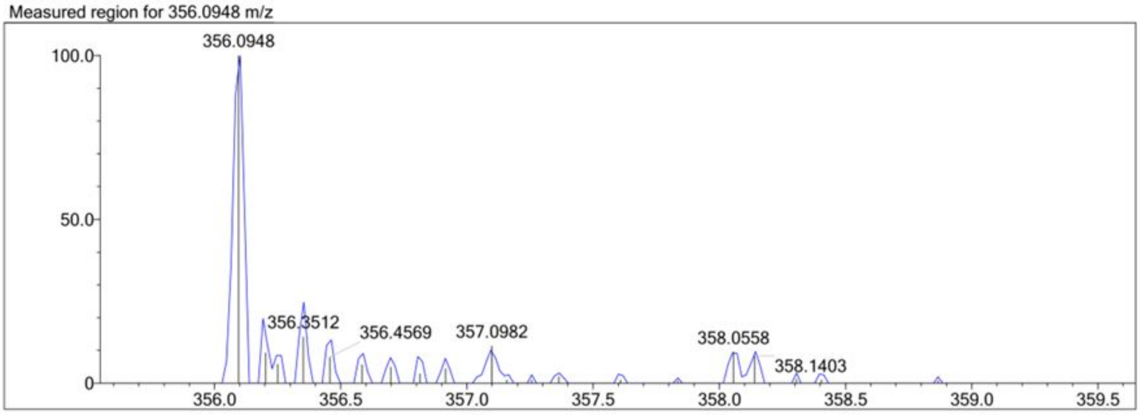

C20 H13 N5 S [M+H]+ : Predicted region for 356.0964 m/z

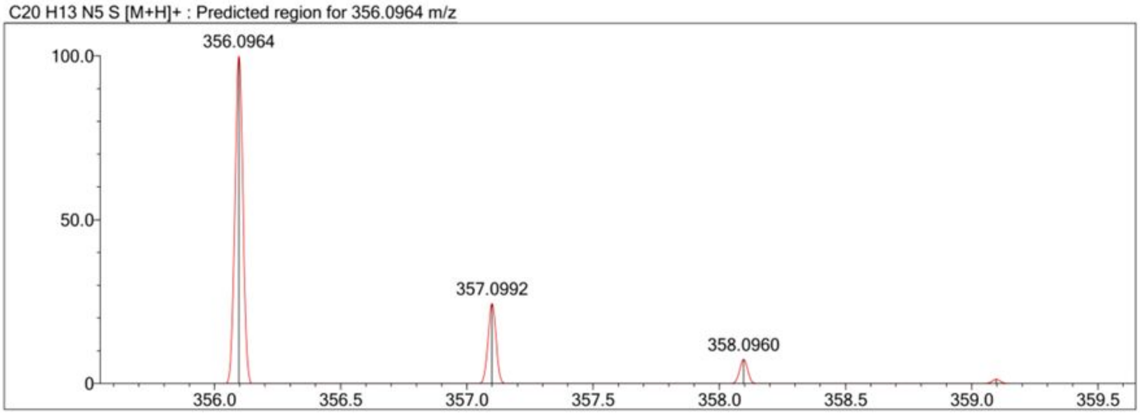

Rank	Score	Formula (M)	Ion	Meas. m/z	Pred. m/z	Df. (mDa)	Df. (ppm)	Iso	DBE
1	10.32	C20 H13 N5 S	[M+H]+	356.0948	356.0964	-1.6	-4.49	11.30	17.0

Figure S24. HRMS spectra of compound **2h**

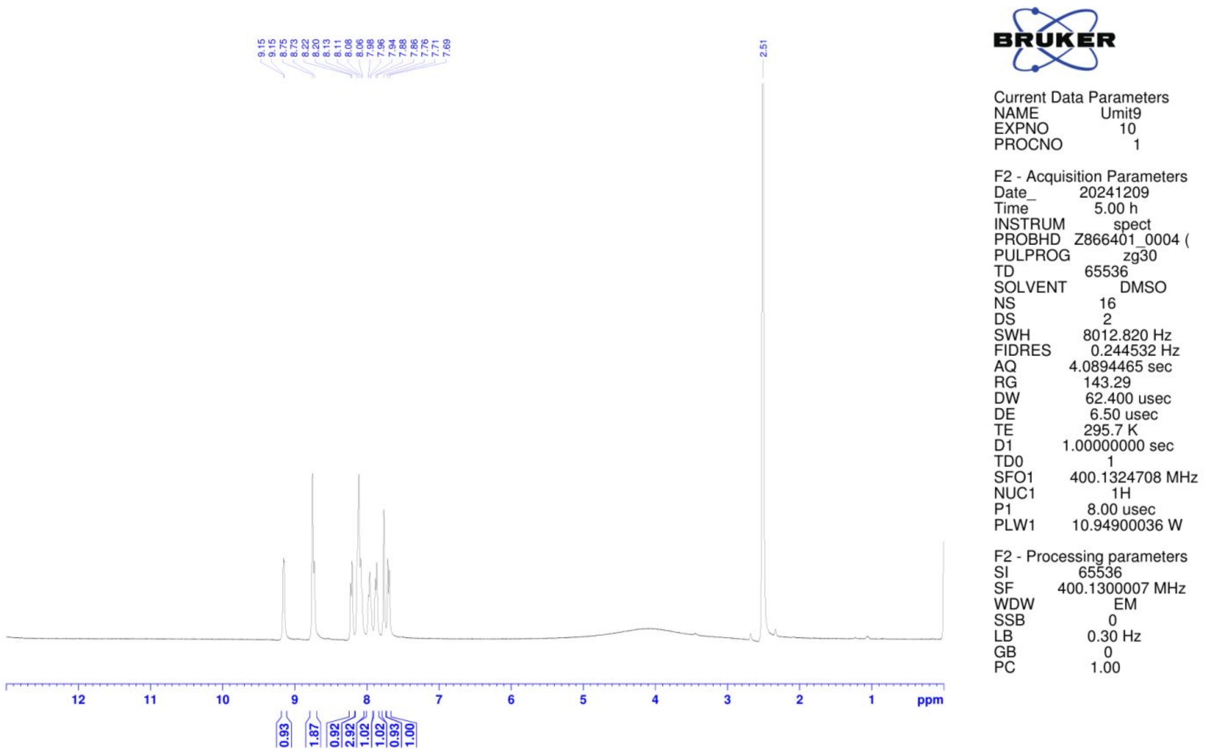

Figure S25. ^{1}H-NMR spectra of compound **2i**

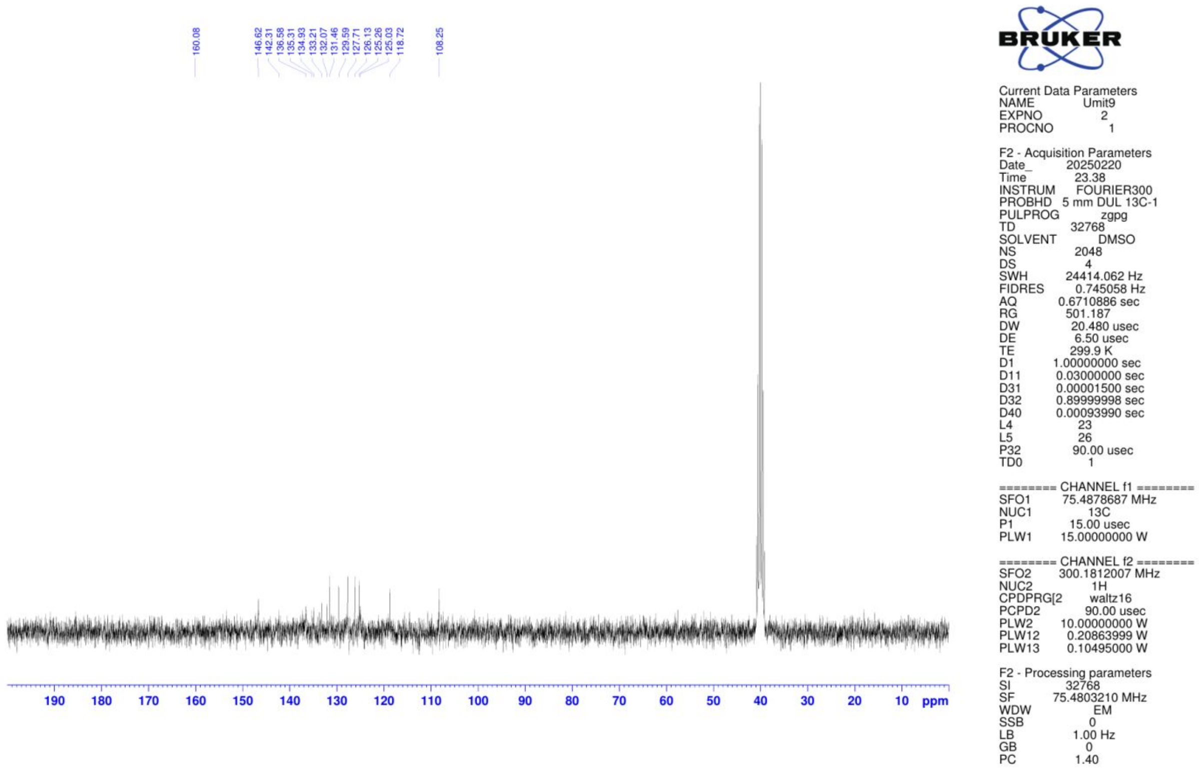

Figure S26. ^{13}C-NMR spectra of compound **2i**

Data File: C:\LabSolutions\Data\Analiz\Derya\Umit-9_48.lcd

Elmt	Val.	Min	Max	Elmt	Val.	Min	Max	Elmt	Val.	Min	Max	Elmt	Val.	Min	Max	Use Adduct
H	1	7	25	O	2	0	3	S	2	0	1	Ru	2	0	0	H
C	4	7	30	F	1	0	0	Cl	1	0	2	Pd	2	0	0	
N	3	2	5	P	3	0	0	Br	1	0	0	I	3	0	0	

Error Margin (ppm):	5	DBE Range:	12.0 - 19.0	Electron Ions:	both
HC Ratio:	unlimited	Apply N Rule:	yes	Use MSn Info:	yes
Max Isotopes:	5	Isotope RI (%):	1.00	Isotope Res:	9000
MSn Iso RI (%):	10.00	MSn Logic Mode:	AND	Max Results:	50

Event#: 1 MS(E+) Ret. Time : 2.120 Scan# : 319

Measured region for 399.0247 m/z

C19 H12 N4 S Cl2 [M+H]+ : Predicted region for 399.0232 m/z

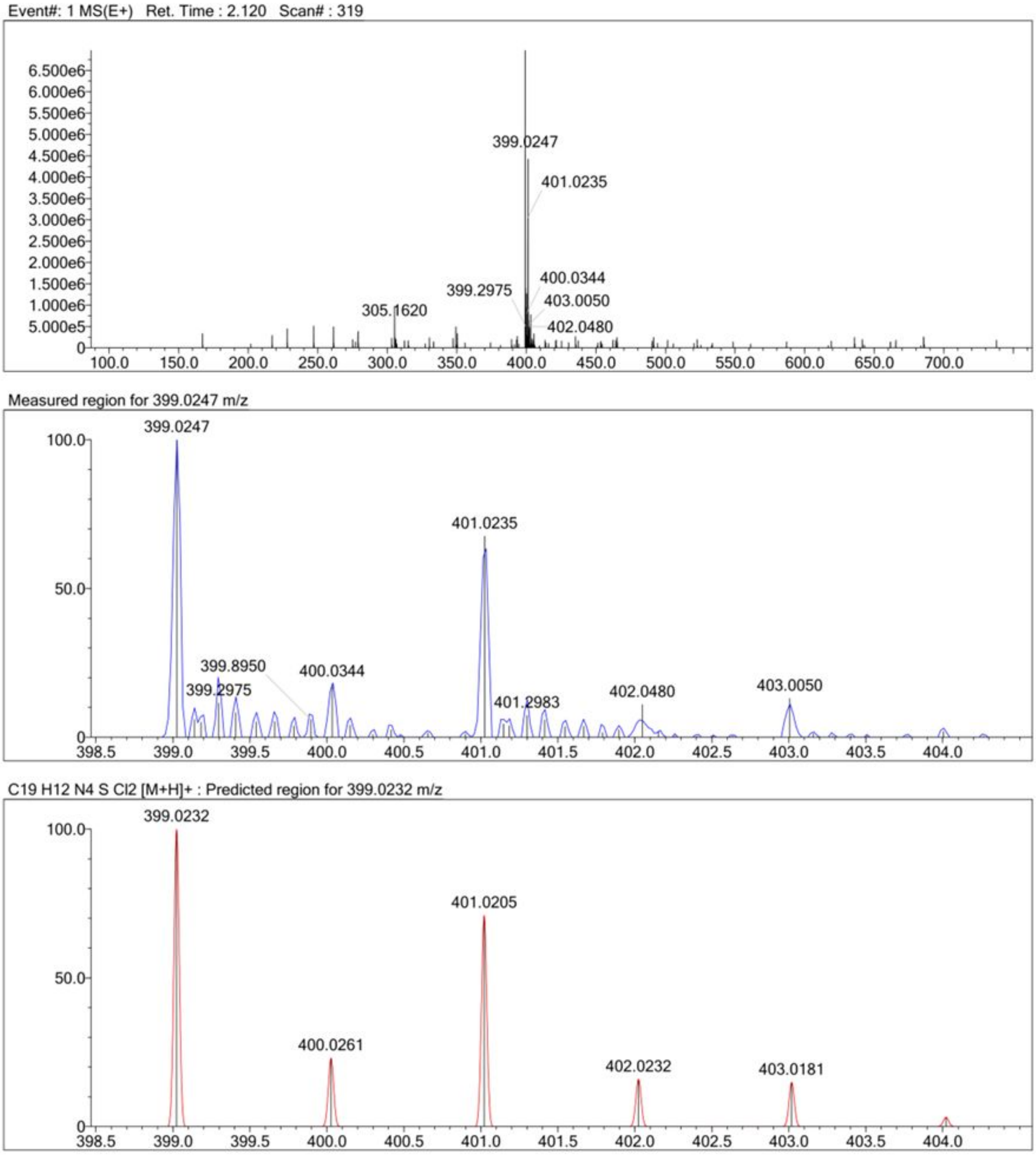

Rank	Score	Formula (M)	Ion	Meas. m/z	Pred. m/z	Df. (mDa)	Df. (ppm)	Iso	DBE
1	68.49	C19 H12 N4 S Cl2	[M+H]+	399.0247	399.0232	1.5	3.76	73.57	15.0

Figure S27. HRMS spectra of compound **2i**

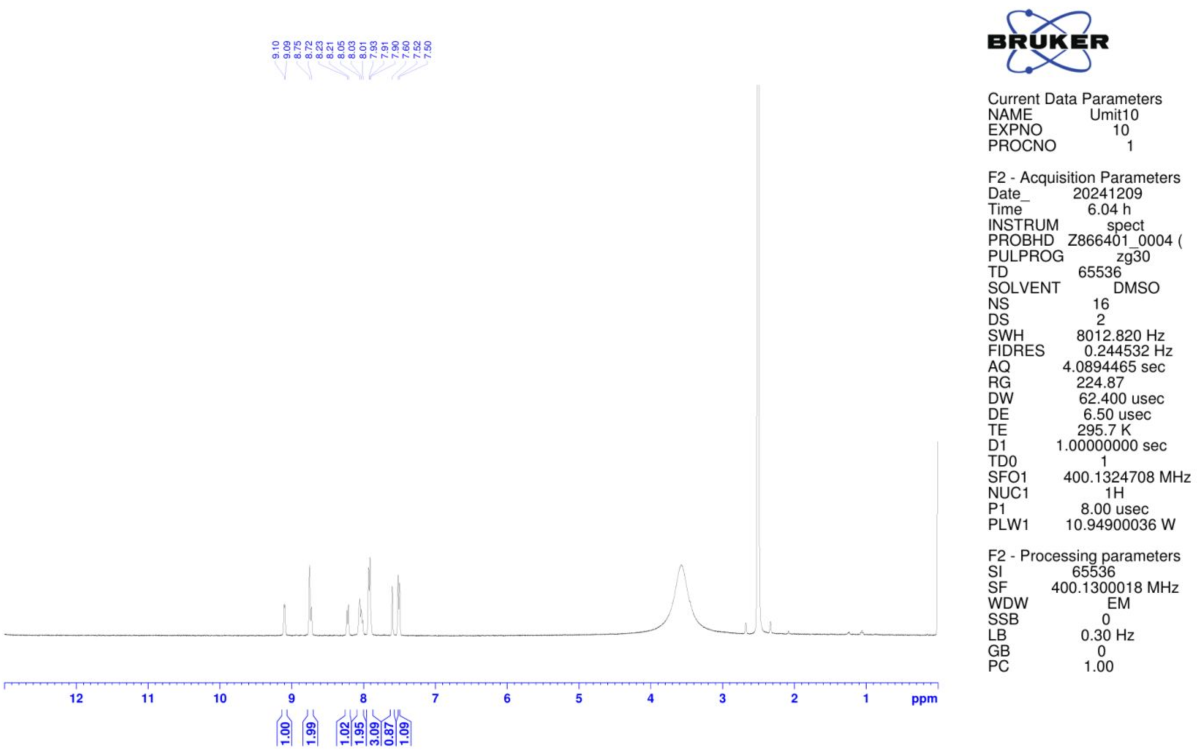

Figure S28. ^{1}H-NMR spectra of compound **2j**

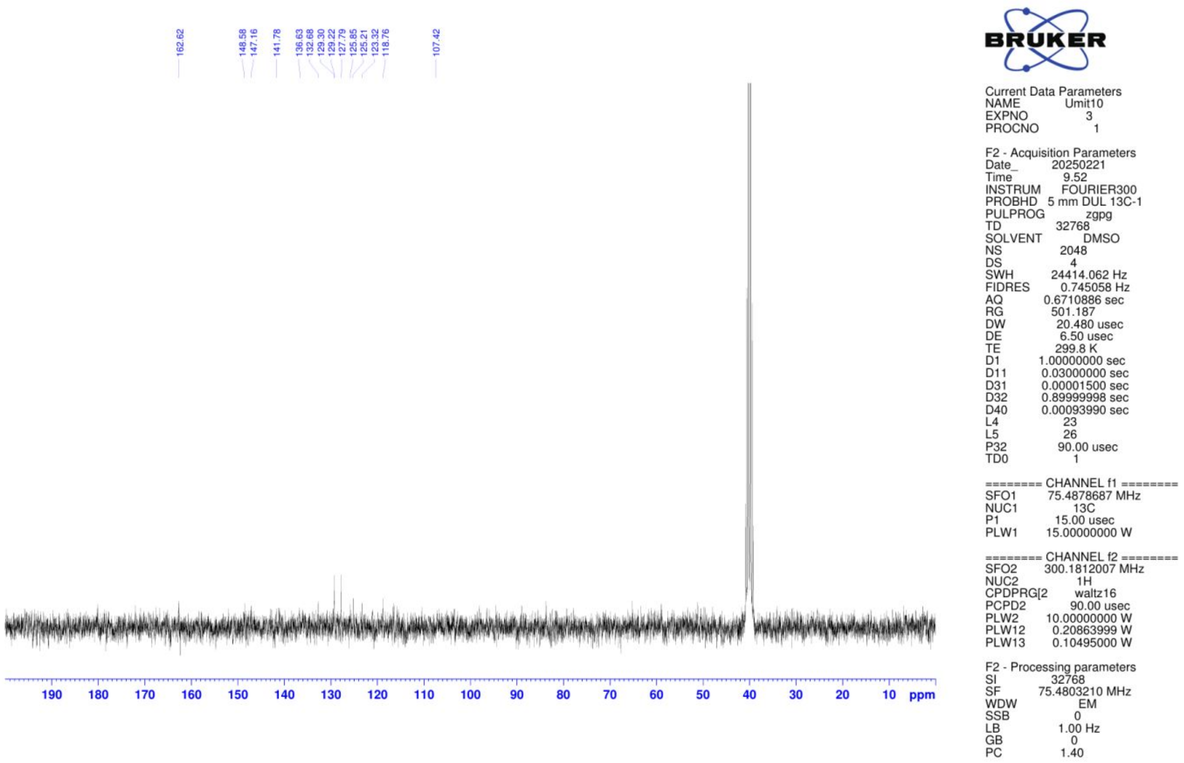

Figure S29. ^{13}C-NMR spectra of compound **2j**

Data File: C:\LabSolutions\Data\Analiz\Derya\umit-10_54.lcd

Elmt	Val.	Min	Max	Elmt	Val.	Min	Max	Elmt	Val.	Min	Max	Elmt	Val.	Min	Max	Use Adduct
H	1	10	23	O	2	0	3	S	2	0	2	Ru	2	0	0	H
C	4	12	22	F	1	0	0	Cl	1	2	2	Pd	2	0	0	
N	3	0	5	P	3	0	0	Br	1	0	0	I	3	0	0	

Error Margin (ppm):	5	DBE Range:	5.0 - 35.0	Electron Ions:	both
HC Ratio:	unlimited	Apply N Rule:	yes	Use MSn Info:	yes
Max Isotopes:	5	Isotope RI (%):	1.00	Isotope Res:	9000
MSn Iso RI (%):	10.00	MSn Logic Mode:	AND	Max Results:	50

Event#: 1 MS(E+) Ret. Time : 2.067 -> 2.173 Scan# : 311 -> 327

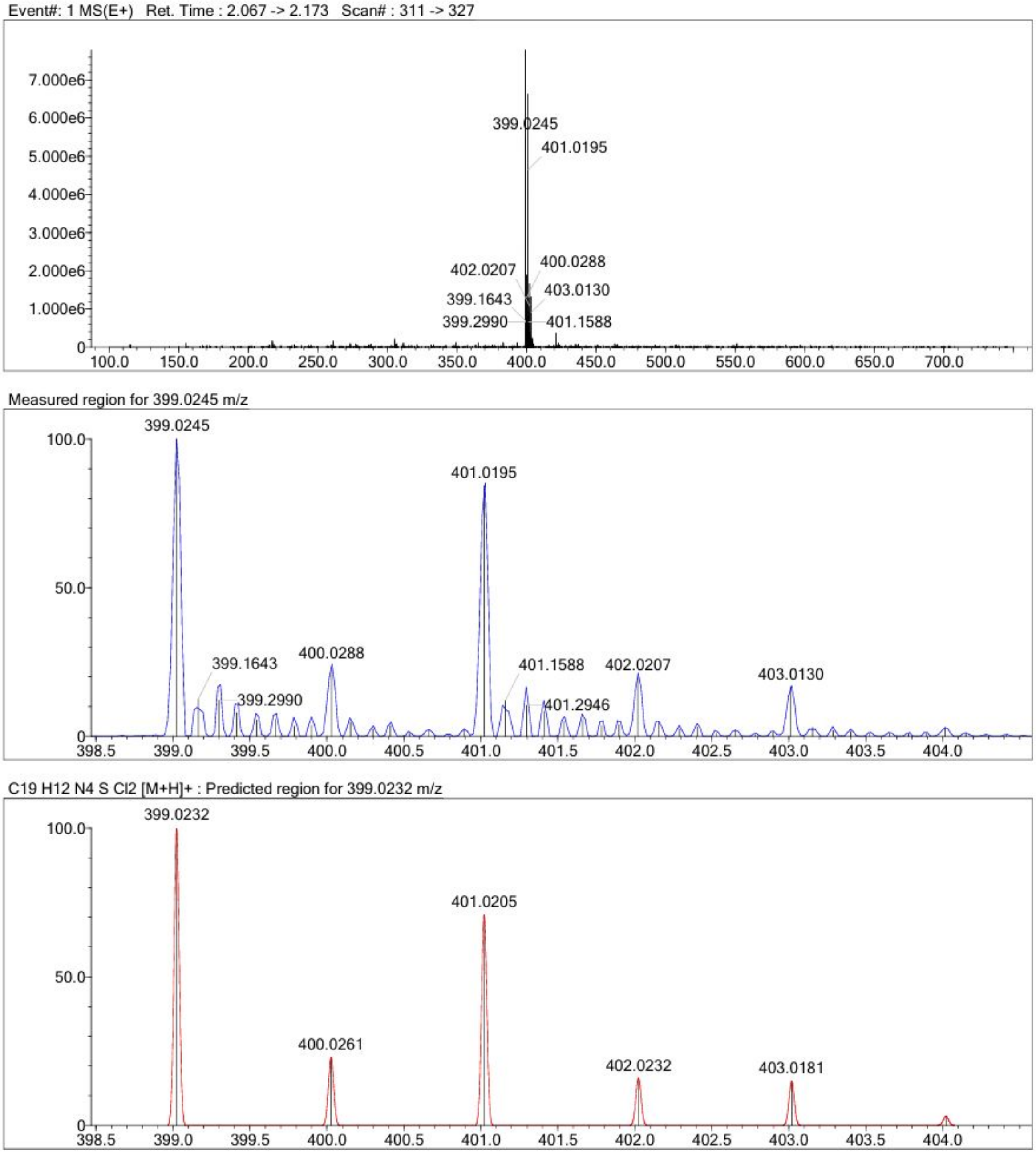

Rank	Score	Formula (M)	Ion	Meas. m/z	Pred. m/z	Df. (mDa)	Df. (ppm)	Iso	DBE
1	77.59	C19 H12 N4 S Cl2	[M+H]+	399.0245	399.0232	1.3	3.26	82.24	15.0

Figure S30. HRMS spectra of compound **2j**

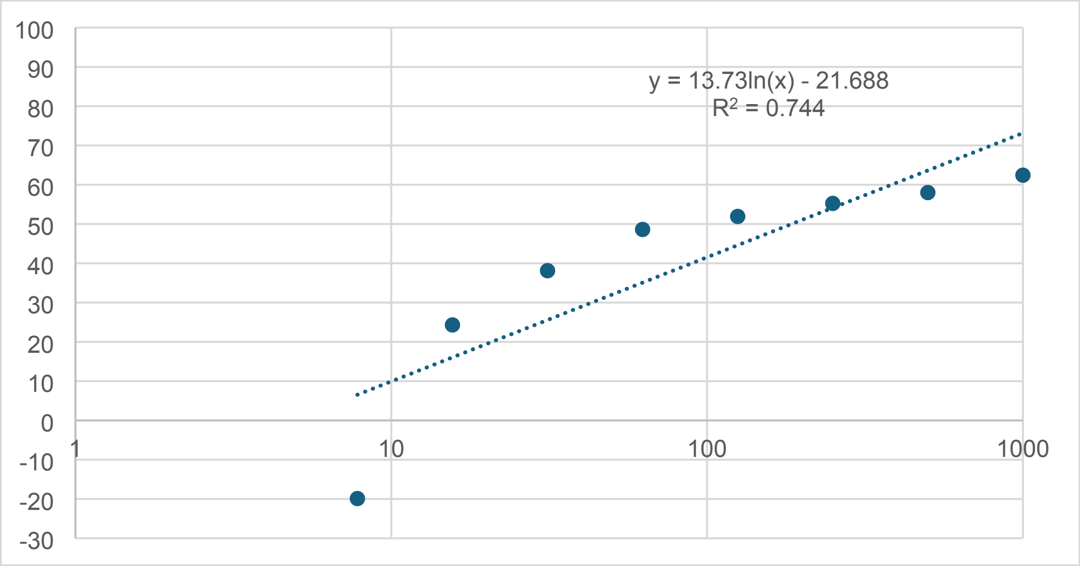

Figure S31. IC_{50} graphic of compound **2a** against A549

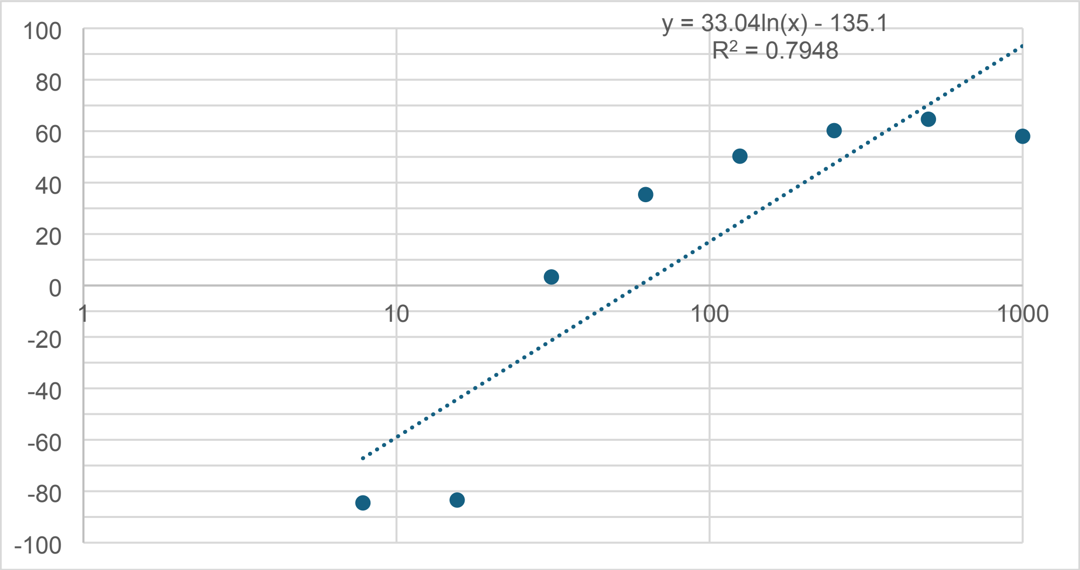

Figure S32. IC_{50} graphic of compound **2b** against A549

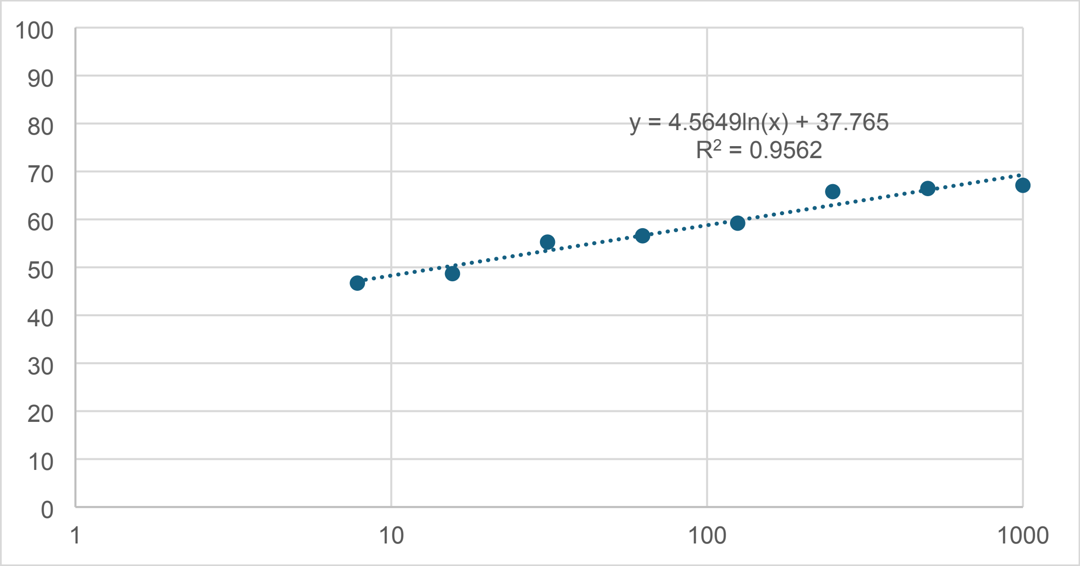

Figure S33. IC_{50} graphic of compound **2c** against A549

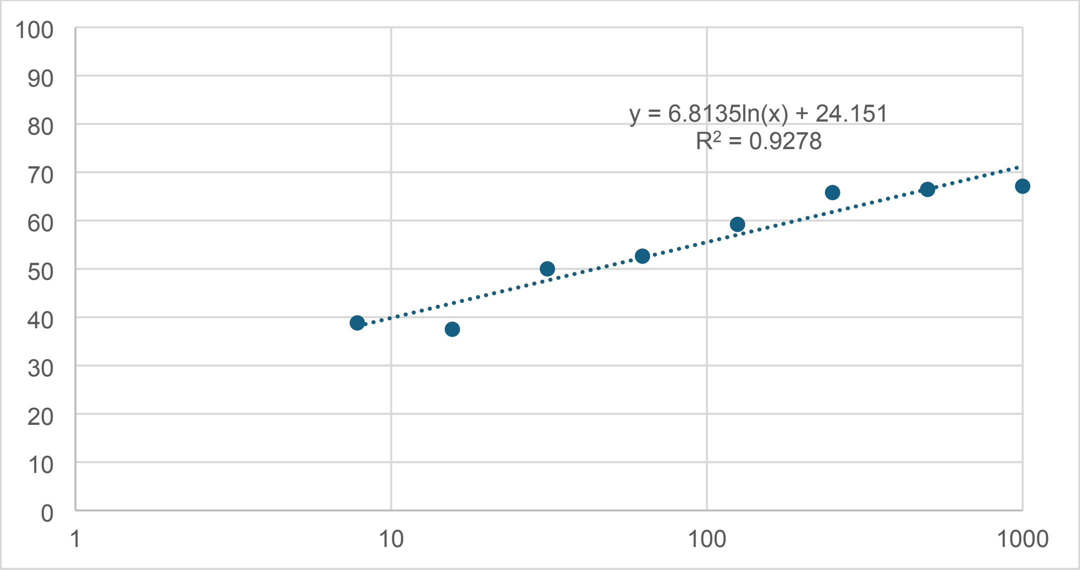

Figure S34. IC_{50} graphic of compound **2d** against A549

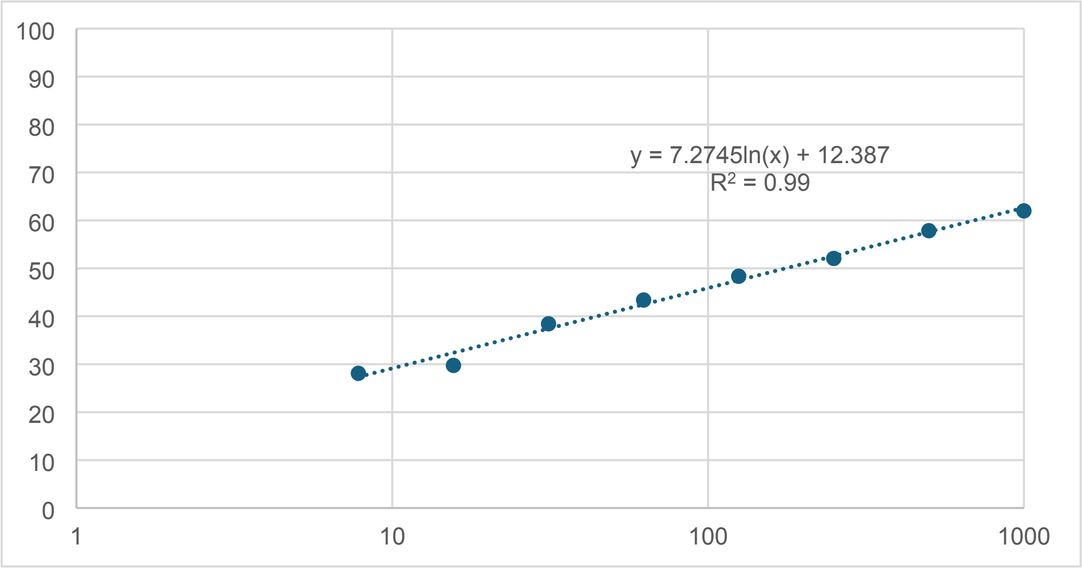

Figure S35. IC_{50} graphic of compound **2e** against A549

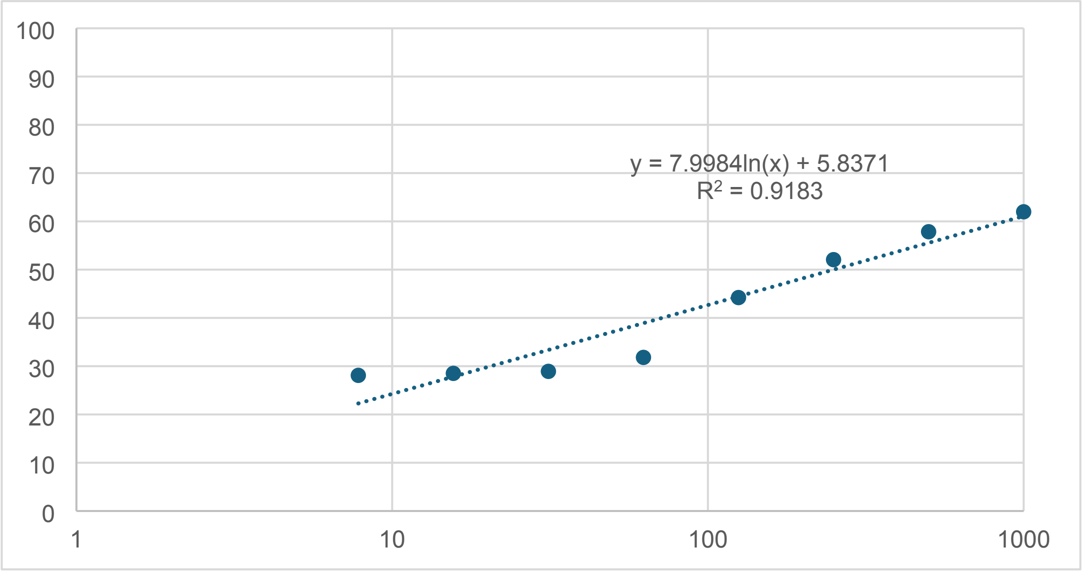

Figure S36. IC_{50} graphic of compound **2f** against A549

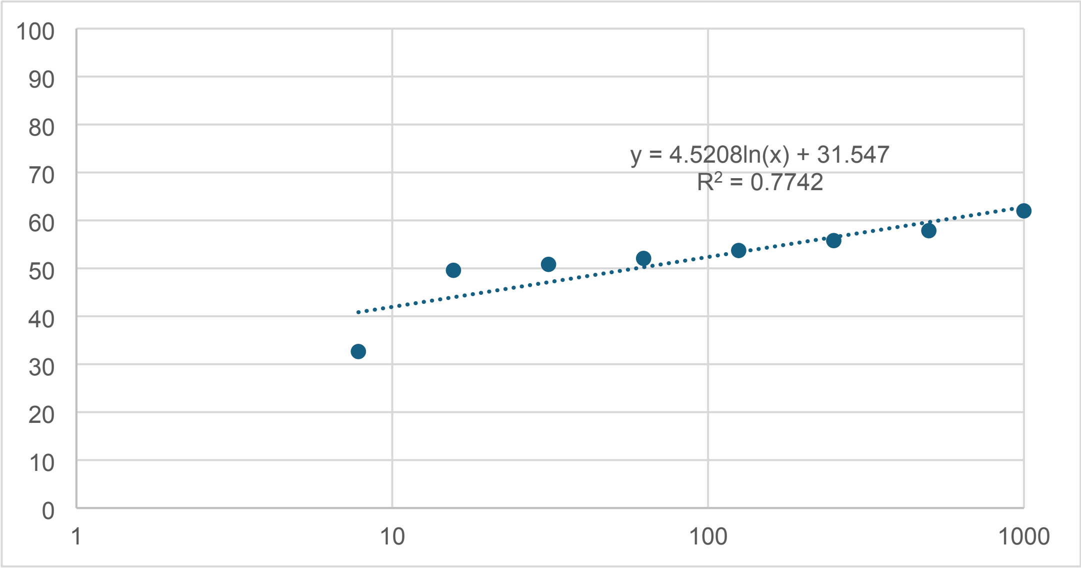

Figure S37. IC_{50} graphic of compound **2g** against A549

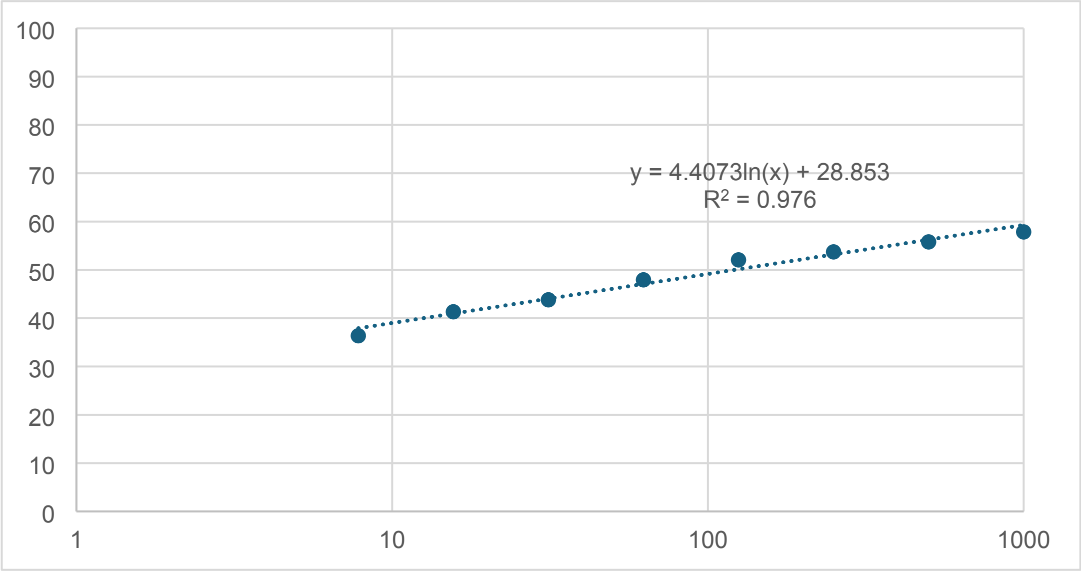

Figure S38. IC_{50} graphic of compound **2h** against A549

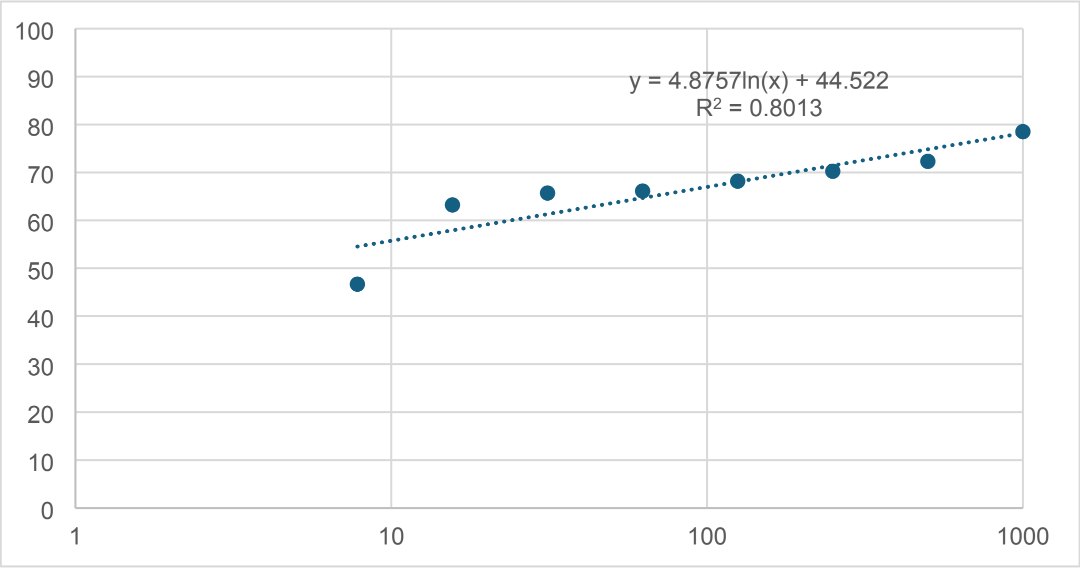

Figure S39. IC_{50} graphic of compound **2i** against A549

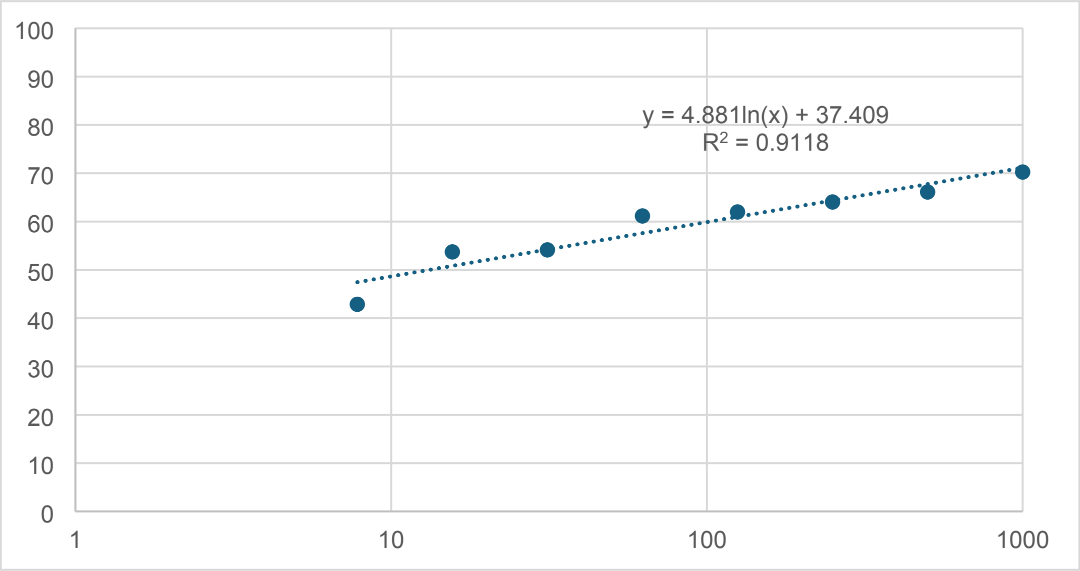

Figure S40. IC_{50} graphic of compound **2j** against A549

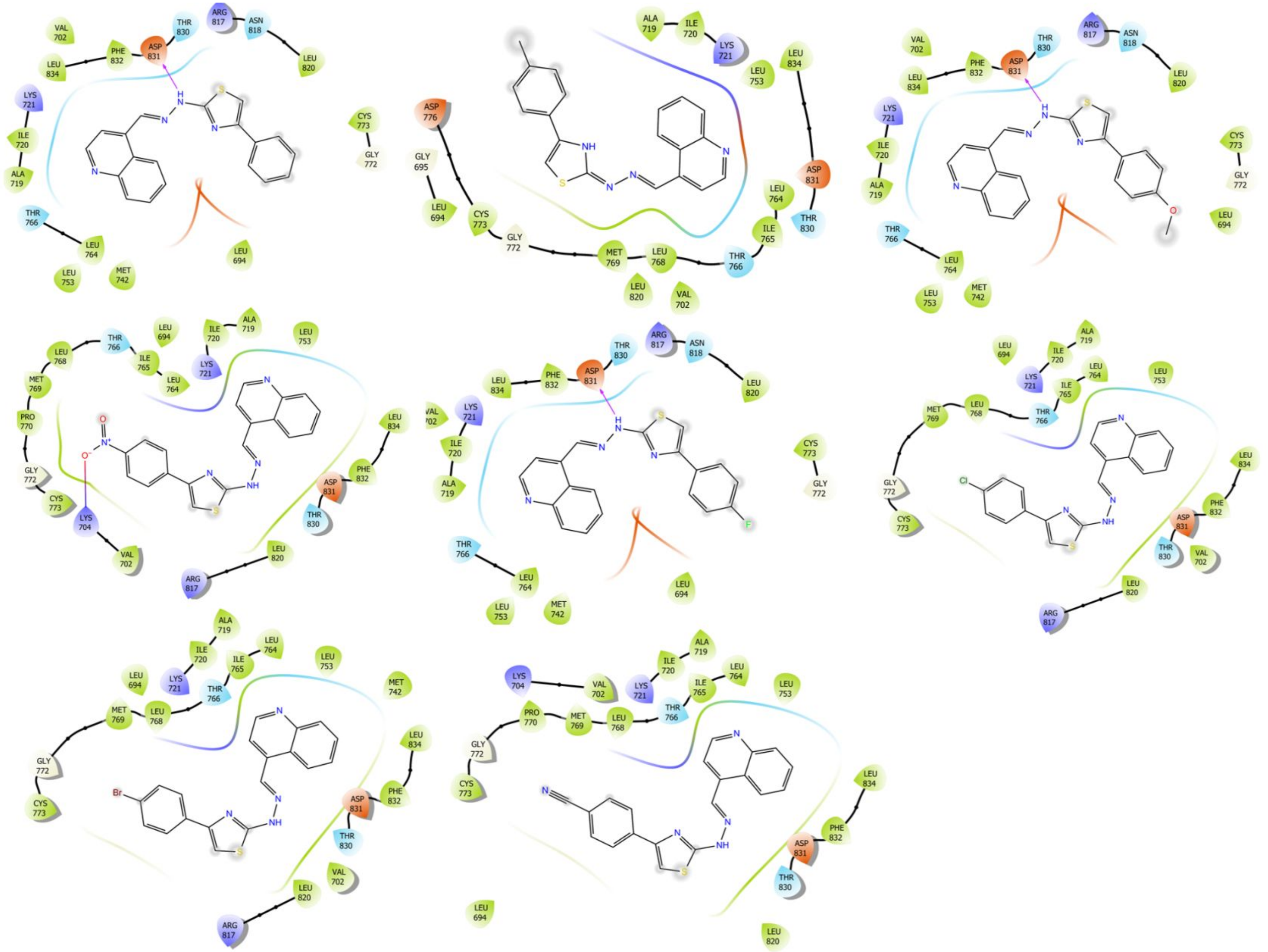

Figure S41. 2D Docking poses of compounds **2a-2h** with EGFR enzyme active site (PDB ID:4HJO)

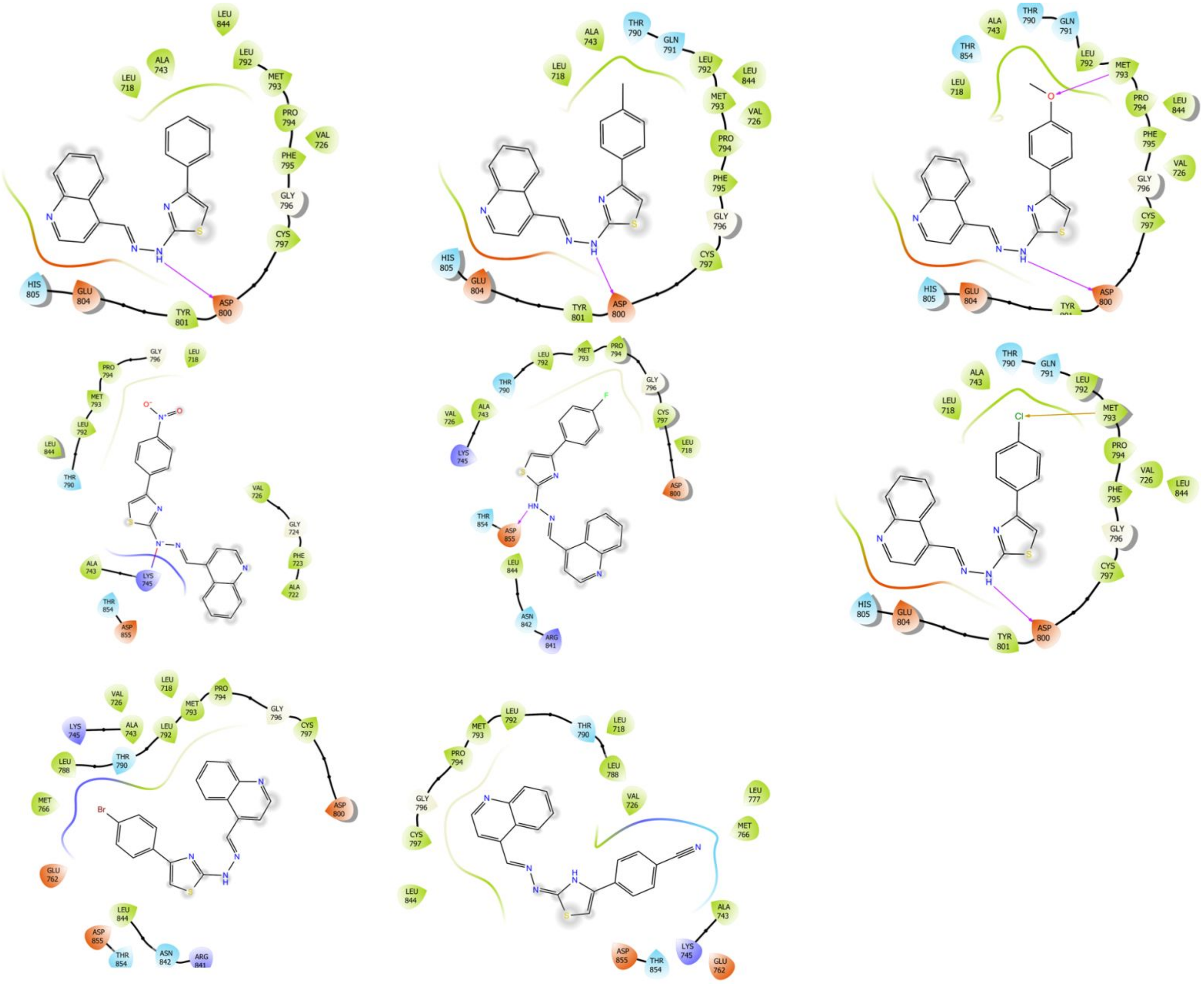

Figure S42. 2D Docking poses of compounds **2a-2h** with EGFR-L858R enzyme active site (PDB ID:2ITZ)

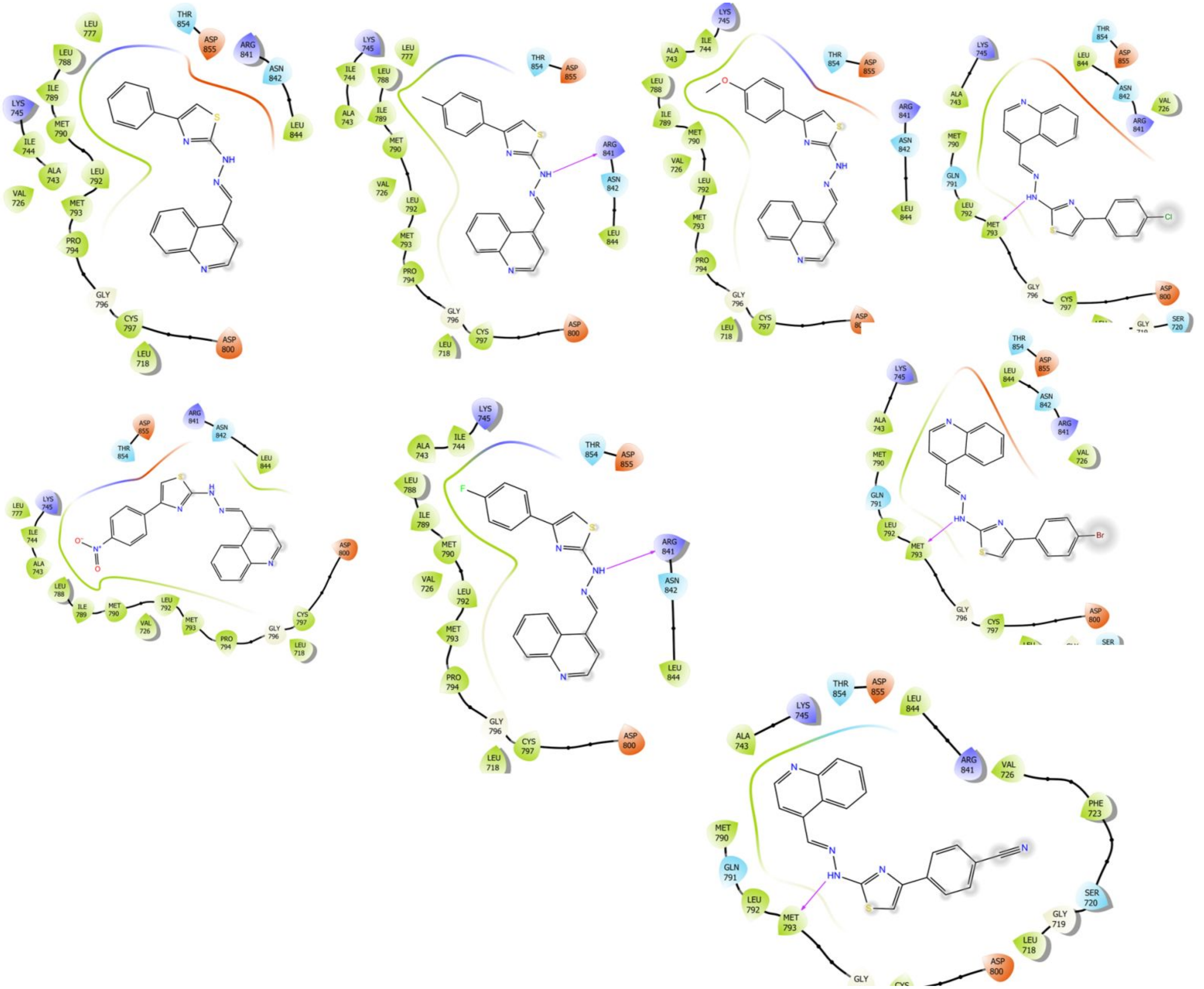

Figure S43. 2D Docking poses of compounds **2a-2h** with EGFR-L858R-T790M enzyme active site (PDB ID:4I22)
